# Novel Benzofuran-3-yl-methyl and Aliphatic Azacyclics: Design, Synthesis, and In Vitro and In Silico anti-Alzheimer Disease Activity Studies

**DOI:** 10.1021/acsomega.5c01432

**Published:** 2025-07-22

**Authors:** Büşra Gebeş-Alperen, Asaf Evrim Evren, Begüm Nurpelin Sağlik Özkan, Ahmet Cagri Karaburun, Nalan Gundogdu-Karaburun

**Affiliations:** Faculty of Pharmacy, Department of Pharmaceutical Chemistry, 52944 Anadolu University, 26470 Eskişehir, Turkey

## Abstract

Neurological disorders represent a significant burden on human health, particularly as global life expectancy continues to rise. Among these conditions, Alzheimer’s disease is notably prevalent. Of greater concern, if left untreated or unaddressed, Alzheimer’s disease can progress to dementia, leading to severe cognitive decline and a substantial reduction in quality of life. In this study, 15 novel benzofuran-azacyclic hybrids were designed and synthesized. The final compounds were evaluated for their inhibitory potency on AChE and BACE-1 enzymes, and in silico studies were performed to clarify their binding modes. Finally, structure–activity relationships (SARs) were proposed for future studies. The results indicated that the most promising compound is **4m**, which contains *N*-(2-hydroxyethyl)­piperazine and benzofuran moieties. These moieties effectively occupied the substrate channel of the AChE enzyme and the catalytic cleft of the BACE-1 enzyme. Additionally, compounds **4e** (benzyl piperidine) and **4h** (2-furoyl piperazine) showed dual inhibitory activity on both enzymes. In conclusion, the tubular form with a stopper group shows great potential for the treatment of Alzheimer’s disease, as it blocks the entrance cavity of the AChE active pocket for the substrate and increases the stability of the inactive BACE-1 enzyme. Moreover, electrolytes, specifically sodium ions in this case, play a crucial role in stabilizing the **4m**-BACE-1 protein complex. For further studies, we suggest that the tubular form with a stopper can serve as a potential pharmacophore and an appropriate starting point for drug development.

## Graphical abstract

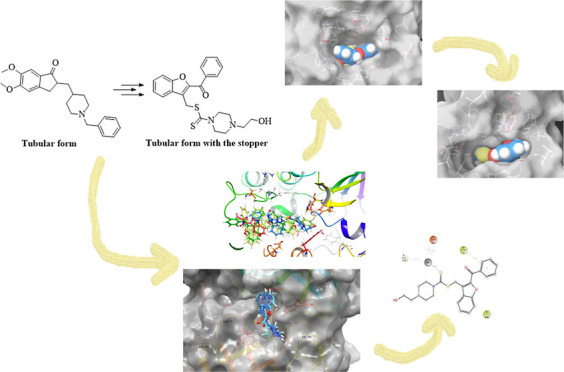

## 1 Introduction

Developments in technology and innovations in health sciences have significantly contributed to the extension of average human life. The recorded life expectancy at birth increased from 44 years in Sweden in 1840 to 82 years in Japan in 2005.^1^ As life expectancy has increased, age-related health issues have become more prominent and are now major global concerns. These include cancer and neurological disorders. Unfortunately, treatment options are mostly palliative and cannot cure these diseases or eliminate their underlying causes.^2^ Therefore, if a patient is not diagnosed in the early stages of the disease, the available treatment options are limited or even ineffective. To address this issue, researchers have aimed to develop dual inhibitors targeting both AChE and β-secretase.^3–5^ This approach holds promise for treating patients at both early and late stages of disease.

Based on this background, in this study we focused on benzofuran–piperazine analogs to fight against Alzheimer’s Disease. The benzofuran ring system has been investigated for the treatment of various diseases such as cancer,^6–8^ diabetes,^9^ soreness,^10^ anxiety,^11^ depression,^12^ Parkinson disease^13^ and also various infections.^14,15^ As known, benzofuran was chosen as a bioisosteric alternative to the indanone ring present in donepezil and galantamine, with the aim of exploring structural diversity while retaining interaction potential with the cholinesterase active site. This makes it an attractive scaffold for researchers developing anti-Alzheimer’s disease agents. Because of this bioisosterism, benzofuran derivatives have been explored for their potential to inhibit AChE^16,17^ and BACE-1 ^18,19^ enzymes or both of them at the same time,^20,21^ as same as moracin analogs.^22^ These studies suggest that the benzofuran ring is a valuable pharmacophore worth investigating for dual activity against both AChE and BACE-1.

Furthermore, aliphatic azacyclic structures such as piperidine, morpholine, and piperazine rings also exhibit anticancer,^23,24^ antidiabetic,^25,26^ analgesic,^27,28^ anxiolytic and antidepressant.^29,30^ These ring systems are commonly found in neurological drugs, as illustrated in Scheme 1. Additionally, piperidine, piperazine, and morpholine derivatives have been reported to show dual inhibitory activity on AChE and BACE-1 enzymes.^31–40^

**Scheme 1:**
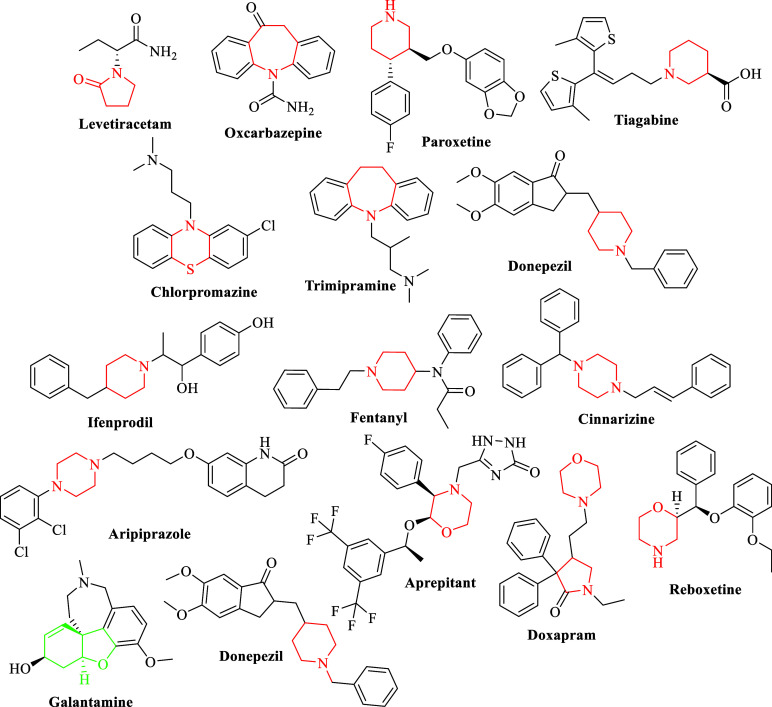
Drugs Used Clinically Including Aliphatic Azacyclic Moieties, and Donepezil and Galantamine^a^

a Azacyclic zstructures are represented by red, benzofuran moiety is represented by green.

In light of the above information, to explore structural diversity while retaining interaction potential with the enzyme active sites, 15 novel benzofuran and aliphatic azacyclic hybrids were synthesized by linking them through a dithiocarbamate moiety. The structures of the final compounds were confirmed using HRMS, as well as ^1^H and ^13^C NMR spectroscopy. Their anti-Alzheimer potential was evaluated against AChE, BuChE, and BACE-1 enzymes through both in vitro and in silico studies. Although the primary aim was to investigate the anti-Alzheimer disease activity of the newly designed benzofuran–azacyclic hybrids, we were also interested in examining the inhibitory effect of the tubular form modified with a “stopper” (The benzoyl moiety of the final compounds is positioned at the entrance of the enzyme’s active site, where it acts as a steric barrier, similar to how a wine stopper seals a bottle). This novel configuration may offer an alternative to conventional tubular drugs, with the added stopper potentially enhancing pharmacological performance. Additionally, structure–activity relationships (SARs) were discussed to clarify the impact of structural features on biological activity.

## 2 Results and Discussion

### 2.1 Chemistry

The synthetic route for the final compounds is illustrated in Scheme 2. The process involved three main steps. First, a ring-closure reaction between 2-bromoacetophenone and 2′-hydroxyacetophenone was carried out to obtain 2-benzoyl-3-methylbenzofuran (compound 1). In the second step, compound 1 was brominated using *N*-bromosuccinimide (NBS) to introduce a bromomethyl group at the 3-position of the benzofuran ring, yielding compound 2. In the final step, compound 2 was reacted with various dithiocarbamate derivatives under S_N_2 conditions to yield the target compounds.

**Scheme 2:**
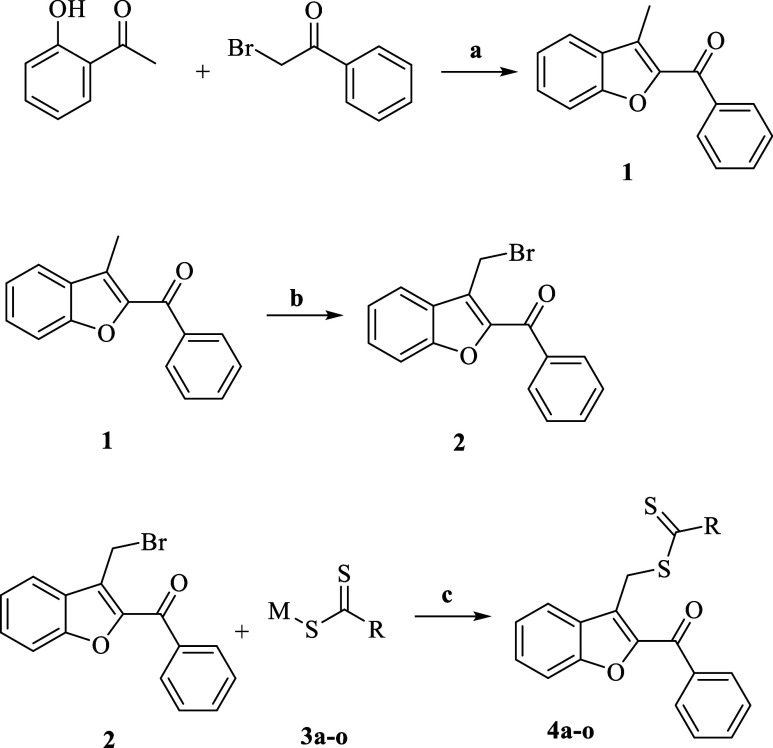
General Synthesis Diagram^a^

a Reaction condition: (a) K_2_CO_3_/CH_3_CN/reflux; (b) NBS/Benzoyl peroxide/CCl_4_/reflux; (c) Acetone, rt (**M**: Na or K).

The synthesized compounds are presented in Table 1. Based on IR spectral analysis, the aromatic C–H stretching vibrations appeared in the range of 2904–3089 cm^–1^. The carbonyl (C=O) stretching band of the benzoyl group located at the 2-position of the benzofuran ring was observed between 1633–1645 cm^–1^. The C=C, C=S, and C–O stretching bands were detected in the ranges of 1552–1597 cm^–1^, 1267–1292 cm^–1^, and 1215–1232 cm^–1^, respectively. Furthermore, characteristic peaks of monosubstituted benzene rings were observed at 715–758 cm^–1^ and 677–709 cm^–1^ as two distinct peaks, while the 1,4-disubstituted benzene ring showed a single peak in the range of 881–887 cm^–1^.

**Table 1:**
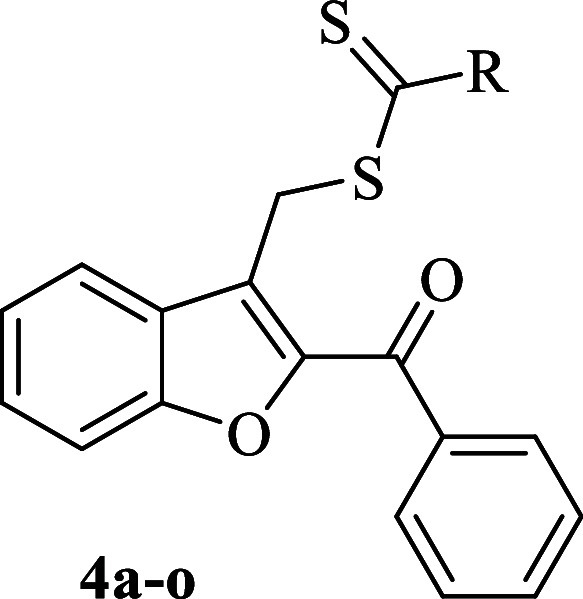

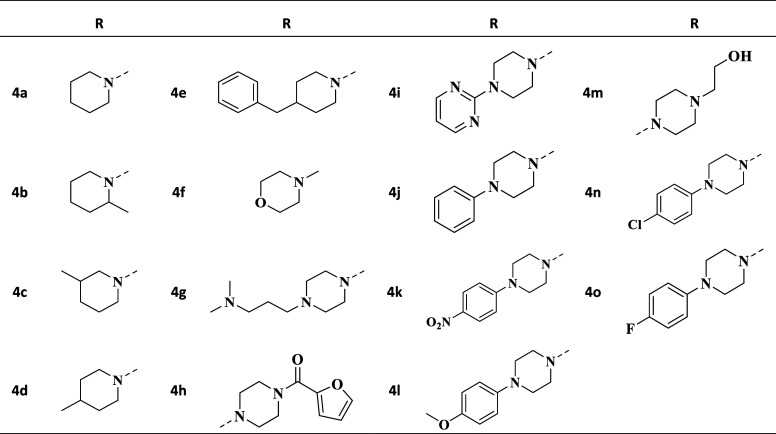
Core Structure and R Groups

In the ^1^H NMR spectra, the methylene protons (−CH_2_–S−) of the common structure appeared as a singlet between 5.05–5.10 ppm. The aliphatic −CH_2_– signals attributed to the piperazine ring were observed in the range of 4.00–4.30 ppm. Aromatic protons resonated between 6.64–8.39 ppm.

In the ^13^C NMR spectra, the common and consistent carbon signals of all derivatives were evaluated. The total number of carbon peaks in each spectrum matched the expected values. For compound **4o**, which contains a fluorine atom as a substituent, additional splitting was observed. This is attributed to C–F couplings, which caused splitting of the carbon signals directly bonded to fluorine as well as those adjacent to it, as previously reported in the literature. Peaks corresponding to S–CH_2_ and C=S, which confirm the ester-like structure of the dithiocarbamate moiety, were detected in the ranges of 30.42–32.09 ppm and 193.20–195.12 ppm, respectively.

### 2.2 Cholinesterase Activities

Inhibition % and IC_50_ doses of the final compounds and standard drugs were displayed in Tables 2 and 3, respectively.

**Table 2:**
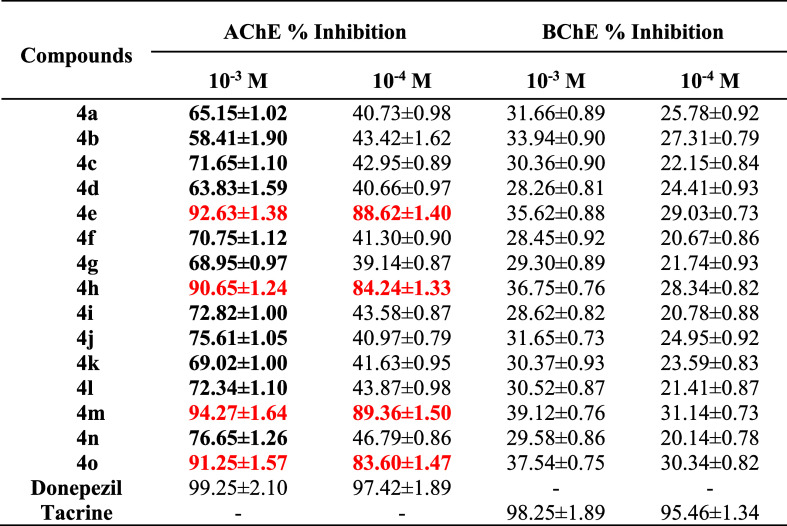
% Inhibition of the Synthesized Compounds, Donepezil and Tacrine against AChE and BChE

**Table 3:**
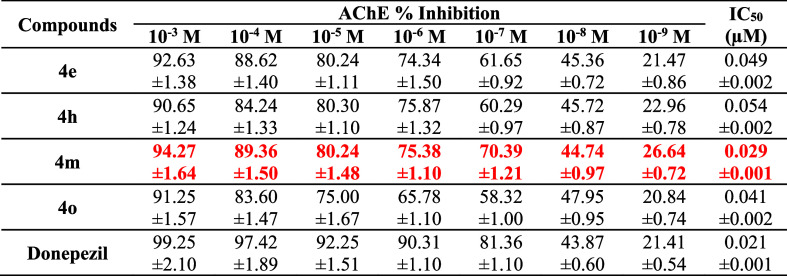
IC_50_ Values of **4e**, **4h**, **4m** and **4o** and Donepezil against AChE

According to the preliminary screening tests (at 10^–3^ and 10^–4^ M concentration) on AChE and BChE enzymes, compounds **4e**, **4h**, **4m** and **4o** showed better anti-AChE activity than other synthesized analogs and similar activity to donepezil, that is why they were evaluated in various concentrations (10^–3^–10^–9^ M) and then calculated their IC_50_ values. However, none of the final compounds showed an inhibition profile better than or similar to tacrine on BChE.

According to IC_50_ doses, compound **4m** (2-hydroxyethyl) showed better activity than other three compounds (**4e**, **4h**, and **4o**), but it is not better as donepezil. The less active compound among them is **4h**, which has furoyl piperazine moiety.

The findings indicated that piperidine substituted with bulky groups exhibited higher activity compared to those with smaller substituents. In contrast, piperazine derivatives bearing groups capable of forming hydrogen bonds did not show significant activity on their own. Furthermore, it appears that such substituents must be positioned at a minimum of three carbon atoms away from the fourth nitrogen of the piperazine ring to exhibit activity. Although compound **4g** fits this structural description, its IC_50_ value was measured between 10^–3^ and 10^–4^ M, which classifies it as a weak inhibitor. Therefore, additional structural factors must be considered to better understand the structure–activity relationship (SAR). We propose that piperazine rings substituted with flexible groups, such as 2-hydroxyethyl or 3-(*N*,*N*-dimethyl)­propyl, may present a more polarized topological surface. This polarity may contribute to enhanced activity. In this context, one explanation could be that shorter carbon chains positively influence the polarization of the molecular surface. For instance, while compound **4m** effectively inhibited AChE at low concentrations, compound **4g** did not, possibly due to its longer chain. Another plausible explanation is that the five-atom distance in compound **4g** from the azacyclic core prevents optimal positioning within the enzyme’s active site. Based on these observations, we suggest that a three- or four-atom distance from the azacyclic moiety is optimal when the substituents are straight-chain groups. However, if the substituents are aromatic rings, then a four- or five-atom linker (as observed in compounds **4e** and **4h**, respectively) results in higher activity.

### 2.3 β-Secretase Activities

The compounds showing the highest AChE inhibitory activity, along with reference drugs, were also evaluated for their effect on the BACE-1 enzyme. The results are presented in Table 4. Among all, compound **4m** demonstrated the highest inhibitory activity against BACE-1 (IC_50_: 0.134 ± 0.006 μM), performing better than donepezil (IC_50_: 0.110 ± 0.005 μM), although it was less potent than verubecestat (IC_50_: 0.031 ± 0.001 μM). Given that **4m**′s inhibition potency was approximately twice that of compounds **4e** and **4h**, and compound **4o** showed no significant inhibition, we propose the following: When the substituents on the azacyclic ring are flexible and small, the inhibitory activity is enhanced. However, if the substituent lacks the ability to form hydrogen bonds, a notable decrease in inhibition efficiency is observed.

**Table 4:** IC_50_ Values of **4e**, **4h**, **4m** and **4o** and Donepezil against BACE-1

compounds	β-secretase (BACE-1) IC_50_ (μM)
**4e**	0.134 ± 0.006
**4h**	0.155 ± 0.007
**4m**	0.084 ± 0.003
**4o**	>10
**donepezil**	0.110 ± 0.005
**verubecestat**	0.031 ± 0.001

### 2.4 Kinetic Studies of AChE Enzyme Inhibition

Enzyme kinetics studies were conducted to determine the mechanism of inhibition of AChE using a procedure similar to that of the inhibition assay for cholinesterase enzymes. These studies were performed with compound **4m**, which was found to be the most potent agent. Linear Lineweaver–Burk graphs were used to estimate the type of inhibition of this compound. The velocity curves of the substrates were recorded in the absence and presence of compound **4m**. This compound was prepared for enzyme kinetic studies at concentrations of IC_50_/2, IC_50_, and 2 × IC_50_. In each case, the initial velocity measurements were obtained at different substrate (ATC) concentrations ranging from 600 to 18.75 μM. To calculate the *K*_i_ (intercept on the *x*-axis) values of this compound value, the secondary plots of slope (*K*_m_/*V*_max_) versus varying concentrations (0, IC_50_/2, IC_50_, and 2 × IC_50_) were created. The graphical analyses of steady-state inhibition data for compound **4m** are shown in Figure 1.

**Figure 1:**
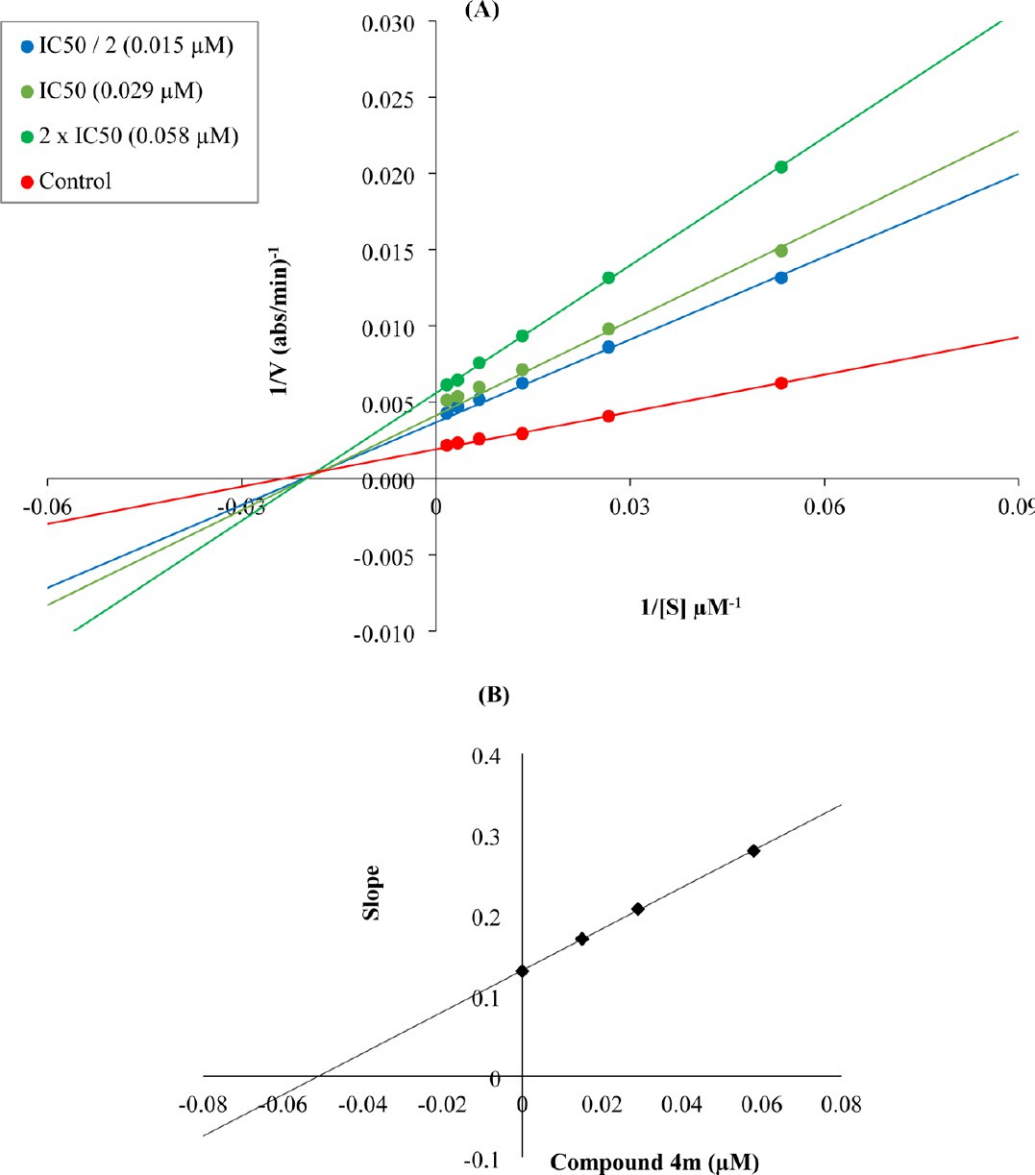
(A) Lineweaver–Burk plots for the inhibition of AChE by compound **4m**. [S], substrate concentration (μM); *V*, reaction velocity (1/*V* (abs/min)^−1^). Inhibitor concentrations are shown at the left. (B) Secondary plot for the calculation of the steady-state inhibition constant (*K*_i_) of compound **4m**. *K*_i_ was calculated as 0.051 μM.

According to the Lineweaver–Burk plots, the type of inhibition consists of two general classes: reversible or irreversible. Mixed-type, uncompetitive, competitive, and noncompetitive inhibition types are included in the reversible inhibition [1–3]. As seen in the Lineweaver–Burk plot of compound **4m** (Figure 1), a graph with lines that do not intersect at the *x*-axis or the *y*-axis was formed. This observation indicated that compound **4m** was a reversible and mixed-type inhibitor with similar inhibition features as the substrates. Furthermore, the *K*_i_ value of compound **4m** was calculated as 0.051 μM with the help of a secondary plot.

#### 2.4.1 Molecular Docking Studies on AChE

To understand binding mode, structure-based docking protocol was applied. According to docking studies (Figure 2), the settlement at the inhibitory cavity of all active compounds was found very similar, thus, 2-benzoylbenzofuran structure can be marked as a valuable pharmacophore core.

**Figure 2:**
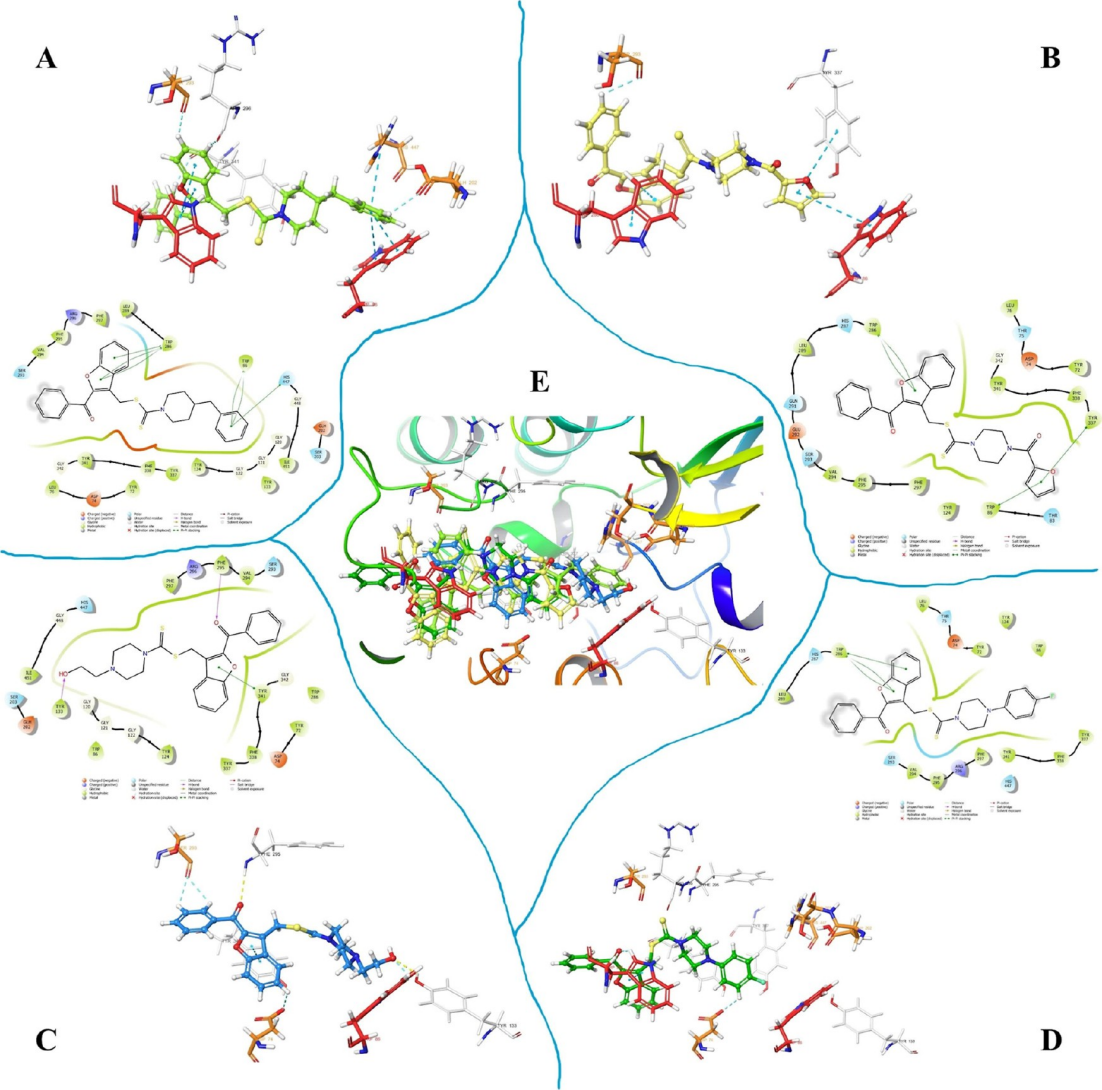
2D and 3D docking poses of **4e** (A), **4h** (B), **4m** (C), **4o** (D), and their superimposed (E) at AChE binding cavity (PDBID: 4EY7), respectively.

In fact, all compounds interacted with the Trp86 (catalytic active site, CAS) and Trp286 (peripheral anionic site, PAS) amino acid residues. Additionally, all compounds except **4o** formed interactions with Ser293, a residue located in the ATP-binding pocket. Since compound **4o** is slightly shorter than the other active derivatives, this provided a useful insight: introducing one or two sp^3^-hybridized carbon atoms between the piperazine and phenyl ring—as seen in compound **4e**—could potentially enhance its inhibitory activity by enabling deeper access into the CAS region. Indeed, similar to the structure of donepezil, benzyl piperazine (in this case, we propose 4-fluorobenzyl piperazine) could serve as an appropriate substituent. Compound **4e**, in fact, was designed as a structural analog of **4o** to explore this hypothesis. Although in vitro results indicated that **4o** was more effective than **4e**, compound **4e** formed more molecular interactions. Based on this, we suggest that ideal structures should incorporate benzyl piperazine analogs, such as the 4-fluorobenzyl-substituted piperazine. The most potent compound, **4m**, bearing a 2-hydroxyethyl piperazine moiety, was able to interact with key amino acids in the CAS region, particularly Trp86 (via aromatic hydrogen bonding) and Tyr133 (through hydrogen bonding). Another noteworthy observation is that compound **4h** lacks a positively charged nitrogen atom in its side chain, which likely results in unstable interactions with the enzyme, especially since this region of the binding pocket includes residues that can carry charge. To further elucidate the binding mode and gain deeper insight into the structure–activity relationship (SAR), molecular dynamics simulations (MDS) were performed using the compound **4m**—AChE complex as a representative model.

#### 2.4.2 Molecular Docking Studies on BACE-1 Enzyme

Docking poses of active compounds (**4e**, **4h** and **4m**) on β-secretase enzyme were shared in Figure 3.

**Figure 3:**
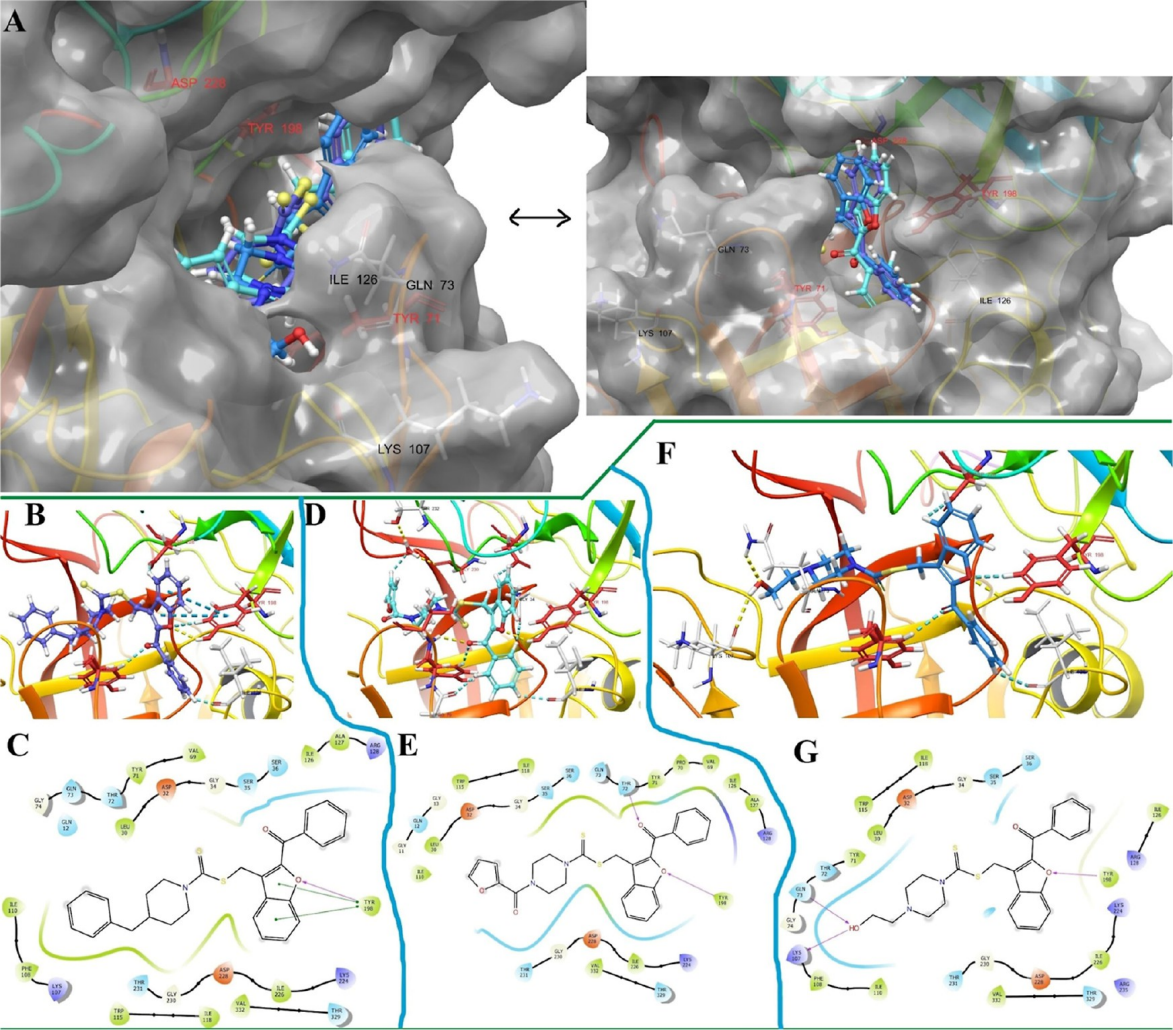
Superimposed of the active compounds from two opposite perspectives (A), and 2D and 3D docking poses of **4e** (B), **4h** (C), and **4m** (D) at BACE-1 catalytic cleft (PDBID: 2ZJM), respectively.

Three active compounds were found to fit well within the catalytic cleft of the β-secretase enzyme (Figure 3A). The common structural moiety, 1-benzofuran-2-yl­(phenyl)­methanone (BPM), interacted with Tyr198 via hydrogen bonding and π–π stacking, thus occupying the S2′ and S3′ subpockets, while the phenyl group extended into the S4′ subpocket. The dithiocarbamate groups of the compounds were positioned between Tyr71 and Asp228 and extended into the S1, S4, and S1′ pockets through their azacyclic moieties. Variations on this aliphatic ring were directed toward the S3 and S4 pockets and positioned accordingly. Based on these observations, we suggest that the nature of the substituents significantly influences the stabilization of the enzyme–inhibitor complex, and thereby, the overall biological activity. Specifically, in these pockets, the 2-hydroxyethyl group of compound **4m** formed direct hydrogen bonds with Gln73 and Lys107, whereas the furoyl group of compound **4h** interacted with Gly230 and Thr232 through water-mediated hydrogen bonds. In summary, we concluded that the BPM scaffold is highly effective, as it engages both the S2′ and S3′ subpockets and simultaneously interacts with three distinct loop-region amino acids. To gain deeper insight into the binding mechanism, molecular dynamics simulations were carried out using the compound **4m**–β-secretase complex as a representative model.

#### 2.4.3 Molecular Dynamics Simulation Studies on AChE

Stability-related plots are presented in Figure 4A–C. The average radius of gyration (*R*_g_) was approximately 4.8 Å, with the system reaching equilibrium and displaying minimal fluctuations after 12.8 ns. Notably, no drastic deviations were observed before this point, indicating that the system retained structural integrity throughout the simulation. The root-mean-square deviation (RMSD) values were as follows: 0.00–1.88 Å for the protein, 0.00–4.79 Å for the ligand aligned to the protein, and 0.00–2.06 Å for the ligand aligned to itself. These results demonstrate that the protein–ligand complex remained stable during the simulation period. The root-mean-square fluctuation (RMSF) values for amino acids in α-helices and β-strands remained below 0.8 Å, while loop-region residues involved in ligand interactions exhibited RMSF values below 1.0 Å. These fluctuations are within acceptable ranges, as supported by several previous studies.^41–44^

**Figure 4:**
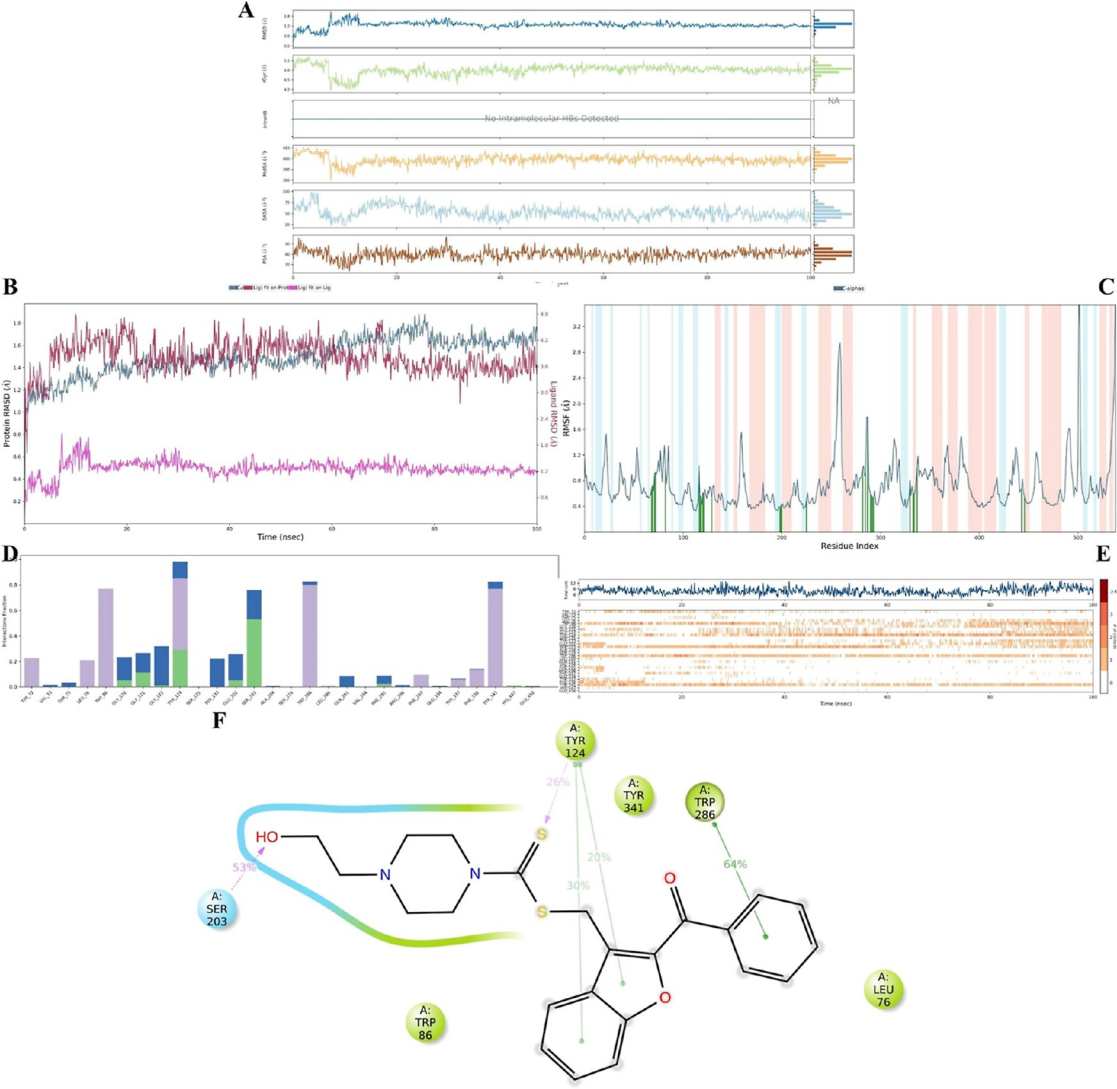
MDS result for the **4m**-AChE complex. (A–C) showed the complex stability; and (D–F) showed the interaction diagrams during 100 ns simulation. (A) **4m** properties (RMSD, *R*_g_, intraHB, MolSA, SASA, and PSA; (B) RMSD values (Protein; ligand fit on protein; and ligand fit on ligand); (C) RMSF diagram; (D) interaction fraction–residue diagram; (E) total connections-residues-time plot; (F) 2D interaction pose with connection strength (cut off = 0.2) at the active region).

After assessing the stability of the complex, the entire simulation was analyzed to understand the binding mode of the ligand–protein complex in relation to time and environmental changes. As shown in Figure 4D,E, the residues Trp86, Tyr124, Ser203, Trp286, and Tyr341 exhibited the most interactions compared to other active pocket amino acids. Notably, the interactions with Trp86 and Tyr341 decreased between 38 and 78 ns, likely due to weak contacts, as these interactions were primarily hydrophobic in nature. However, after 78 ns, the interaction frequencies with these residues increased again. We propose that the ligand’s activity was predominantly driven by interactions with Trp86, Ser203, and Trp286. Meanwhile, residues such as Gly120, Gly121, Gly122, Tyr124, Tyr133, and Glu202 played a crucial role in stabilizing the complex, preventing the ligand from dissociating from the substrate-binding region, and thereby ensuring the continued inhibitory effect. This stability was maintained despite intermittent disruptions in interactions with Trp86 (as observed in video 1, Figure 4). Notably, the *N*-(2-hydroxyethyl)­piperazine moiety of the ligand penetrated the CAS region of the AChE enzyme pocket, while the 1,1-diaryl methanone moiety occupied the PAS region, functioning as a stopper. Thus, the 1,1-diaryl methanone group effectively creates a barrier, preventing the entry of the substrate (acetylcholine) and water molecules into the active site (see video 1 and Figure 5).

**Figure 5:**
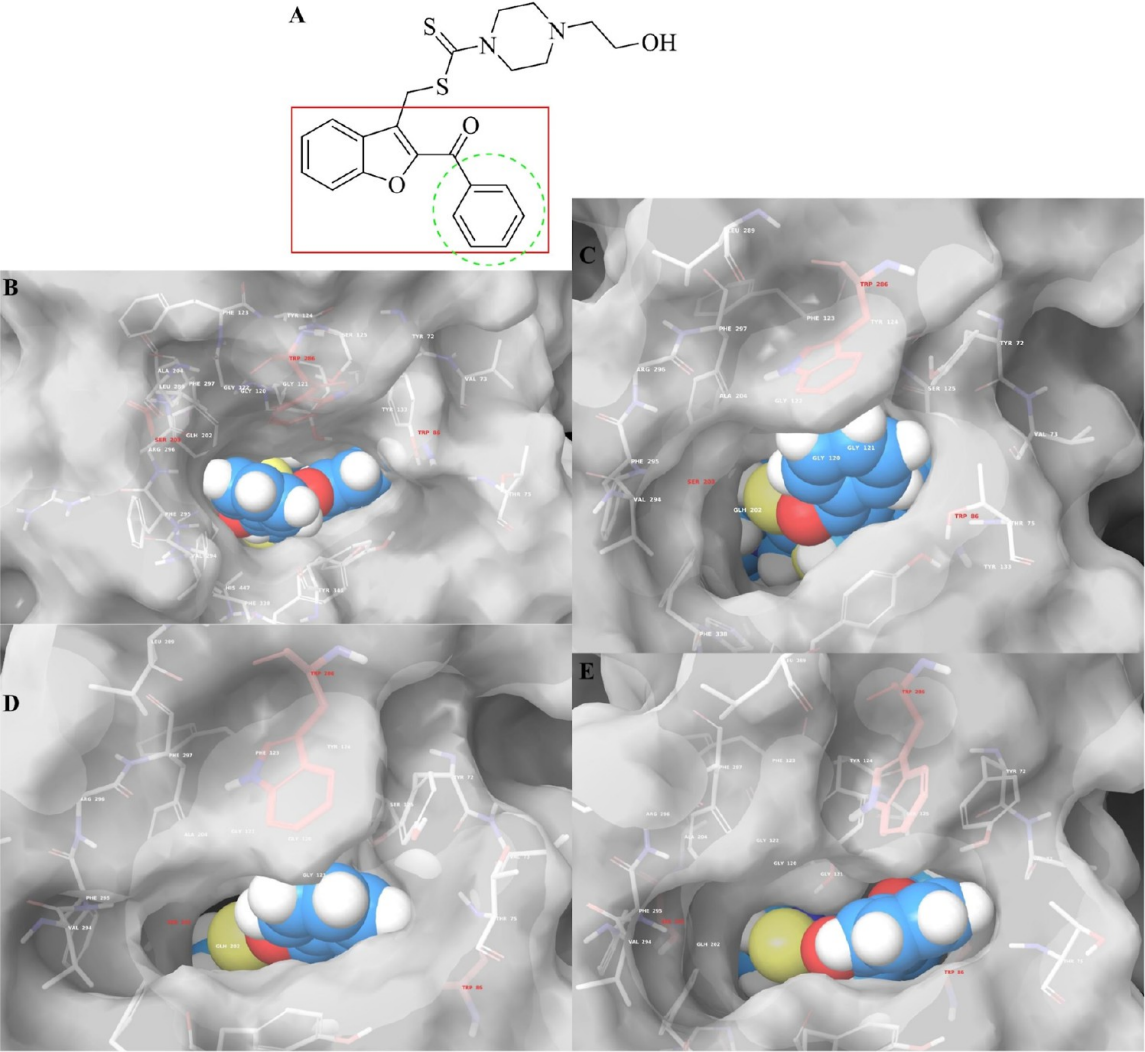
Representative snaps extracted from MDS of (B) 0.0 ns, (C) 33.3 ns, (D) 66.6 ns, and (E) 100.0 ns. The occlusion of the AChE substrate-binding channel induced by the presence of compound **4m** (its structure represented in (A)). Only interacted residues are displayed, and the red carbon residues represent mentioned as important ones in previous studies. White surface area (transparency front = 20, and back = 0) was used for rendering the AChE substrate-binding channels shape.

In conclusion, unlike the classic tubular structure of AChE inhibitors, experimental studies have suggested that the combination of a polarizable straight chain and rigid hydrophobic rings is an effective approach for AChE inhibition. As detailed in the in silico section, this combination is attributed to the polarization of the straight chain and the bulky hydrophobic rings of the compounds. The hydrophobic rings act as a barrier at the enzyme’s entrance, blocking substrate binding like wine stopper, while the polarized straight chain interacts with charged residues, stabilizing the complex. This stabilization is further supported by the hydrophobic moiety of compound **4m** through its interaction with Trp286 (as seen in video 1 and Figure 4C–E). Consequently, the in silico studies predicted possible binding modes, and these findings highlight that a group capable of forming hydrogen bonds can enhance the frequency of interactions in the CAS region. For future studies, the key points and suggestions outlined here should be considered when designing and developing new AChE inhibitors.

#### 2.4.4 MDS Studies on BACE-1

Similar to **4m**-AChE complex, **4m**-BACE-1 complex has a good stability profile as shown in Figure 6A–C. The overall rG value was calculated 4.7 Å and it did not show drastic changes during simulation time. The maximum RMSD value for protein was calculated as 2.29 Å. Similar to **4m**-AChE complex, the RMSF values of α-helix and β-strand region amino acids were under 0.8 Å while the RMSF values of loop region amino acids, if the ligand interacted with them, were observed under 1.5 Å. For this complex, all values are acceptable (as mentioned in MDS of AChE) and gesture that the stability of the **4m**-BACE-1 complex was not interrupted.

**Figure 6:**
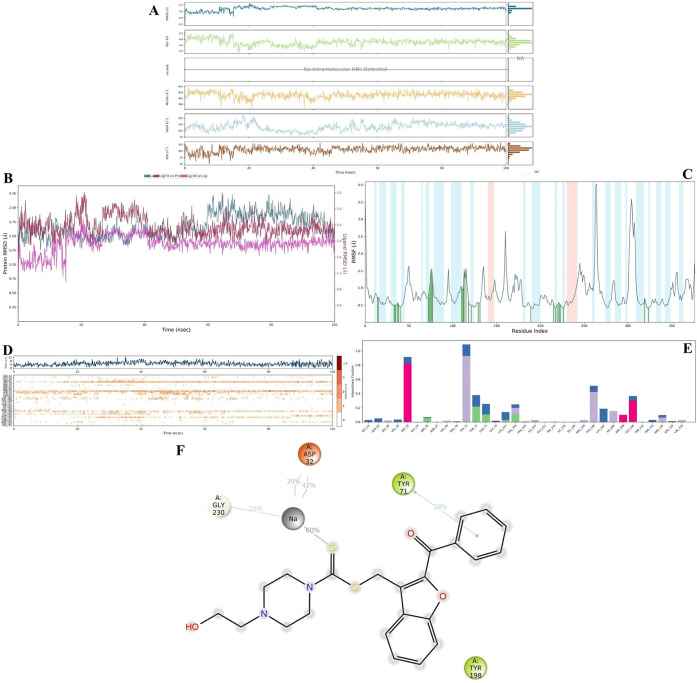
MDS result for the **4m**-BACE-1 complex. (A–C) showed the complex stability; and (D–F) showed the interaction diagrams during 100 ns simulation. (A): **4m** properties (RMSD, *R*_g_, intraHB, MolSA, SASA, and PSA; (B): RMSD values (protein; ligand fit on protein; and ligand fit on ligand); (C): RMSF diagram; (D) Interaction fraction–residue diagram; (E) total connections-residues-time plot; (F) 2D interaction pose with connection strength (cut off = 0.20) at the active region).

The MDS results (Figure 6D,F and video 2) indicated that the Asp32, Tyr71, and Gly230 residues play an important role in inhibitory activity. Notably, interactions with Asp32 and Gly230 exhibited sodium-mediated chelation after 15.90 ns. Following this time, the interactions with Asp32 remained consistent. Similar to the docking study results, the BPM moiety localized to the S2′ and S3′ subpockets and intermittently interacted with three loop regions, with Tyr71 (via π–π stacking) playing a significant role. The oxygen atom of the benzofuran group frequently formed aromatic hydrogen bonds with Tyr198, while the benzoyl group formed aromatic hydrogen bonds with Pro70, Ile126, and Tyr198. Therefore, we continue to suggest that BPM serves as an effective pharmacophore structure against β-secretase. On the other hand, the 2-hydroxyethyl group in compound **4m** occupied the S3 and S4 pockets by forming either direct or aromatic hydrogen bonds. We propose that this tail is likely responsible for the indirect inhibitory activity by enhancing the stability of the complex.

In summary, our results indicate that the main activity is attributed to 1-benzofuran-2-yl­(phenyl) methanone (BPM), while the tail plays an indirect role in modulating inhibitory activity. Additionally, we observed similar structure–activity relationships (SAR) for the ACh enzyme, as described above. These findings should be considered by pharmaceutical chemists working on the design of potential new dual inhibitors for anti-Alzheimer’s therapy.

Additionally, using the SwissADME web tool,^45^ the BBB permeability was predicted for the synthesized compounds. Compounds were not determined as positive. The blood–brain barrier (BBB) leakage is commonly observed in the early pathological stages of Alzheimer’s disease,^46^ and is now considered one of its characteristic features. Therefore, while designing compounds with BBB permeability is beneficial, it may not always be an essential prerequisite for anti-Alzheimer disease activity.

Nevertheless, the lack of BBB permeability in the current active compounds as predicted, despite their strong in vitro enzyme inhibition profiles, the structural modifications aimed at improving BBB permeability or the use of drug delivery systems to facilitate central nervous system targeting may be considered for further studies. These considerations have been briefly mentioned in the manuscript as part of future perspectives.

## 3 Conclusions

In this study, 15 novel hybrids, benzofuran-azacyclics, were designed and synthesized to investigate the theory of “anti-Alzheimer’s effects of the tubular form with the stopper.” The structures of these compounds were confirmed by HRMS, ^1^H NMR, and ^13^C NMR techniques. The final compounds (**4a–4o**) were first evaluated for their inhibitory potency on AChE, followed by testing the active molecules (**4e**, **4h**, **4m**, and **4o**) against the BACE-1 enzyme. The findings were further analyzed using in silico methods to clarify their binding modes. Subsequently, the structure–activity relationships (SARs) were proposed for future studies. The results indicated that compound **4m**, which contains *N*-(2-hydroxyethyl)­piperazine and benzofuran moieties, was the most potent. These moieties effectively occupied the substrate channel of the AChE enzyme and the catalytic cleft of the BACE-1 enzyme. Compounds **4e** (benzyl piperidine) and **4h** (2-furoyl piperazine) analogs exhibited dual inhibitory activity against both enzymes. In conclusion, the tubular form with the stopper shows great potential for Alzheimer’s disease treatment, as it blocks the entrance cavity of the AChE active pocket and enhances the stability of the inactive BACE-1 enzyme. Moreover, electrolytes (such as sodium in this case) play a pivotal role in stabilizing the **4m**-BACE-1 protein complex. These findings suggest that the synthesized compounds exhibit promising inhibitory activity across multiple enzymatic pathways. Importantly, beyond the identification of novel bioactive molecules, this study demonstrates that compounds adopting a nonclassical architecture, specifically, a “tubular form with the stopper”, may also serve as effective enzyme inhibitors, challenging the traditional design approaches. Moreover, based on the current molecular scaffold, future studies may explore structural modifications or drug delivery systems to enhance pharmacokinetic properties, including improvements in BBB permeability, thereby supporting further preclinical development.

## 4 Materials and Methods

### 4.1 Chemistry

All chemical substances were purchased from Sigma-Aldrich Chemical Co (Sigma-Aldrich Corp., St. Louis, MO, USA) and Merck Chemicals (Merck KGaA, Darmstadt, Germany). The melting points (mp) of all compounds were determined using a MP90 digital melting point apparatus (Mettler Toledo, Ohio, USA) and were uncorrected. Reactions were monitored using thin-layer chromatography (TLC) on Silica Gel 60 F254 plates (Merck KGaA, Darmstadt, Germany). Petroleum ether-ethyl acetate (3:1) was used as the mobile phase. Spectroscopic data were recorded using the following instruments: ^1^H- and ^1^C NMR spectra were obtained on a Bruker DPX-300 FT-NMR spectrometer (Bruker Bioscience, Billerica, MA, USA) in DMSO-*d*_6_, with TMS as the internal standard. High-resolution mass spectra (HRMS) were recorded on a Shimadzu 8040 LC/MS/MS system (Shimadzu, Tokyo, Japan). Detailed spectral data can be found in the Supporting Information.

#### 4.1.1 General Synthesis of (3-Methylbenzofuran-2-yl)­(phenyl)­methanone (**1**)

2′-Hydroxyacetophenone (5 mmol), 2-bromoacetophenone (5 mmol), and potassium carbonate (6 mmol) were stirred in acetonitrile under reflux for 4 h. The reaction progress was monitored using TLC. After the reaction, the solvent was completely evaporated, and the solid was washed with water, filtered, and dried. The product was crystallized from ethanol.

#### 4.1.2 General Synthesis of (3-(Bromomethyl)­benzofuran-2-yl)­(phenyl)­metanone (**2**)

(3-Methylbenzofuran-2-yl)­(phenyl)­methanone (1) (5 mmol), *N*-bromosuccinimide (NBS) (5 mmol), and benzoylperoxide (5 mmol) were stirred at reflux in CCl4 for 5 h. After the reaction, the solvent was evaporated, and the solid was washed first with water, then with cold alcohol, filtered, and dried.

#### 4.1.3 Sodium/Potassium *N*,*N*-Disubstituted Dithiocarbamate Salt Synthesis (**3a**–**o**)

An inorganic base (sodium/potassium hydroxide) (0.01 mol) was dissolved in ethanol (100 mL) with continuous stirring. After adding the secondary amine (0.01 mol), the mixture was cooled in an ice bath, and carbon disulfide (0.10 mol) was added dropwise with stirring. The reaction mixture was stirred for another hour at room temperature. Afterward, the solvent was evaporated under reduced pressure, and ether was added until precipitation occurred. The precipitate was filtered, and the product was crystallized from ethanol.

#### 4.1.4 Synthesis of (2-Benzoyl Benzofuran-3-yl)­methyl *N*,*N*-Disubstituted-1-carbodithioate Derivatives (**4a**–**o**)

[3-(Bromomethyl)­benzofuran-2-yl]­(phenyl)­methanone (**2**) (0.01 mmol) and the appropriate dithiocarbamate metal salt derivatives (**3a**–**o**) (0.01 mmol) were stirred in acetone at room temperature for 4 h. The solvent was completely evaporated, the solid was washed with water, filtered and then dried. The products were crystallized from ethanol.

##### 4.1.4.1 (2-Benzoylbenzofuran-3-yl)­methylpiperidine-1-dithiocarbamate (**4a**)

mp 142 °C. Yield % 82. ^1^H NMR (300 MHz, DMSO-*d*_6_): δ 1.54–1.59 (6H, m, piperidine-CH_2_), 3.82 (2H, s, piperidine-CH_2_), 4.22 (2H, s, piperidine-CH_2_), 5.07 (2H, s, S–CH_2_), 7.41 (H, t, *J* = 7.6 Hz, Ar–H), 7.55–7.63 (3H, m, Ar–H), 7.72 (2H, t, *J* = 7.9 Hz, Ar–H), 7.95 (H, d, *J* = 7.71 Hz, Ar–H), 8.00–8.03 (2H, m, Ar–H). ^13^C NMR (75 MHz, DMSO-*d*_6_): δ 23.97 (C, s, piperidine-CH_2_), 25.70 (C, s, piperidine-CH_2_), 26.27 (C, s, piperidine-CH_2_), 31.48 (C, s, CH_2_), 51.42 (C, s, piperidine-CH_2_), 53.13 (C, s, piperidine-CH_2_), 112.98, 122.90, 124.49, 125.12, 127.32, 129.11, 129.32, 130.03, 133.76, 137.22, 148.94, 154.14, 185.41 (C, s, C=O), 193.20 (C, s, C=S). LCMSMS (-*m*/*z*): [M + H]^+^ For C_22_H_21_NO_2_S_2_; found, 396.15.

##### 4.1.4.2 (2-Benzoylbenzofuran-3-yl)­methyl 2-Methylpiperidine-1-dithiocarbamate (**4b**)

mp 103 °C. Yield % 84. ^1^H NMR (300 MHz, DMSO-*d*_6_): δ 1.16 (3H, d, *J* = 6.86 Hz, piperidine-CH_3_), 1.47–1.68 (6H, m, piperidine-CH_2_), 2.85–3.19 (2H, m, piperidine-CH_2_), 4.25–4.41 (1H, m, piperidine-CH), 5.06 (2H, s, S–CH_2_), 7.39 (2H, t, *J* = 7.48 Hz, Ar–H), 7.77–7.71 (3H, m, Ar–H), 7.93 (H, d, *J* = 7.79 Hz, Ar–H), 7.99–8.02 (3H, m, Ar–H). LCMSMS (–*m*/*z*): [M + H]^+^ For C_23_H_23_NO_2_S_2_; found, 410.15.

##### 4.1.4.3 (2-Benzoylbenzofuran-3-yl)­methyl 3-Methylpiperidine-1-dithiocarbamate (**4c**)

mp 117 °C. Yield % 74. ^1^H NMR (300 MHz, DMSO-*d*_6_): δ 0.86–0.84 (3H, m, piperidine-CH_3_), 1.13–1.75 (6H, m, piperidine-CH_2_), 3.30–2.89 (2H, m, piperidine-CH_2_), 4.32–4.18 (H, m, piperidine-CH_2_), 5.10 (2H, s, S–CH_2_), 7.37–7.42 (H, m, Ar–H), 7.53–7.58 (H, m, Ar–H), 7.59–7.62 (2H, m, Ar–H), 7.67–7.73 (2H, m, Ar–H), 7.93 (H, d, *J* = 7.7 Hz, Ar–H), 7.80–8.03 (2H, m, Ar–H). ^13^C NMR (75 MHz, DMSO-*d*_6_): δ 19.06 (C, s, piperidine-CH_3_), 25.52 (C, s, piperidine-CH_2_), 31.53 (C, s, piperidine-CH_2_), 32.40 (C, s, piperidine-CH), 52.69 (C, s, piperidine-CH_2_), 58.78 (C, s, piperidine-CH_2_), 112.94, 122.87, 124.46, 125.10, 127.32, 129.08, 129.29, 130.03, 133.74, 137.20, 148.93, 154.12, 185.36 (C, s, C=O), 193.36 (C, s, C=S). LCMSMS (–*m*/*z*): [M + H]^+^ For C_23_H_23_NO_2_S_2_; found, 410.10.

##### 4.1.4.4 (2-Benzoylbenzofuran-3-yl)­methyl 4-Methylpiperidine-1-dithiocarbamate (**4d**)

mp 100 °C. Yield % 82. ^1^H NMR (300 MHz, DMSO-*d*_6_): δ 0.85 (3H, s, piperidine-CH_3_), 0.98–1.05 (2H, m, piperidine-CH_2_), 1.64–1.67 (4H, m, piperidine-CH_2_), 3.11–3.27 (2H, m, piperidine-CH_2_), 4.34–4.41 (H, m, piperidine-CH), 5.05 (2H, s, S–CH_2_), 7.36–7.41 (H, m, Ar–H), 7.52–7.61 (3H, m, Ar–H), 7.67–7.71 (2H, m, Ar–H), 7.99–8.02 (3H, s, Ar–H). ^13^C NMR (75 MHz, DMSO-*d*_6_): δ 21.47 (C, s, piperidine-CH_3_), 30.42 (C, s, CH_2_), 31.56 (C, s, piperidine-CH), 33.66 (C, s, piperidine-CH_2_), 34.17 (C, s, piperidine-CH_2_), 50.49 (C, s, piperidine-CH_2_), 52.34 (C, s, piperidine-CH_2_), 112.92, 122.88, 124.43, 125.15, 127.31, 129.10, 129.07, 129.27, 133.72, 137.19, 148.92, 154.12, 185.32 (C, s, C=O), 193.38 (C, s, C=S). LCMSMS (–*m*/*z*): [M + H]^+^ For C_23_H_23_NO_2_S_2_; found, 410.10.

##### 4.1.4.5 (2-Benzoylbenzofuran-3-yl)­methyl 4-Benzylpiperidine-1-dithiocarbamate (**4e**)

mp 54 °C. Yield % 81. ^1^H NMR (300 MHz, DMSO-*d*_6_): δ 1.60–1.86 (4H, m, piperidine-CH_2_), 2.08 (H, s, piperidine-CH), 2.46 (2H, d, *J* = 6.88 Hz, phenyl-CH_2_), 3.09–3.53 (4H, m, piperidine-CH_2_), 5.06 (2H, s, S–CH_2_), 7.12–7.28 (3H, m, Ar–H), 7.25 (3H, t, *J* = 7.4 Hz, Ar–H), 7.38–7.43 (H, m, Ar–H), 7.54.-7.62 (3H, m, Ar–H), 7.68–7.74 (2H, m, Ar–H), 7.94 (H, d, *J* = 7.9 Hz, Ar–H), 8.03 (H, s, CH–Ar–H). ^13^C NMR (75 MHz, DMSO-*d*_6_): δ 31.56 (2C, s, piperidine-CH_2_), 32.09 (C, s, CH_2_), 37.40 (C, s, piperidine-CH), 41.92 (C, s, phenyl-CH_2_), 50.44 (C, s, piperidine-CH_2_), 52.21 (C, s, piperidine-CH_2_), 112.97, 122.90, 125.1, 124.49, 125.11, 126.36, 127.32, 128.65, 129.10, 129.45, 130.03, 133.76, 137.21, 140.29, 148.93, 154.13, 185.38 (C, s, C=O), 193.35 (C, s, C=S). LCMSMS (–*m*/*z*): [M + H]^+^ For C_29_H_27_NO_2_S_2_; found, 486.20.

##### 4.1.4.6 (2-Benzoylbenzofuran-3-yl)­methyl Morpholine-4-dithiocarbamate (**4f**)

mp 121 °C. Yield % 85. ^1^H NMR (300 MHz, DMSO-*d*_6_): δ 3.64 (4H, s, morpholine-CH2), 3.87–4.22 (4H, m, morpholine-CH2), 5.08 (2H, s, S–CH2), 7.40–7.45 (H, m, Ar–H), 7.56–7.58 (H, m, Ar–H), 7.59–7.63 (2H, m, Ar–H), 7.69–7.75 (2H, m, Ar–H), 7.96 (H, d, *J* = 7.8 Hz, Ar–H), 8.01–8.04 (2H, m, Ar–H). ^13^C NMR (75 MHz, DMSO-*d*_6_): δ 31.20 (C, s, CH2), 50.65 (C, s, morpholine-CH_2_), 51.82 (C, s, morpholine-CH_2_), 6.03 (2C, s, morpholine-CH_2_), 112.99, 122.88, 124.53, 124.88, 127.30, 129.12, 129.35, 130.04, 133.80, 137.17, 149.02, 154.13, 185.39 (C, s, C=O), 195.12 (C, s, C=S). LCMSMS (–*m*/*z*): **[M + H]** ^**+**^ For C_21_H_19_NO_3_S_2_; found, 398.10.

##### 4.1.4.7 (2-Benzoylbenzofuran-3-yl)­methyl 4-(3-(Dimethylamino)­propyl)­piperazine-1-dithiocarbamate (**4g**)

mp 158 °C. Yield % 86. ^1^H NMR (300 MHz, DMSO-*d*_6_): δ 1.73–1.78 (2H, m, –CH_2_–CH_2_–CH_2_–*N*–), 2.33–2.43 (6H, m, *N*-(CH_3_)_2_), 2.67 (6H, s, piperazine-CH_2_), 2.92–2.97 (2H, m, –CH_2_–CH_2_–CH_2_–N–), 3.86–4.24 (4H, m, piperazine-CH_2_ ve –CH_2_-CH_2_–CH_2_–N–), 5.07 (2H, s, S–CH_2_), 7.39–7.45 (H, m, Ar–H), 7.58–7.63 (3H, m, Ar–H), 7.70–7.75 (2H, m, Ar–H), 7.95 (H, d, *J* = 8.82 Hz, Ar–H), 8.00–8.04 (2H, m, Ar–H). ^13^C NMR (75 MHz, DMSO-*d*_6_): δ 21.82 (C, s, –CH_2_–CH_2_–CH_2_–N–), 31.39 (C, s, CH_2_), 43.09 (2C, s, *N*-(CH_3_)_2_), 51.58 (C, s, –CH_2_–CH_2_–CH_2_–N–), 52.34 (C, s, –CH_2_–CH_2_–CH_2_–*N*–), 54.47 (2C, s, piperazine-CH_2_), 55.99 (2C, s, piperazine-CH_2_), 113.01, 122.88, 124.53, 124.94, 127.29, 129.14, 129.37, 130.04, 130.38, 133.82, 137.17, 148.99, 154.13, 185.40 (C, s, C=O), 194.53 (C, s, C=S). LCMSMS (–*m*/*z*): [M + H]^+^ For C_26_H_31_N_3_O_2_S_2_; found, 482.20.

##### 4.1.4.8 (2-Benzoylbenzofuran-3-yl)­methyl 4-(Furan-2-carbonyl)­piperazine-1-dithiocarbamate (**4h**)

mp 150 °C. Yield % 75. ^1^H NMR (300 MHz, DMSO-*d*_6_): δ 3.42–3.82 (4H, m, piperazine-CH_2_), 4.00–4.33 (4H, m, piperazine-CH_2_), 5.10 (2H, s, S–CH_2_), 6.64 (H, q, *J*1 = 1.77 Hz, *J*2 = 3.48 Hz, Ar–H), 7.05 (H, d, *J* = 3.45 Hz, Ar–H), 7.41–7.46 (H, m, Ar–H), 7.57–7.64 (3H, m, Ar–H),7.70–7.77 (2H, m, Ar–H), 7.86–7.87 (H, m, Ar–H), 7.98 (H, d, *J* = 7.8 Hz, Ar–H), 8.02–8.05 (2H, m, Ar–H). ^13^C NMR (75 MHz, DMSO-*d*_6_): δ 31.32 (C, s, CH_2_), 49.36 (2C, s, piperazine-CH_2_), 51.09 (2C, s, piperazine-CH_2_), 111.91, 113.01, 116.60, 122.91, 124.55, 124.88, 127.30, 129.14, 129.38, 130.06, 133.82, 137.18, 145.61, 147.03, 149.05, 154.15, 158.87, 185.41 (C, s, C=O), 195.05 (C, s, C=S). LCMSMS (–*m*/*z*): [M + H]^+^ For C_26_H_22_N_2_O_4_S_2_; found, 491.15.

##### 4.1.4.9 (2-Benzoylbenzofuran-3-yl)­methyl 4-(Pyrimidine-2-yl)­piperazine-1-dithiocarbamate (**4i**)

mp 155 °C. Yield % 70. ^1^H NMR (300 MHz, DMSO-*d*_6_): δ 3.84 (4H, s, piperazine-CH2), 3.99 (2H, s, piperazine-CH2), 4.34 (2H, s, piperazine-CH_2_), 5.10 (2H, s, S–CH_2_), 6.67 (H, t, *J* = 4.74 Hz, Ar–H), 7.39–7.45 (H, m, Ar–H), 7.56–7.63 (3H, m, Ar–H), 7.69–7.75 (2H, m, Ar–H), 7.98 (H, d, *J* = 7.02 Hz, Ar–H), 8.02–8.05 (2H, m, Ar–H), 8.37 (H, s, Ar–H), 8.39 (H, s, Ar–H). ^13^C NMR (75 MHz, DMSO-*d*_6_): δ 31.34 (C, s, CH2), 49.62 (2C, s, piperazine-CH_2_), 51.23 (2C, s, piperazine-CH_2_), 111.05, 113.00, 122.90, 124.54, 124.94, 127.30, 129.12, 129.36, 130.05, 133.80, 137.18, 149.03, 154.14, 158.44, 161.14, 185.38 (C, s, C=O), 194.88 (C, s, C=S). LCMSMS (–*m*/*z*): [M + H]^+^ For C_25_H_22_N_4_O_2_S_2_; found, 475.15.

##### 4.1.4.10 (2-Benzoylbenzofuran-3-yl)­methyl 4-Phenylpiperazine-1-dithiocarbamate (**4j**)

mp 122 °C. Yield % 79. ^1^H NMR (300 MHz, DMSO-*d*_6_): δ 3.25 (4H, s, piperazine-CH_2_), 4.01 (2H, s, piperazine-CH_2_), 4.37 (2H, s, piperazine-CH_2_), 5.01 (2H, s, S–CH_2_), 6.79 (H, t, *J* = 7.2 Hz, Ar–H), 6.91 (2H, d, *J* = 7.95 Hz, Ar–H), 7.19–7.24 (2H, m, Ar–H), 7.39–7.44 (H, m, Ar–H), 7.55–7.58 (H, m, Ar–H), 7.59–7.63 (2H, m, Ar–H), 7.72 (2H, t, *J* = 8.5 Hz, Ar–H), 7.97 (H, d, *J* = 7.8 Hz, Ar–H), 8.01–8.04 (2H, m, Ar–H). ^13^C NMR (75 MHz, DMSO-*d*_6_): δ 31.38 (C, s, CH_2_), 49.72 (2C, s, piperazine-CH_2_), 51.29 (2C, s, piperazine-CH_2_), 112.99, 115.97, 119.86, 122.91, 124.52, 124.96, 127.31, 129.12, 129.35, 130.05, 133.79, 137.19, 149.02, 150.34, 154.14, 185.39 (C, s, C=O), 194.76 (C, s, C=S). LCMSMS (–*m*/*z*): [M + H]^+^ For C_27_H_24_N_2_O_2_S_2_; found, 473.20.

##### 4.1.4.11 (2-Benzoylbenzofuran-3-yl)­methyl 4-(4-Nitrophenyl)­piperazine-1-dithiocarbamate (**4k**)

mp 181 °C. Yield % 80. ^1^H NMR (300 MHz, DMSO-*d*_6_): δ 3.68 (4H, s, piperazine-CH_2_), 4.05 (2H, s, piperazine-CH_2_), 4.37 (2H, s, piperazine-CH_2_), 5.10 (2H, s, S–CH_2_), 6.90 (2H, d, *J* = 9.5 Hz, Ar–H), 7.40–7.45 (H, m, Ar–H), 7.56–7.58 (H, m, Ar–H), 7.59–7.64 (2H, m, Ar–H), 7.69–7.76 (2H, m, Ar–H), 7.98 (H, d, *J* = 6.03 Hz, Ar–H), 8.02–8.09 (4H, m, Ar–H). ^13^C NMR (75 MHz, DMSO-*d*_6_): δ 31.25 (C, s, CH_2_), 48.70 (2C, s, piperazine-CH_2_), 50.61 (C, s, piperazine-CH_2_), 112.20, 113.01, 122.92, 124.54, 124.94, 126.26, 127.29, 129.14, 129.37, 130.06, 130.50, 133.82, 137.17, 137.20, 149.05, 154.08, 154.14, 185.40 (C, s, C=O), 194.85 (C, s, C=S). LCMSMS (–*m*/*z*): [M + H]^+^ For C_27_H_23_N_3_O_4_S_2_; found, 518.15.

##### 4.1.4.12 (2-Benzoylbenzofuran-3-yl)­methyl 4-(4-Methoxyphenyl)­piperazine-1-dithiocarbamate (**4l**)

mp 108 °C. Yield % 88. ^1^H NMR (300 MHz, DMSO-*d*_6_): δ 3.10 (2H, s, piperazine-CH_2_), 3.45 (2H, s, piperazine-CH_2_), 3.67 (3H, s, O–CH3), 4.01 (2H, s, piperazine-CH_2_), 4.38 (2H, s, piperazine-CH_2_), 5.10 (2H, s, S–CH_2_), 6.81–6.84 (2H, m, Ar–H), 6.91 (2H, d, *J* = 9.18 Hz, Ar–H), 7.43 (H, t, *J* = 7.60 Hz, Ar–H), 7.56–7.59 (H, m, Ar–H), 7.61–7.64 (2H, m, Ar–H), 7.70–7.77 (2H, m, Ar–H), 7.98 (H, d, *J* = 7.86 Hz, Ar–H), 8.01–8.05 (2H, m, Ar–H). ^13^C NMR (75 MHz, DMSO-*d*_6_): δ 31.39 (C. s, CH_2_), 49.86 (4C, s, piperazine-CH_2_), 55.63 (C, s, O–CH_3_), 113.01, 114.77, 118.42, 122.91, 124.54, 124.94, 127.31, 129.14, 129.37, 130.05, 133.81, 137.20, 144.72, 149.02, 154.15, 185.42 (C, s, C=O), 194.73 (C, s, C=S). LCMSMS (–*m*/*z*): [M + H]^+^ For C_28_H_26_N_2_O_3_S_2_; found, 503.20.

##### 4.1.4.13 (2-Benzoylbenzofuran-3-yl)­methyl 4-(2-Hydroxyethyl)­piperazine-1-dithiocarbamate (**4m**)

mp 127 °C. Yield % 86. ^1^H NMR (300 MHz, DMSO-*d*_6_): δ 2.47 (2H, t, *J* = 5.99 Hz, HO–CH_2_–CH_2_–), 2.54 (4H, s, piperazine-CH_2_), 3.52 (2H, t, *J* = 5.94 Hz, HO–CH_2_–CH_2_−), 3.86 (2H, s, piperazine-CH_2_), 4.24 (2H, s, piperazine-CH_2_), 4.54 (H, s, –OH), 5.06 (2H, s, S–CH_2_), 7.36–7.42 (H, m, Ar–H), 7.52–7.61 (3H, m, Ar–H), 7.671–7.71 (2H, m, Ar–H), 7.93 (H, d, *J* = 7.8 Hz, Ar–H), 8.00–8.02 (2H, m, Ar–H). ^13^C NMR (75 MHz, DMSO-*d*_6_): δ 31.46 (C, s, CH_2_), 49.89 (2C, s, piperazine-CH_2_), 51.34 (C, s, piperazine-CH_2_), 52.78 (C, s, piperazine-CH_2_), 58.62 (C, s, hydroxyethyl- CH_2_), 59.75 (C, s, hydroxyethyl-CH_2_), 112.93, 122.85, 124.46, 124.96, 127.29, 129.08, 130.01, 133.73, 137.18, 148.98, 154.12, 185.30 (C, s, C=O), 194.64 (C, s, C=S). LCMSMS (-*m*/*z*): [M + H]^+^ For C_23_H_24_N_2_O_3_S_2_; found, 441.10.

##### 4.1.4.14 (2-Benzoylbenzofuran-3-yl)­methyl 4-(4-Chlorophenyl)­piperazine-1-dithiocarbamate (**4n**)

mp 93 °C. Yield % 77. ^1^H NMR (300 MHz, DMSO-*d*_6_): δ 3.49 (4H, s, piperazine-CH_2_), 4.02 (2H, s, piperazine-CH_2_), 4.38 (2H, s, piperazine-CH_2_), 5.10 (2H, s, S–CH_2_), 6.92 (2H, d, *J* = 9.10 Hz, Ar–H), 7.24 (2H, d, *J* = 8.9 Hz, Ar–H), 7.35–7.45 (2H, m, Ar–H), 7.57–7.64 (2H, m, Ar–H), 7.69–7.76 (2H, m, Ar–H), 7.87 (H, d, *J* = 8.01 Hz, Ar–H), 7.97–8.05 (2H, m, Ar–H). ^13^C NMR (75 MHz, DMSO-*d*_6_): δ 31.38 (C, s, CH_2_), 47.68 (4C, s, piperazine-CH_2_), 112.99, 117.21, 117.86, 122.90, 123.13, 124.53, 124.91, 127.32, 129.13, 129.18, 129.35, 130.03, 133.79, 137.21, 149.04, 149.23, 154.16, 185.42 (C, s, C=O), 194.84 (C, s, C=S). LCMSMS (-*m*/*z*): [M + H]^+^ For C_27_H_23_ClN_2_O_2_S_2_; found, 507.15.

##### 4.1.4.15 (2-Benzoylbenzofuran-3-yl)­methyl 4-(4-Fluorophenyl)­piperazine-1-dithiocarbamate (**4o**)

mp 109 °C. Yield % 73. ^1^H NMR (300 MHz, DMSO-*d*_6_): δ 3.16 (4H, s, piperazin-CH_2_), 4.00 (2H, s, piperazine-CH_2_), 4.37 (2H, s, piperazine-CH_2_), 5.10 (2H, s, S–CH_2_), 6.89–6.94 (2H, m, Ar–H), 7.04 (2H, t, *J* = 8.85 Hz, Ar–H), 7.40 (H, t, *J* = 7.25 Hz, Ar–H), 7.54–7.62 (3H, m, Ar–H), 7.70 (2H, t, *J* = 7.32 Hz, Ar–H), 7.97 (H, d, *J* = 7.8 Hz, Ar–H), 8.02–8.05 (2H, m, Ar–H). ^13^C NMR (75 MHz, DMSO-*d*_6_): δ 31.45 (C, s, CH_2_), 48.92 (2C, s, piperazine-CH_2_), 49.81 (C, s, piperazine-CH_2_), 51.31 (C, s, piperazine-CH_2_), 112.94, 115.70 ve 115.99, 117.80, 117.90, 122.88, 124.48, 124.97, 127.32, 129.08, 129.31, 130.03, 133.74, 137.19, 147.34, 147.36, 149.02, 154.15, 155.19 ve 158.32, 185.31 (C, s, C=O), 194.89 (C, s, C=S). LCMSMS (-*m*/*z*): [M + H]^+^ For C_27_H_23_FN_2_O_2_S_2_; found, 491.15.

### 4.2 Cholinesterase Activity

According to the modified Ellman approach outlined in our earlier works^47–49^ the AChE and BChE inhibitory activities of the obtained compounds were evaluated. The enzymes utilized in the experiment were human AChE (CAS no. 9000-81-1) and human BChE (CAS no. 9001-08-5).

### 4.3 β-Secretase Activity

The experimental procedure was based on the “Human β-Secretase (BACE-1) Inhibitor Screening Assay” kit (Human β-Secretase (BACE1) Inhibitor Screening Kit (Fluorometric)-Catalog no: K720-100) protocol based on the fluorometric method as performed previously.^50^

### 4.4 Kinetic Studies of AChE Enzyme Inhibition

The compound **4m**, which was found to be the most effective derivative in the series, was included in the enzyme kinetics study to assign the type of inhibition. For this purpose, this compound was prepared at different concentrations (IC_50_, 2xIC_50_ and IC_50/2_). Moreover, a substrate (ATC) was used at various concentrations (600, 300, 150, 75, 37.5, and 18.75 μM). The enzyme kinetics assay was carried out as in our previous.^51–53^ Lineweaver–Burk plots were formed using Microsoft Office Excel 2013. The *K*_i_ values of the compound were easily calculated from the second plot with a common intercept on the *x*-axis (corresponding to –*K*_i_).

### 4.5 Molecular Docking and Molecular Dynamics Simulation (MDS) Studies

The in silico docking procedure was applied to understand potential interactions, which point out to us what’s the relation between ligands and acetylcholinesterase and β-secretase enzymes (PDBID: 4EY7 and 2ZJM, respectively). Herewith, it helps us to understand the behavior of how active compounds act in the active region of the enzyme. Considering in vitro enzyme tests, compounds **4e**, **4h**, **4m**, and **4o** were docked into AChE active pocket and BACE-1 binding cavity using structure-based in silico docking procedure.^4,54–57^ After docking studies, to understand environmental effects regarding time on the stability and behavior of ligand–protein complex, the most active compound on both enzymes, **4m**, was used as a model for its analogs. The MDS method for 100 ns simulation time was applied the same as performed previously by our team.^4,54–57^

## Supplementary Material



## Notes

Includes the characterization data (^1^H NMR, ^13^C NMR, and MS) for the synthesized compounds; this additional material provides further confirmation and details relevant to the experimental section of the manuscript (PDF)

## References

[1] Canudas-Romo V. (2010). Three measures
of longevity: Time trends and record values. Demography.

[2] Hugar L. A., Wulff-Burchfield E. M., Winzelberg G. S., Jacobs B. L., Davies B. J. (2021). Incorporating
palliative care principles to improve patient care and quality of
life in urologic oncology. Nat. Rev. Urol.

[3] Drozdowska D., Maliszewski D., Wróbel A., Ratkiewicz A., Sienkiewicz M. (2023). New Benzamides
as Multi-Targeted Compounds: A Study
on Synthesis, AChE and BACE1 Inhibitory Activity and Molecular Docking. Int. J. Mol. Sci..

[4] Saglik B. N., Levent S., Osmaniye D., Evren A. E., Karaduman A. B., Ozkay Y., Kaplancikli Z. A. (2022). Design, Synthesis, and In Vitro and
In Silico Approaches of Novel Indanone Derivatives as Multifunctional
Anti-Alzheimer Agents. ACS Omega.

[5] Iraji A., Khoshneviszadeh M., Firuzi O., Khoshneviszadeh M., Edraki N. (2020). Novel small molecule
therapeutic agents for Alzheimer
disease: Focusing on BACE1 and multi-target directed ligands. Bioorg. Chem..

[6] Demirayak S., Yurttas L., Cagri Karaburun A., Gundogdu-Karaburun N., Kayagil I. (2016). Synthesis and Antiproliferative Activity
of 2-arylidene
6-(2-aryl-2-oxoethoxy)­Benzofuran-3-one Derivatives. Lett. Drug Des. Discovery.

[7] Gao Y., Ma C., Feng X., Liu Y., Haimiti X. (2020). BF12, a novel benzofuran,
exhibits antitumor activity by inhibiting microtubules and the PI3K/Akt/mTOR
signaling pathway in human cervical cancer cells. Chem. Biodiversity.

[8] Eldehna W. M., Nocentini A., Elsayed Z. M., Al-Warhi T., Aljaeed N., Alotaibi O. J., Al-Sanea M. M., Abdel-Aziz H. A., Supuran C. T. (2020). Benzofuran-Based Carboxylic Acids as Carbonic Anhydrase
Inhibitors and Antiproliferative Agents against Breast Cancer. ACS Med. Chem. Lett..

[9] Adalat B., Rahim F., Taha M., Hayat S., Iqbal N., Ali Z., Shah S. A. A., Wadood A., Rehman A. U., Khan K. M. (2022). Synthesis
of Benzofuran–based Schiff bases as anti-diabetic compounds
and their molecular docking studies. J. Mol.
Struct..

[10] Abd
El-Karim S. S., Mahmoud A. H., Al-Mokaddem A. K., Ibrahim N. E., Alkahtani H. M., Zen A. A., Anwar M. M. (2023). Development
of a New Benzofuran–Pyrazole–Pyridine-Based Molecule
for the Management of Osteoarthritis. Molecules.

[11] Shejol V. M., Singh N. (2022). Synthesis and Potential
Anti-Anxiety Activity of Chalcone Benzofuran
Derivatives. NeuroQuantology.

[12] Strelow D. N., Magalhães L. S., Paim M. P., Krüger L. D., Neto J. S. S., Brüning C. A., Bortolatto C. F. (2023). Depressive-like
behavior and cognitive impairment induced by acute administration
of dexamethasone: Pharmacological effects of 2-phenyl-3-(phenylselanyl)
benzofuran in female Swiss mice. Prog. Neuro-Psychopharmacol.
Biol. Psychiatry.

[13] Dawbaa S., Evren A. E., Saglik B. N., Gundogdu-Karaburun N., Karaburun A. C. (2022). Biological activity evaluation of
novel monoamine oxidase
inhibitory compounds targeting Parkinson disease. Future Med. Chem..

[14] Karaburun A. C. (2019). Synthesis
and Anticandidal Activities of Some Aryl (5-Chloro-Benzofuran- 2-yl)
Ketoximes. Lett. Drug Des. Discovery.

[15] Karaburun A. C. ¸. (2019). Synthesis and Antifungal Activity
of New Nitrobenzofuran Derivatives. J. Osaka
Inst. Sci. Technol.

[16] Ceyhun İ., Karaca Ş., Osmaniye D., Sağlık B. N., Levent S., Özkay Y., Kaplancıklı Z. A. (2022). Design
and synthesis of novel chalcone derivatives and evaluation of their
inhibitory activities against acetylcholinesterase. Archiv der Pharmazie.

[17] Yilmaz A., Koca M., Boga M., Kurt A., Ozturk T. (2023). Synthesis
of Novel Oxime and Benzofuran Chemical Frameworks Possessing Potent
Anticholinesterase Activity: A SAR Study Related to Alzheimer Disease. ChemistrySelect.

[18] Kaur R., Narang S. S., Singh P., Goyal B. (2023). Structural and molecular
insights into tacrine-benzofuran hybrid induced inhibition of amyloid-β
peptide aggregation and BACE1 activity. J. Biomol.
Struct. Dyn..

[19] Goyal D., Kaur A., Goyal B. (2018). Benzofuran
and Indole: Promising
Scaffolds for Drug Development in Alzheimer’s Disease. ChemMedChem.

[20] Paudel P., Seong S. H., Zhou Y., Ha M. T., Min B. S., Jung H. A., Choi J. S. (2019). Arylbenzofurans from the root bark
of Morus alba as triple inhibitors of cholinesterase, β-site
amyloid precursor protein cleaving enzyme 1, and glycogen synthase
kinase-3β: Relevance to Alzheimer’s disease. ACS omega.

[21] Yun Y., Miao Y., Sun X., Sun J., Wang X. (2021). Synthesis
and biological evaluation of 2-arylbenzofuran derivatives as potential
anti-Alzheimer’s disease agents. J. Enzyme
Inhib. Med. Chem..

[22] Cui X., Huang Z., Deng S., Zhang Y., Li G., Wang L., Deng Y., Wu C. (2024). Benzofuran Derivatives
from Cortex Mori Radicis and Their Cholinesterase-Inhibitory Activity. Molecules.

[23] Evren A. E., Eksellİ B., YurttaŞ L., Temel H. E., AkalİN
ÇİFtÇİ G. (2025). Design and synthesis of new benzothiazole-piperazine
derivatives and in vitro and in silico investigation of their anticancer
activity. J. Mol. Struct..

[24] Evren A. E., Yurttaş L., Eksellı B., Akalın-Cıftcı G. (2019). Novel Tri-substituted
Thiazoles Bearing Piperazine Ring: Synthesis and Evaluation of their
Anticancer Activity. Lett. Drug Des. Discovery.

[25] Asha R. N., Sankarganesh M., Bhuvanesh N., Nayagam B. R. D. (2022). Synthesis, structural,
spectral, antidiabetic, DNA interactions and molecular docking investigations
of a piperidine derivative. J. Mol. Struct..

[26] Bindu B., Vijayalakshmi S., Manikandan A. (2020). Synthesis and discovery of triazolo-pyridazine-6-yl-substituted
piperazines as effective anti-diabetic drugs; evaluated over dipeptidyl
peptidase-4 inhibition mechanism and insulinotropic activities. Eur. J. Med. Chem..

[27] Jain A., Chaudhary J., Khaira H., Chopra B., Dhingra A. (2021). Piperazine:
a promising scaffold with analgesic and anti-inflammatory potential. Drug Research.

[28] Xiong J., Jin J., Gao L., Hao C., Liu X., Liu B.-F., Chen Y., Zhang G. (2020). Piperidine propionamide as a scaffold
for potent sigma-1 receptor antagonists and mu opioid receptor agonists
for treating neuropathic pain. Eur. J. Med.
Chem..

[29] Partyka A., Chlon-Rzepa G., Wasik A., Jastrzebska-Wiesek M., Bucki A., Kolaczkowski M., Satala G., Bojarski A. J., Wesolowska A. (2015). Antidepressant-
and anxiolytic-like activity of 7-phenylpiperazinylalkyl-1,3-dimethyl-purine-2,6-dione
derivatives with diversified 5-HT(1)­A receptor functional profile. Bioorg. Med. Chem..

[30] Partyka A., Jastrzębska-Więsek M., Canale V., Zajdel P., Wesołowska A. (2018). Anxiolytic-like
activity of PZ-1433, a novel arylsulfonamide
derivative of aryloxy (propyl) piperidine, in rodents. Medicina Internacia Revuo.

[31] Costanzo P., Cariati L., Desiderio D., Sgammato R., Lamberti A., Arcone R., Salerno R., Nardi M., Masullo M., Oliverio M. (2016). Design, synthesis,
and evaluation of donepezil-like
compounds as AChE and BACE-1 inhibitors. ACS
Med. Chem. Lett..

[32] Gabr M. T., Abdel-Raziq M. S. (2018). Design
and synthesis of donepezil analogues as dual
AChE and BACE-1 inhibitors. Bioorg. Chem..

[33] Qin P., Ran Y., Xie F., Liu Y., Wei C., Luan X., Wu J. (2023). Design, synthesis,
and biological evaluation of novel N-Benzyl piperidine
derivatives as potent HDAC/AChE inhibitors for Alzheimer’s
disease. Bioorg. Med. Chem..

[34] Kiran P. V. R., Waiker D. K., Verma A., Saraf P., Bhardwaj B., Kumar H., Singh A., Kumar P., Singh N., Srikrishna S. (2023). Design and development of benzyl piperazine
linked 5-phenyl-1, 2, 4-triazole-3-thione conjugates as potential
agents to combat Alzheimer’s disease. Bioorg. Chem..

[35] Waiker D. K., Verma A., Akhilesh, Singh N., Singh N., Roy A., Dilnashin H., Tiwari V., Trigun S. K., Singh S. P. (2023). Design, Synthesis, and Biological Evaluation of Piperazine and N-Benzylpiperidine
Hybrids of 5-Phenyl-1, 3, 4-oxadiazol-2-thiol as Potential Multitargeted
Ligands for Alzheimer’s Disease Therapy. ACS Chem. Neurosci..

[36] Sharma P., Tripathi A., Tripathi P. N., Prajapati S. K., Seth A., Tripathi M. K., Srivastava P., Tiwari V., Krishnamurthy S., Shrivastava S. K. (2019). Design
and development of multitarget-directed N-Benzylpiperidine analogs
as potential candidates for the treatment of Alzheimer’s disease. Eur. J. Med. Chem..

[37] Bajad N. G., Singh R. B., Gajendra T., Gutti G., Kumar A., Krishnamurthy S., Singh S. K. (2024). Development of multi-targetable chalcone
derivatives bearing N-aryl piperazine moiety for the treatment of
Alzheimer’s disease. Bioorg. Chem..

[38] Mezeiova E., Chalupova K., Nepovimova E., Gorecki L., Prchal L., Malinak D., Kuca K., Soukup O., Korabecny J. (2019). Donepezil
derivatives targeting amyloid-β cascade in Alzheimer’s
disease. Curr. Alzheimer Res..

[39] Pathak C., Kabra U. D. (2024). A comprehensive
review of multi-target directed ligands
in the treatment of Alzheimer’s disease. Bioorg. Chem..

[40] Mathew B., Oh J. M., Baty R. S., Batiha G. E.-S., Parambi D. G. T., Gambacorta N., Nicolotti O., Kim H. (2021). Piperazine-substituted
chalcones: A new class of MAO-B, AChE, and BACE-1 inhibitors for the
treatment of neurological disorders. Environ.
Sci. Pollut. Res..

[41] Yurttaş L., Evren A. E., AlChaib H., Temel H. E., Akalin
Çiftçi G. (2024). Synthesis, molecular docking, and molecular dynamic
simulation studies of new 1,3,4-thiadiazole derivatives as potential
apoptosis inducers in A549 lung cancer cell line. J. Biomol. Struct. Dyn..

[42] Yucel N. T., Asfour A. A. R., Evren A. E., Yazici C., Kandemir U., Ozkay U. D., Can O. D., Yurttas L. (2024). Design and synthesis
of novel dithiazole carboxylic acid Derivatives: In vivo and in silico
investigation of their Anti-Inflammatory and analgesic effects. Bioorg. Chem..

[43] Evren A. E., Karaduman A. B., Saglik B. N., Ozkay Y., Yurttas L. (2023). Investigation
of Novel Quinoline-Thiazole Derivatives as Antimicrobial Agents: In
Vitro and In Silico Approaches. ACS Omega.

[44] Dawbaa S., Nuha D., Evren A. E., Cankiliç M. Y., Yurttaş L., Turan G. (2023). New oxadiazole/triazole
derivatives
with antimicrobial and antioxidant properties. J. Mol. Struct..

[45] Daina A., Zoete V. (2016). A BOILED-Egg To Predict
Gastrointestinal Absorption and Brain Penetration
of Small Molecules. ChemMedChem.

[46] Nehra G., Bauer B., Hartz A. M. S. (2022). Blood-brain barrier leakage in Alzheimer’s
disease: From discovery to clinical relevance. Pharmacol. Ther..

[47] Ellman G. L., Courtney K. D., Andres V., Feather-Stone R. M. (1961). A new and
rapid colorimetric determination of acetylcholinesterase activity. Biochem. Pharmacol..

[48] Osmaniye D., Evren A. E., Sağlık B.
N., Levent S., Ozkay Y., Kaplancıklı Z. A. (2022). Design, synthesis,
biological activity, molecular docking, and molecular dynamics of
novel benzimidazole derivatives as potential AChE/MAO-B dual inhibitors. Arch. Pharm. (Weinheim, Ger.).

[49] Durmaz Ş., Evren A. E., Sağlık B.
N., Yurttaş L., Tay N. F. (2022). Synthesis, anticholinesterase activity, molecular docking,
and molecular dynamic simulation studies of 1,3,4-oxadiazole derivatives. Arch. Pharm. (Weinheim, Ger.).

[50] Tok F., Sağlık B. N., Özkay Y., Kaplancıklı Z. A., Koçyiğit-Kaymakçıoğlu B. (2022). Design, synthesis,
biological activity evaluation and in silico studies of new nicotinohydrazide
derivatives as multi-targeted inhibitors for Alzheimer’s disease. J. Mol. Struct..

[51] Saglik B. N., Ilgin S., Ozkay Y. (2016). Synthesis
of new donepezil analogues
and investigation of their effects on cholinesterase enzymes. Eur. J. Med. Chem..

[52] Demir
Ozkay U., Can O. D., Saglik B. N., Acar
Cevik U., Levent S., Ozkay Y., Ilgin S., Atli O. (2016). Design, synthesis, and AChE inhibitory activity of new benzothiazole-piperazines. Bioorg. Med. Chem. Lett..

[53] Tok F., Koçyigit-Kaymakçioglu B., Saglik B. N., Levent S., Özkay Y., Kaplancikli Z. A. (2019). Synthesis and biological evaluation
of new pyrazolone Schiff bases as monoamine oxidase and cholinesterase
inhibitors. Bioorg. Chem..

[54] Al-Sharabi A. A., Evren A. E., Saglik B. N., Yurttas L. (2023). Synthesis,
characterization,
molecular docking and molecular dynamics simulations of novel 2,5-disubstituted-1,3,4-thiadiazole
derivatives as potential cholinesterase/monoamine oxidase dual inhibitors
for Alzheimer’s disease. J. Biomol. Struct.
Dyn..

[55] Naji S. A., Sağlik B. N., Agamennone M., Evren A. E., Gundogdu-Karaburun N., Karaburun A. C. ¸. (2023). Design and Evaluation of Synthesized
Pyrrole Derivatives as Dual COX-1 and COX-2 Inhibitors Using FB-QSAR
Approach. ACS Omega.

[56] Yurttaş L., Evren A. E., Kubilay A., Aksoy M. O., Temel H. E., Akalın Çiftçi G. (2023). Synthesis of Some New 1,3,4-Oxadiazole
Derivatives and Evaluation of Their Anticancer Activity. ACS Omega.

[57] Evren A. E., Nuha D., Dawbaa S., Saglik B. N., Yurttas L. (2022). Synthesis
of novel thiazolyl hydrazone derivatives as potent dual monoamine
oxidase-aromatase inhibitors. Eur. J. Med. Chem..

