# Supplementary material for: Novel Benzofuran-3-yl-methyl and Aliphatic Azacyclics: Design, Synthesis, and In Vitro and In Silico anti-Alzheimer Disease Activity Studies
Source: ACS Omega. 2025 Jul 22;10(30):32829–43. doi: 10.1021/acsomega.5c01432 (PMC12332554; doi:10.1021/acsomega.5c01432)
Supplement: Supplementary file 1 [file ao5c01432_si_001.pdf]

Novel Benzofuran-3-yl-methyl and aliphatic azacyclics: Design, synthesis, and in vitro and in silico anti-Alzheimer disease activity studies

Büşra GEBEŞ-ALPEREN[1], Asaf Evrim EVREN[1,*], Begüm Nurpelin SAĞLIK ÖZKAN[1], Ahmet Cagri KARABURUN[1], Nalan GUNDOGDU-KARABURUN[1,*]

[1] Anadolu University, Faculty of Pharmacy, Department of Pharmaceutical Chemistry, 26470, Eskişehir, Turkey.

✉ Nalan GUNDOGDU-KARABURUN (0000-0002-8808-8697),
E-mail: ngundogd@anadolu.edu.tr

***This file includes analytical spectra of the final molecules.**

1.1.1.1. (2-benzoylbenzofuran-3-yl)methyl piperidin-1-dithiocarbamate (4a)

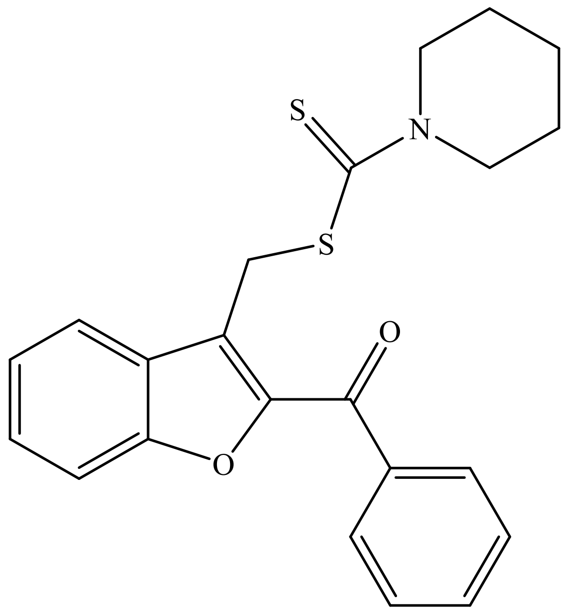

Figure S1. *Compound **4a***

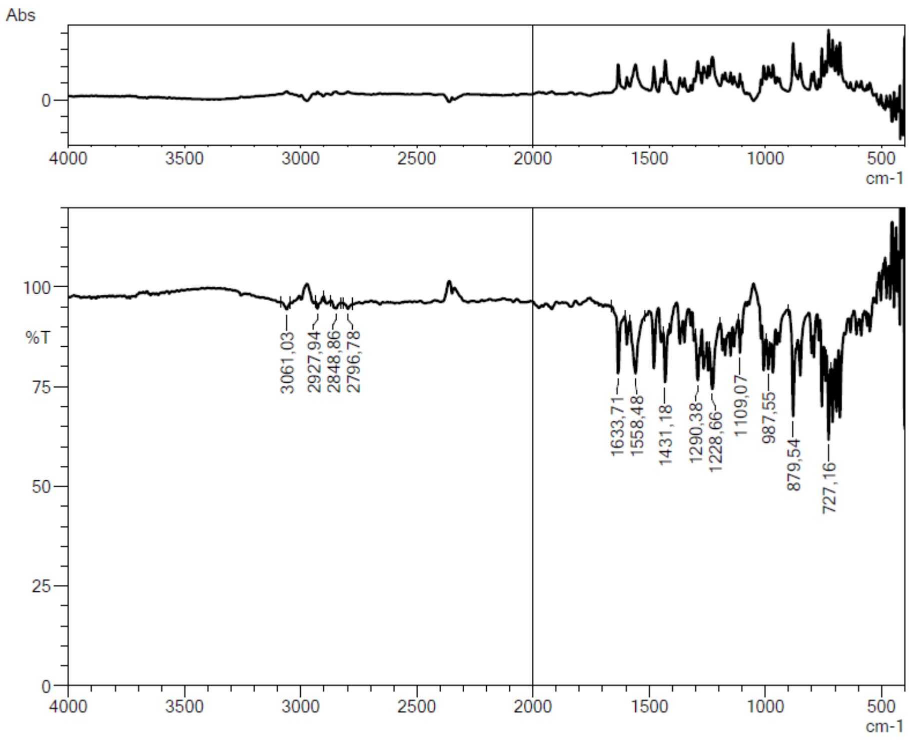

Figure S2. *Compound **4a**-IR spectrum*

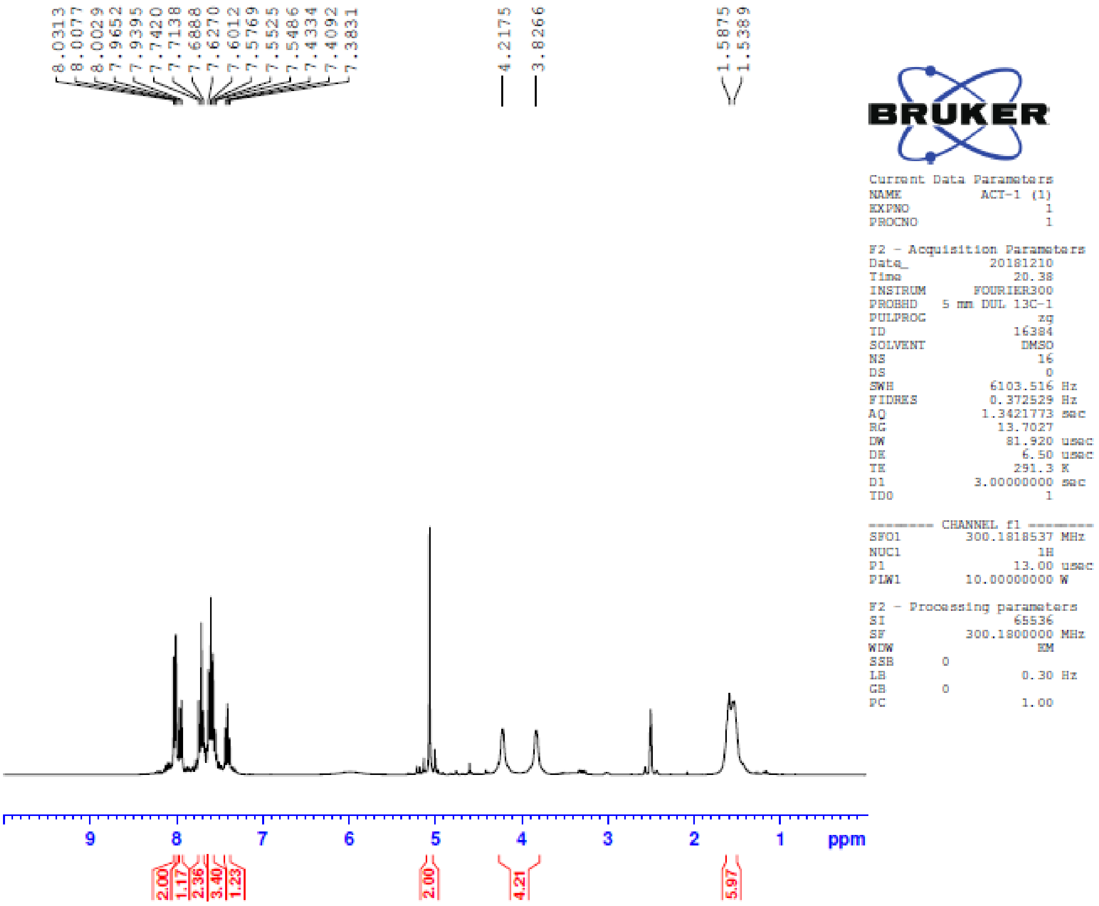

Figure S3. *Compound **4a**-^{1}H-NMR spectrum*

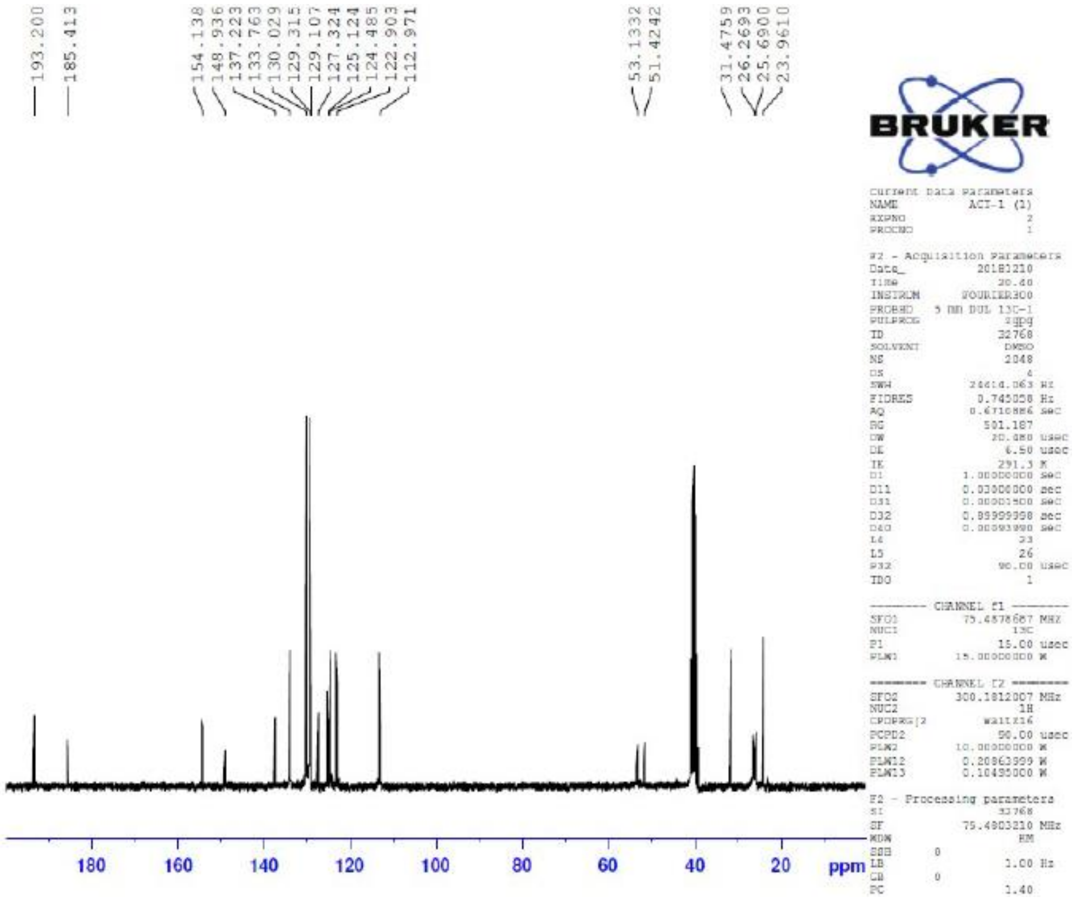

Figure S4. *Compound* ***4a***-^{13}*C-NMR spectrum*

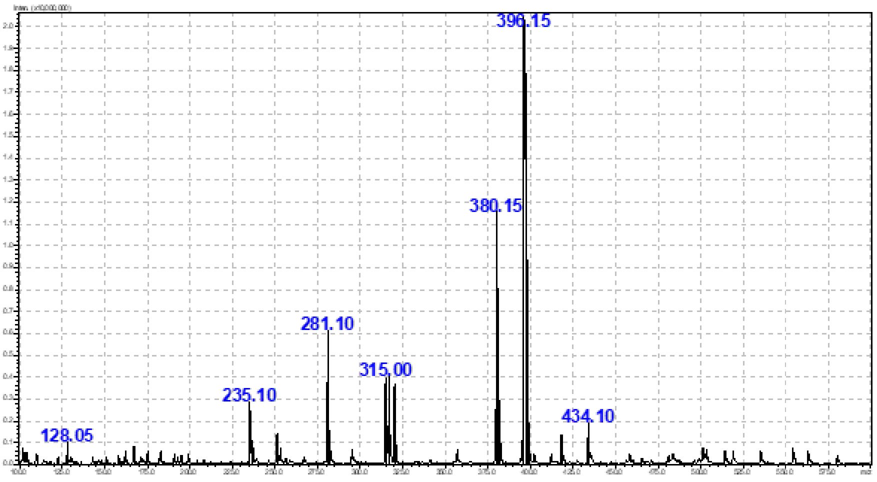

Figure S5. *Compound* ***4a****-LC-MSMS spectrum*

1.1.1.2. (2-benzoylbenzofuran-3-yl)methyl 2-methylpiperidin-1-dithiocarbamate (4b)

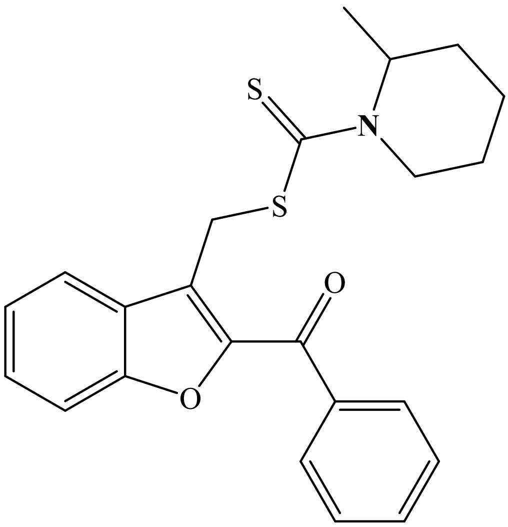

Figure S6. *Compound **4b***

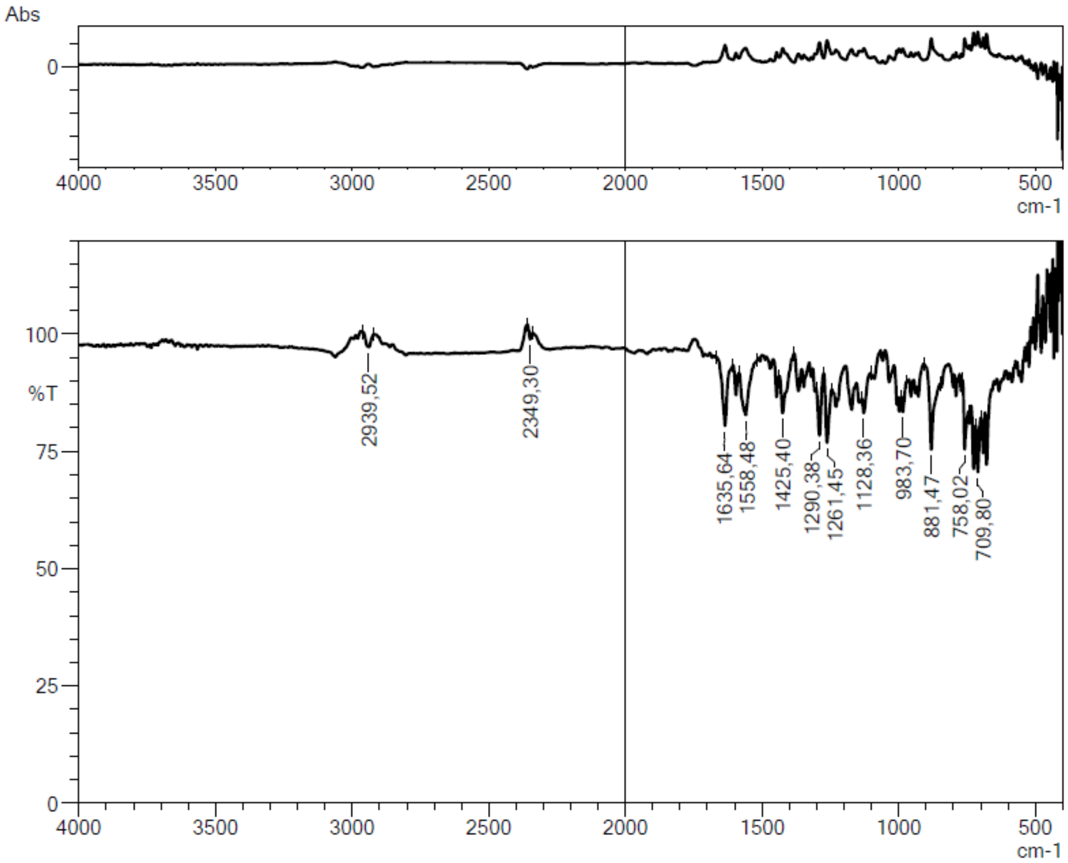

Figure S7. *Compound **4b**-IR spectrum*

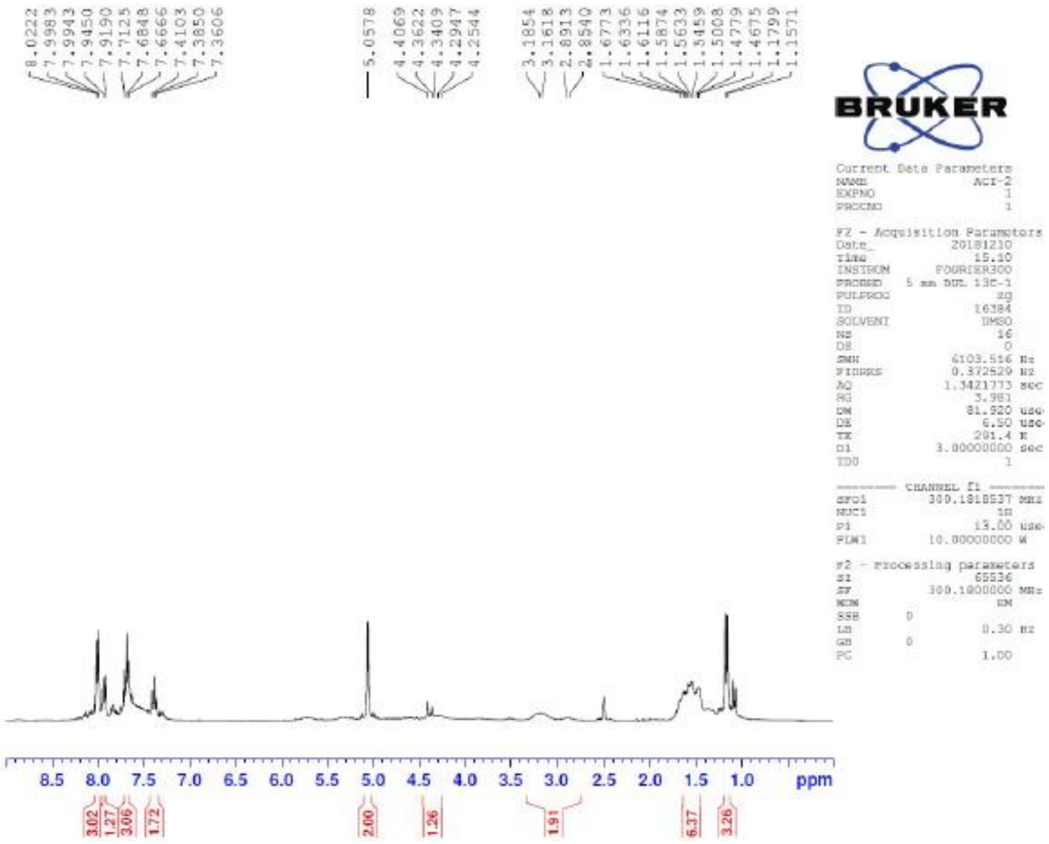

Figure S8. *Compound **4b**-^{1}H-NMR spectrum*

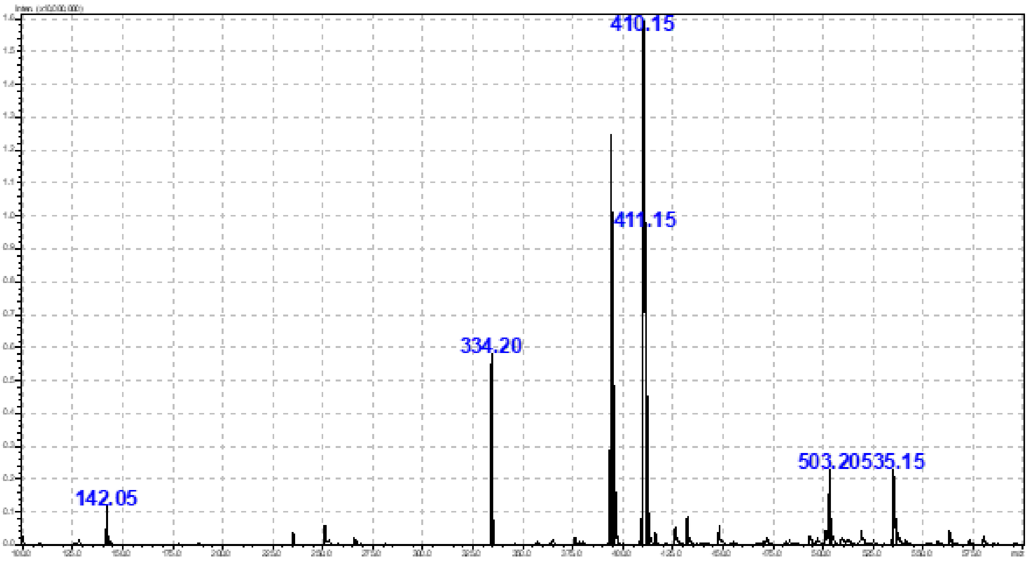

Figure S9. *Compound **4b**-LC-MSMS spectrum*

1.1.1.3. (2-benzoylbenzofuran-3-yl)methyl 3-methylpiperidin-1-dithiocarbamate (4c)

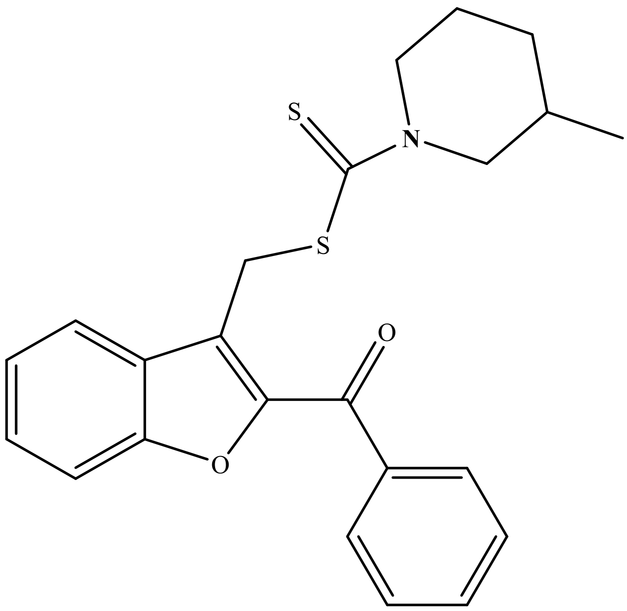

Figure S10. *Compound* ***4c***

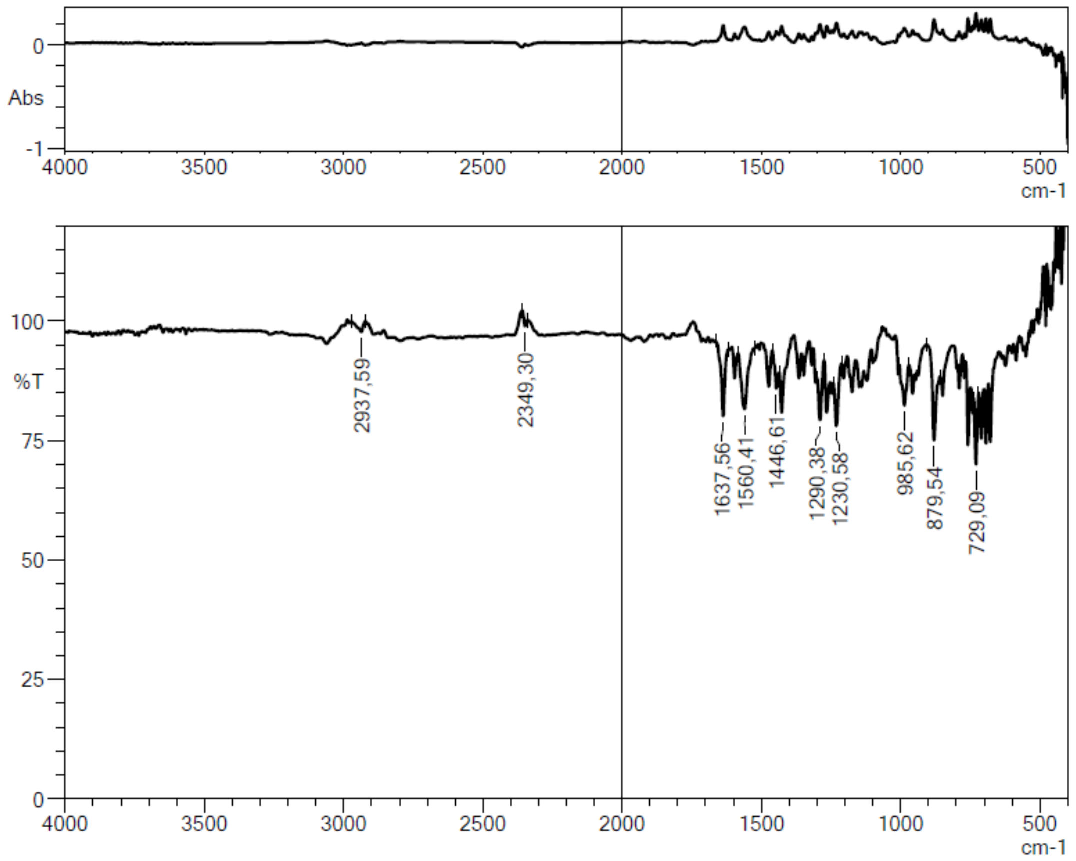

Figure S11. *Compound* ***4c****-IR spectrum*

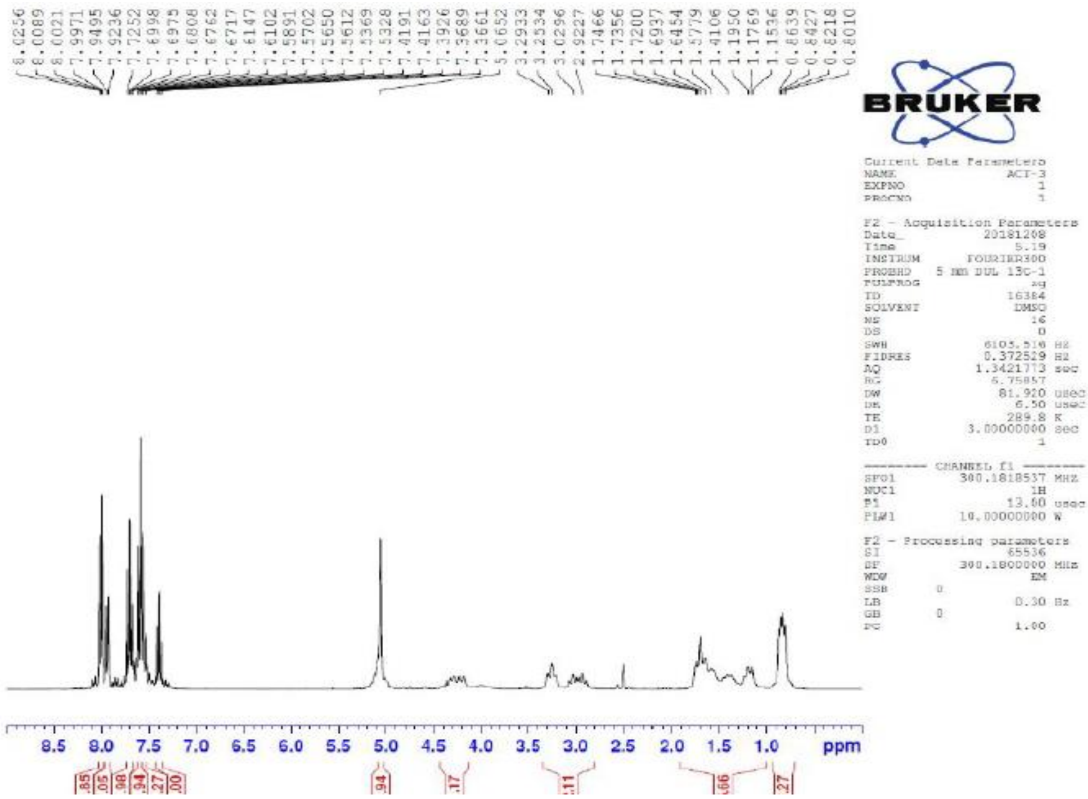

Figure S12. *Compound **4c**-^{1}H-NMR spectrum*

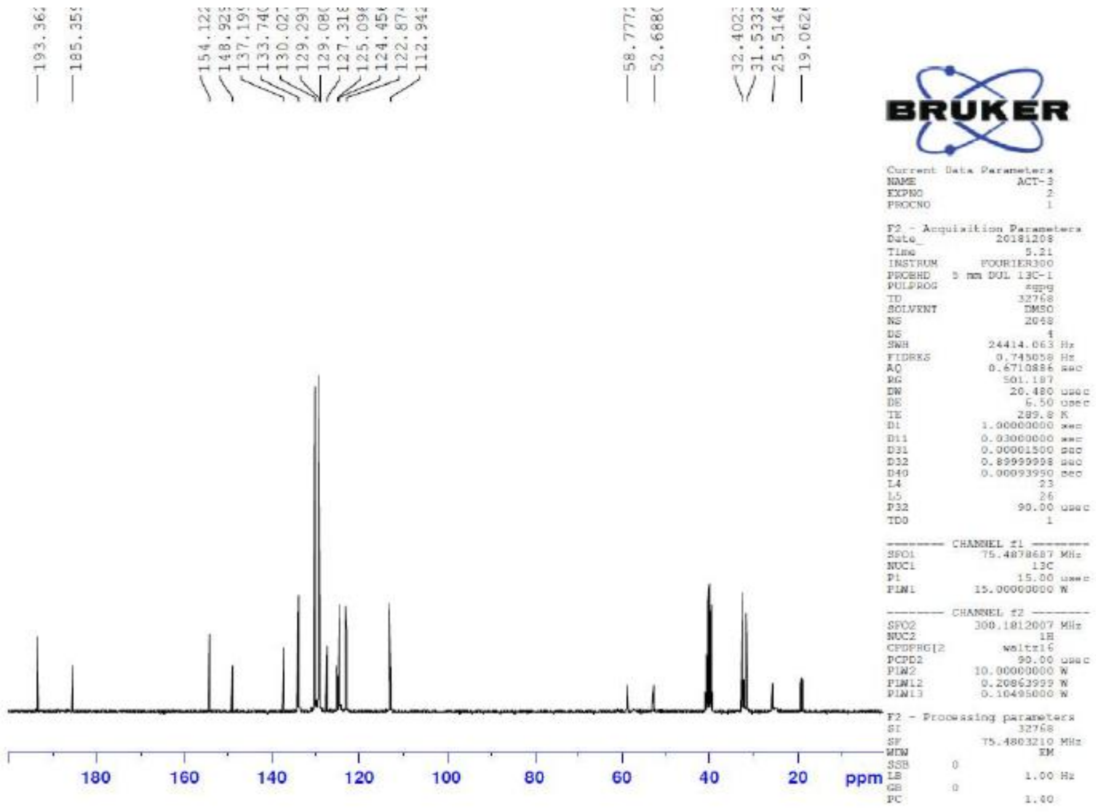

Figure S13. *Compound **4c**-^{13}C-NMR spectrum*

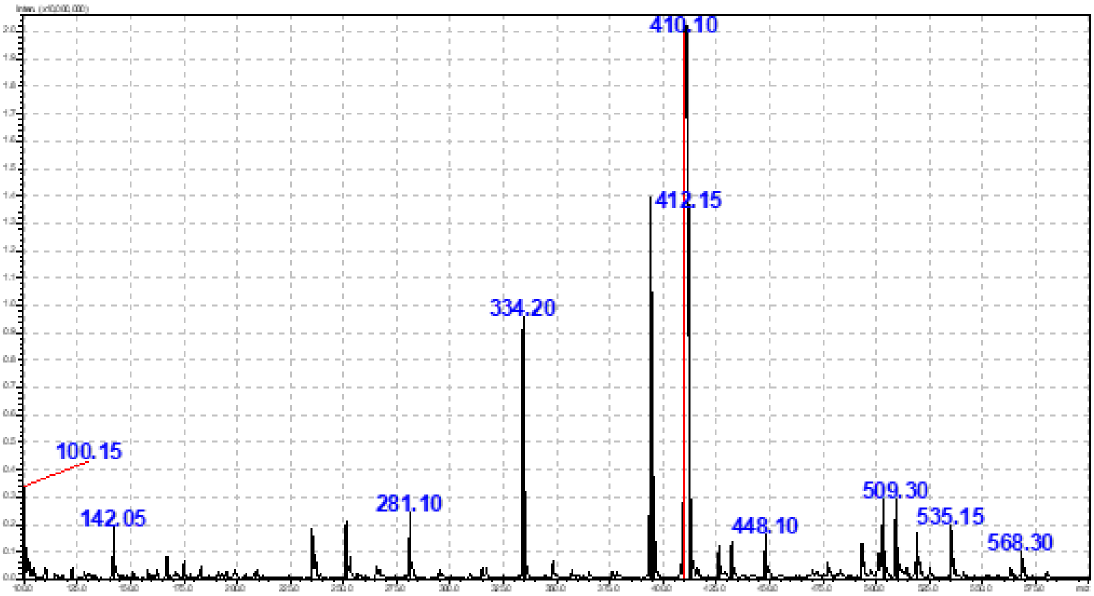

Figure S14. *Compound **4c**-LC-MSMS spectrum*

1.1.1.4. (2-benzoylbenzofuran-3-yl)methyl 4-methylpiperidin-1-dithiocarbamate (4d)

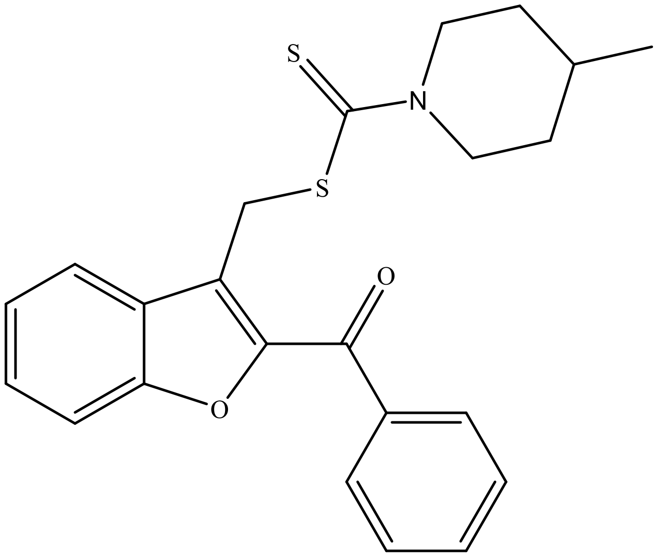

Figure S15. *Compound **4d***

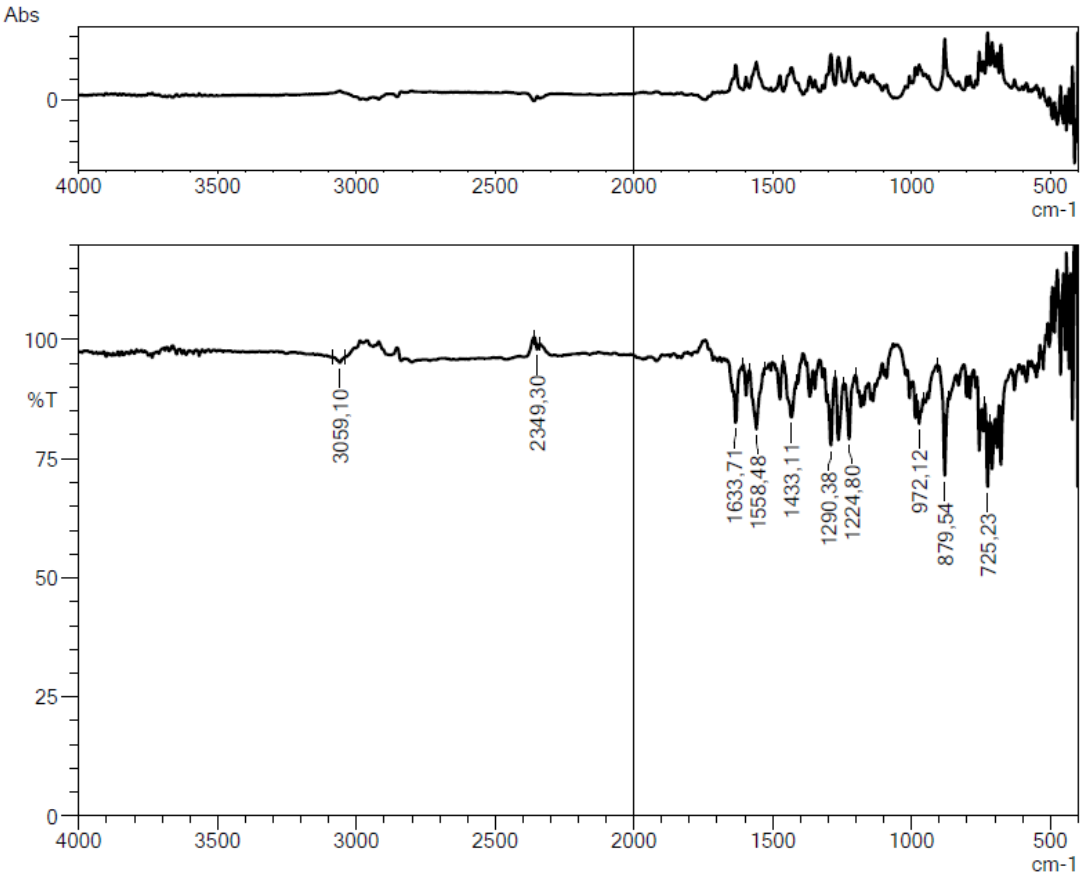

Figure S16. *Compound **4d**-IR spectrum*

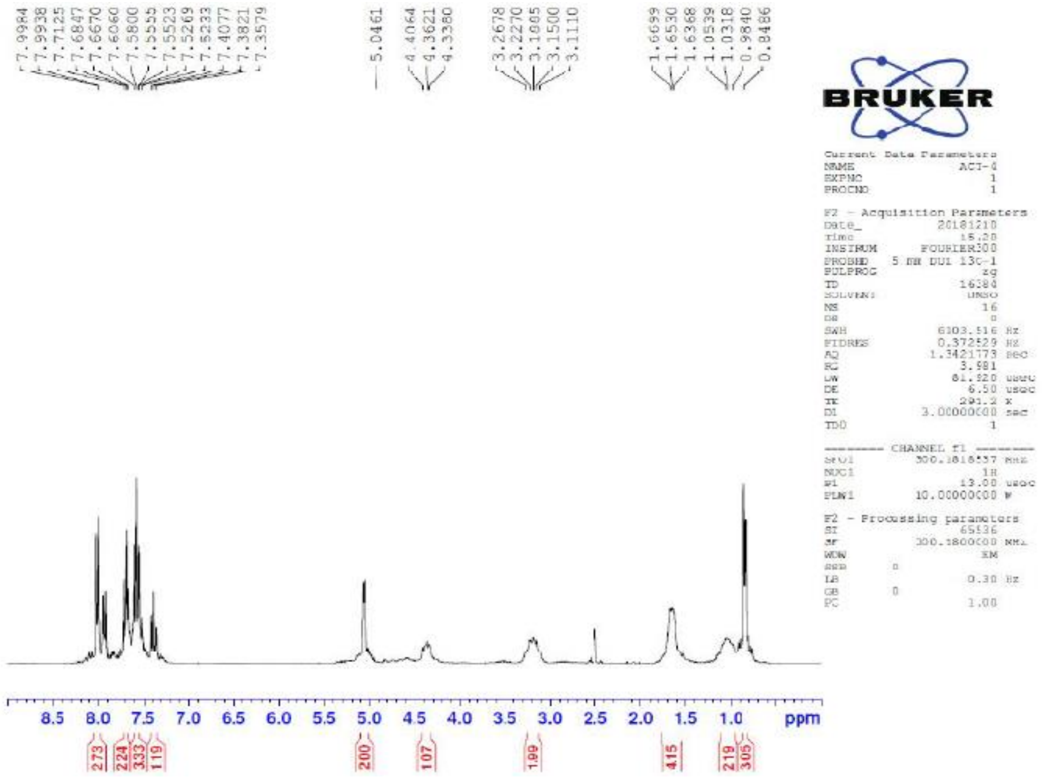

Figure S17. *Compound **4d**-^{1}H-NMR spectrum*

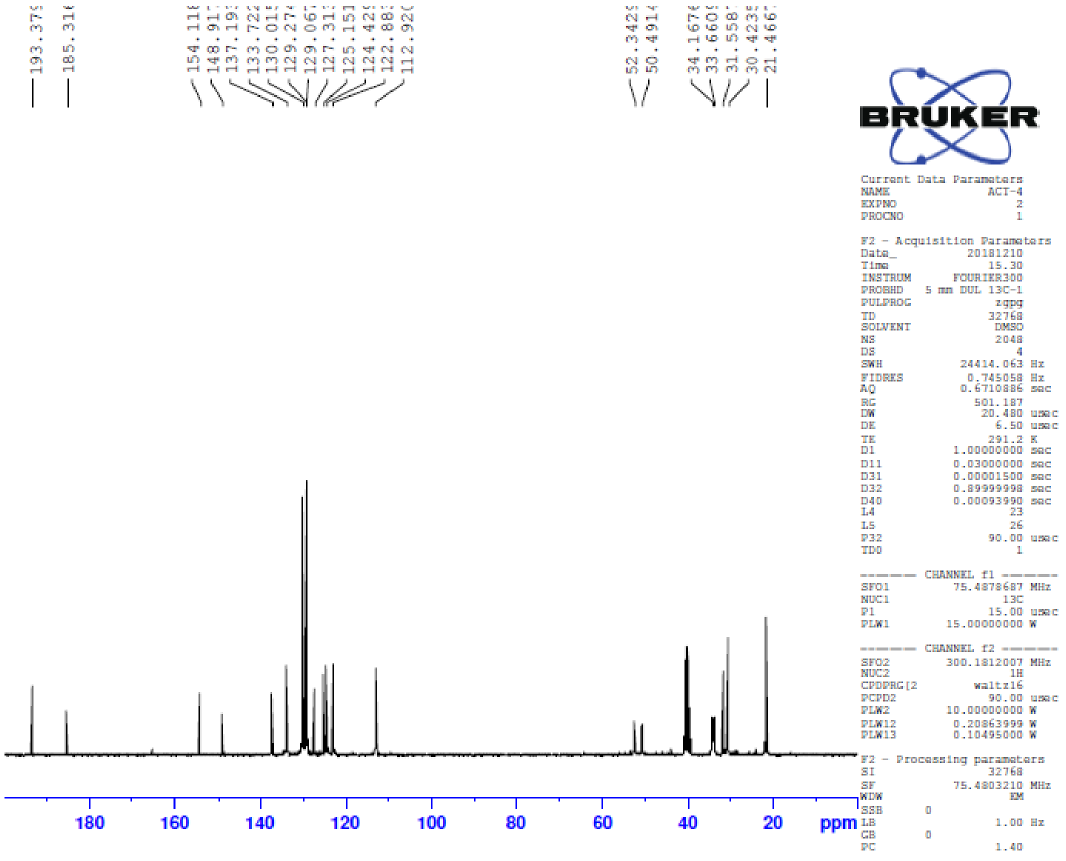

Figure S18. *Compound **4d**-^{13}C-NMR spectrum*

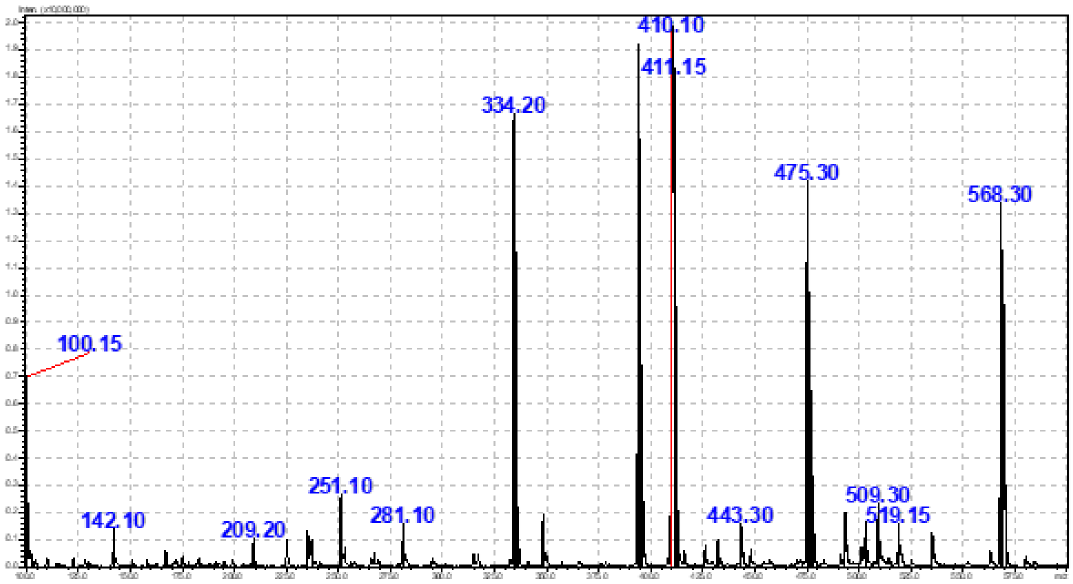

Figure S19. *Compound **4d**-LC-MSMS spectrum*

1.1.1.5. (2-benzoylbenzofuran-3-yl)methyl 4-benzylpiperidin-1-dithiocarbamate (4e)

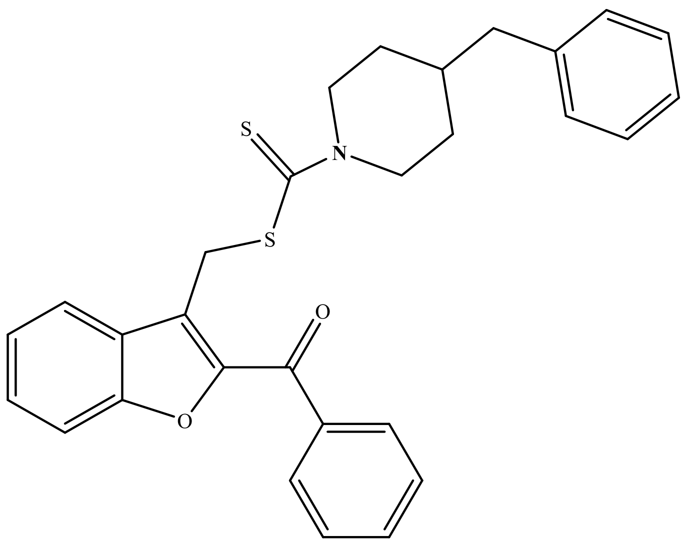

Figure S20. *Compound **4e***

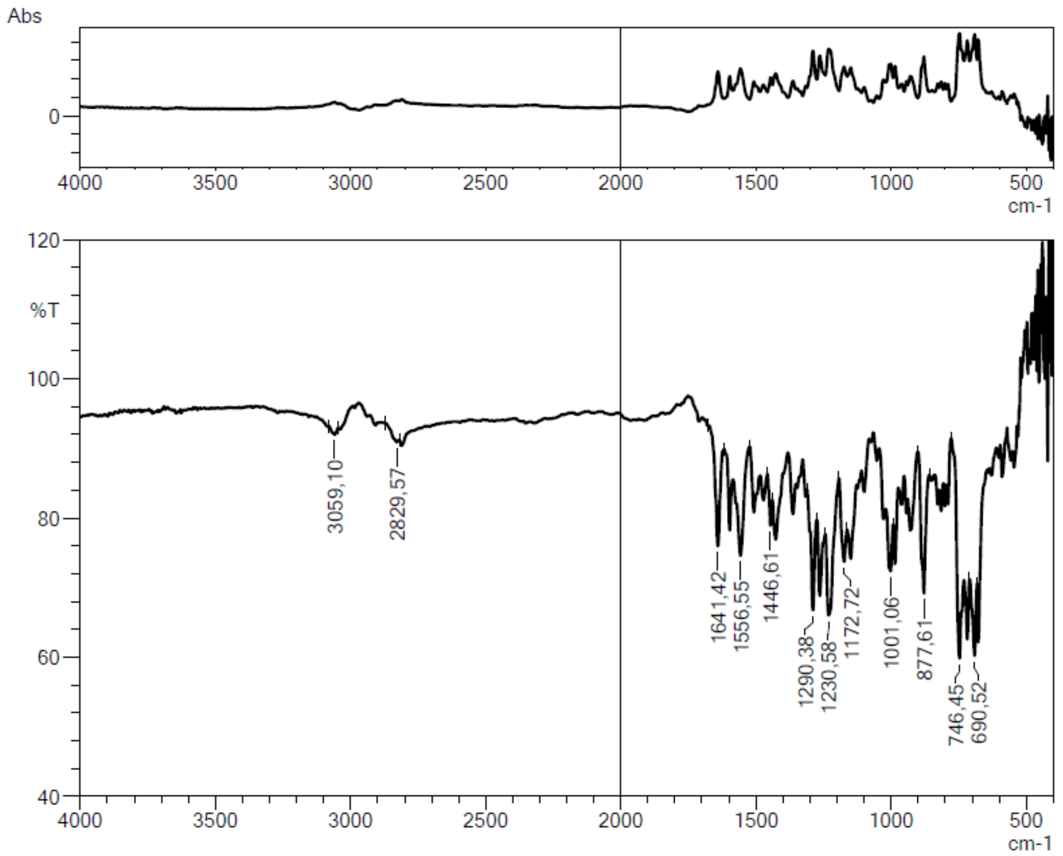

Figure S21. *Compound **4e**-IR spectrum*

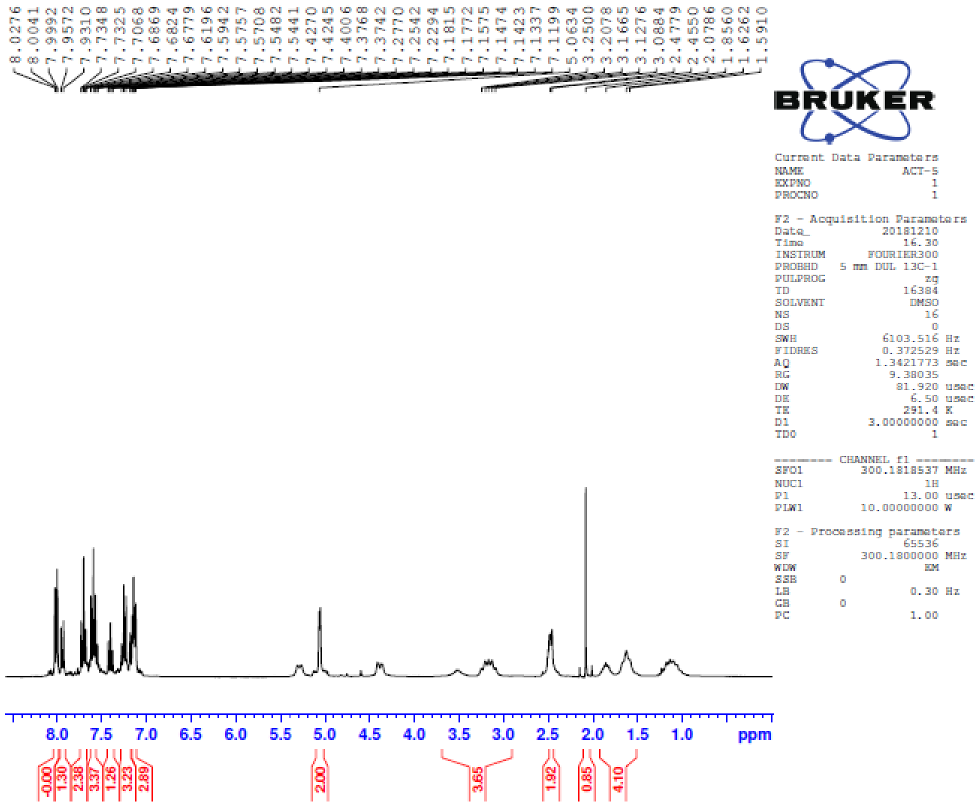

Figure S22. *Compound **4e**-^{1}H-NMR spectrum*

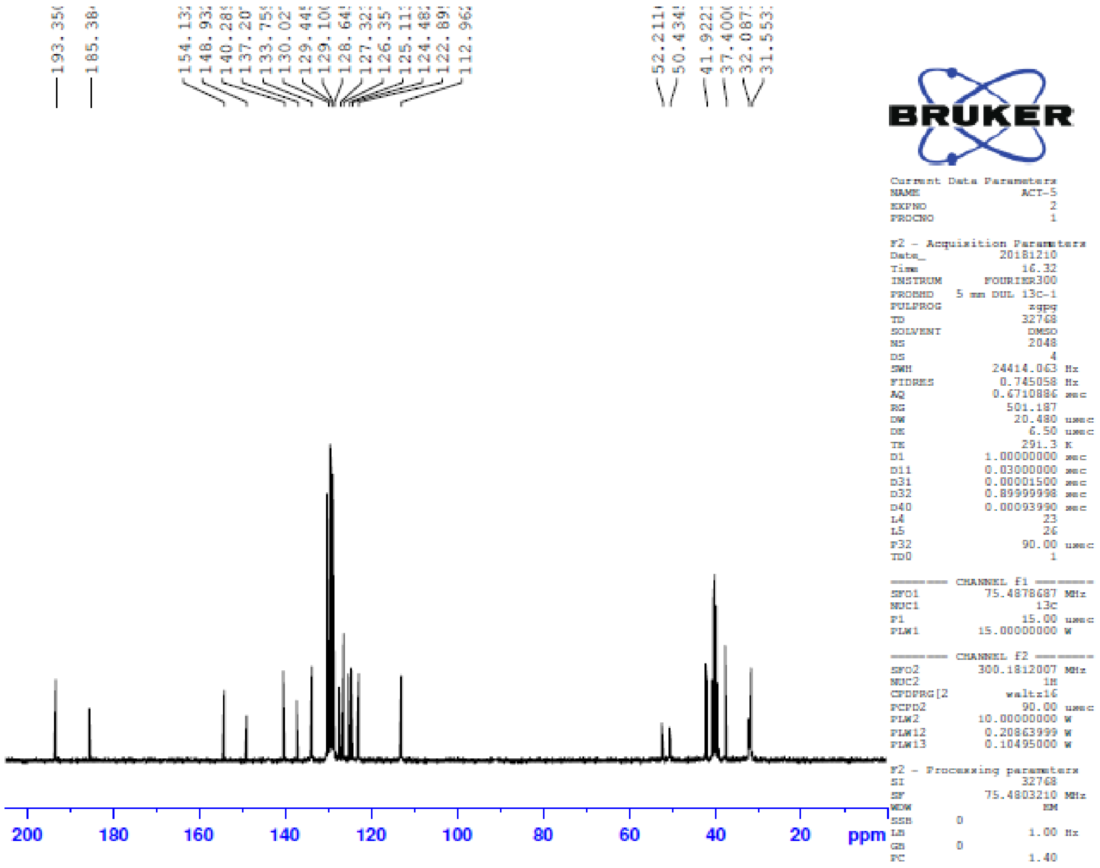

Figure S23. *Compound* ***4e****-^{13}C-NMR spectrum*

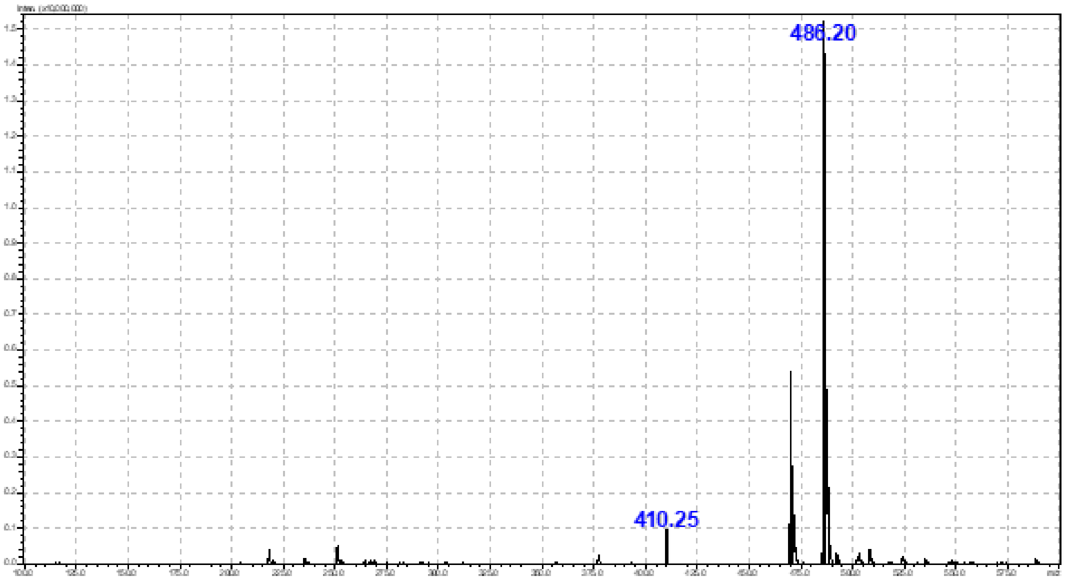

Figure S24. *Compound* ***4e****-LC-MSMS spectrum*

1.1.1.6. (2-benzoylbenzofuran-3-yl)methyl morpholin-4-dithiocarbamate (4f)

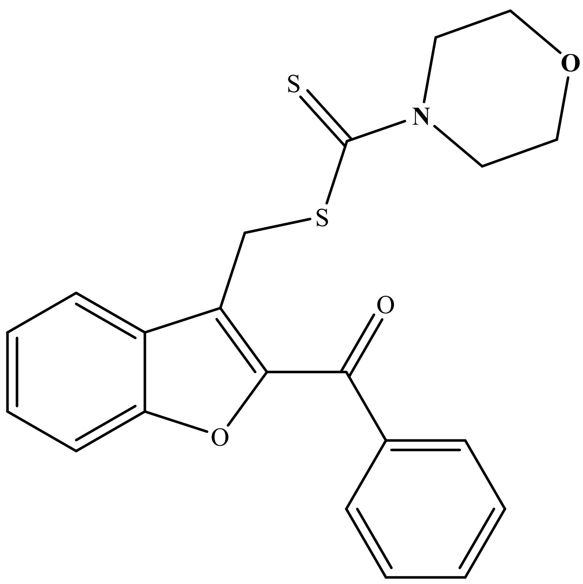

Figure S25. *Compound **4f***

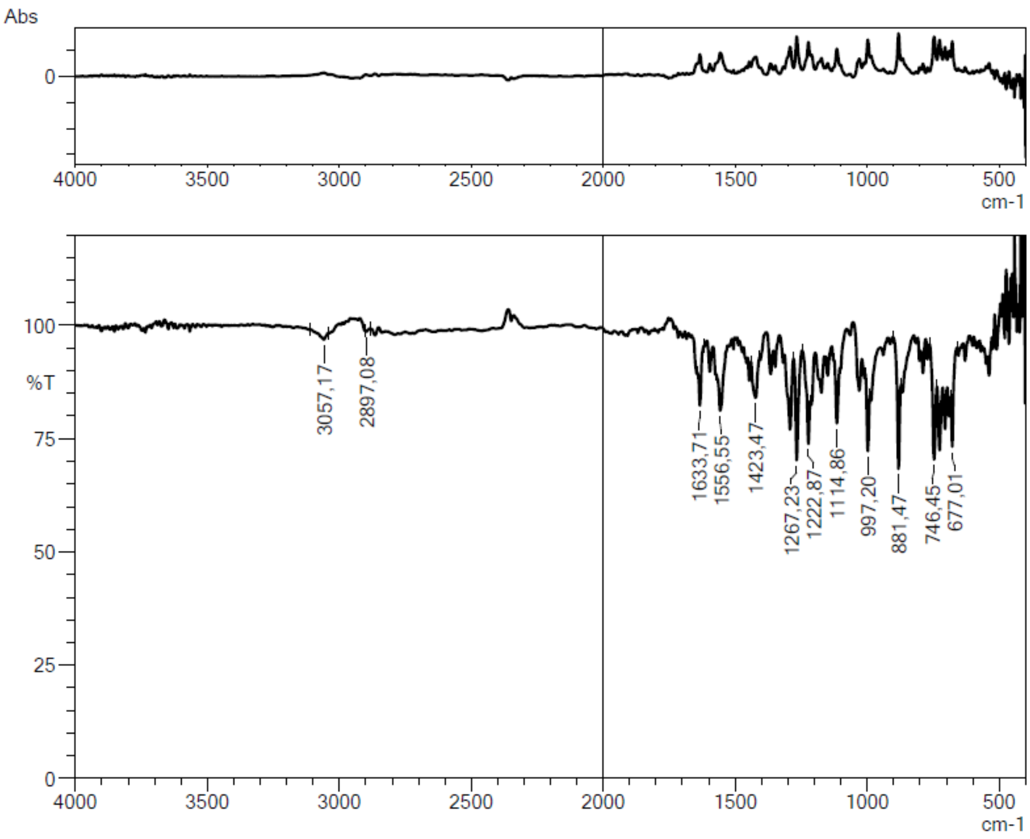

Figure S26. *Compound **4f**-IR spectrum*

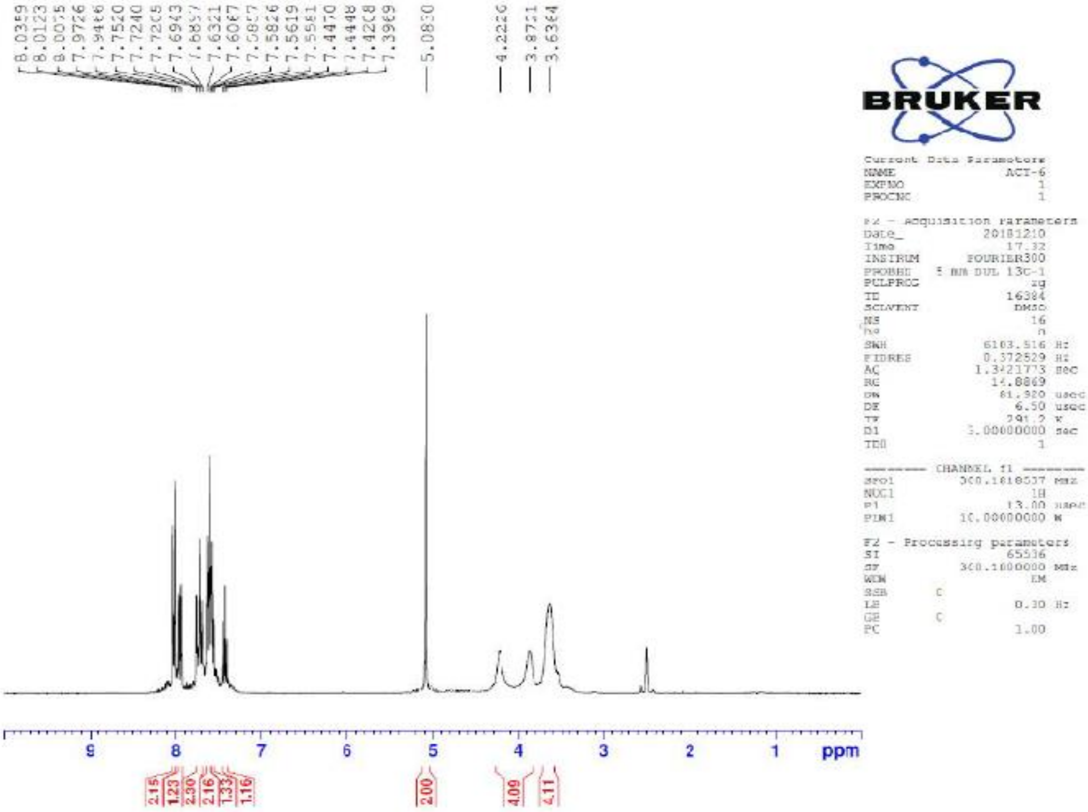

Figure S27. *Compound **4f**-^{1}H-NMR spectrum*

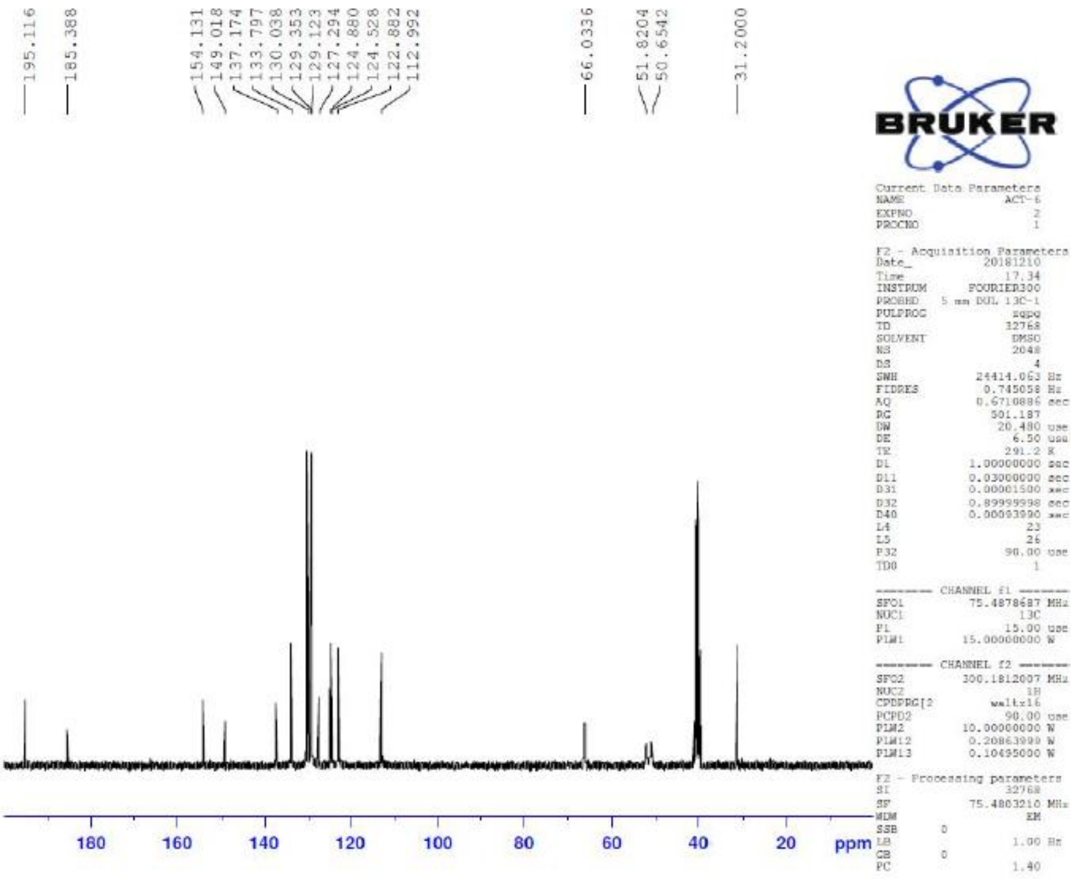

Figure S28. *Compound* ***4f***-^{13}*C-NMR spectrum*

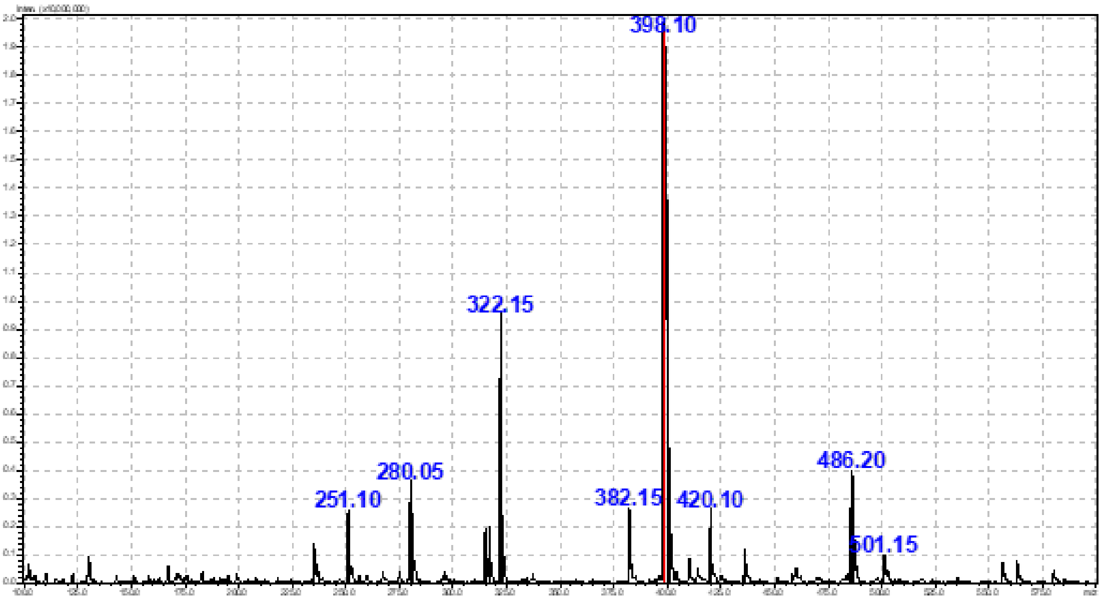

Figure S29. *Compound* ***4f***-*LC-MSMS spectrum*

1.1.1.7. (2-benzoylbenzofuran-3-yl)methyl 4-(3-(dimethylamino)propyl)piperazin-1-dithiocarbamate (4g)

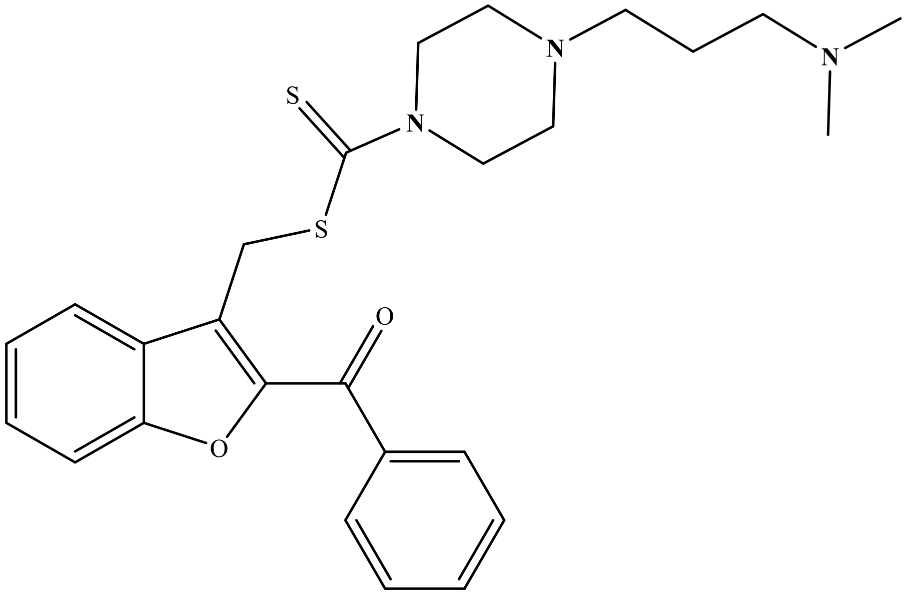

Figure S30. *Compound* ***4g***

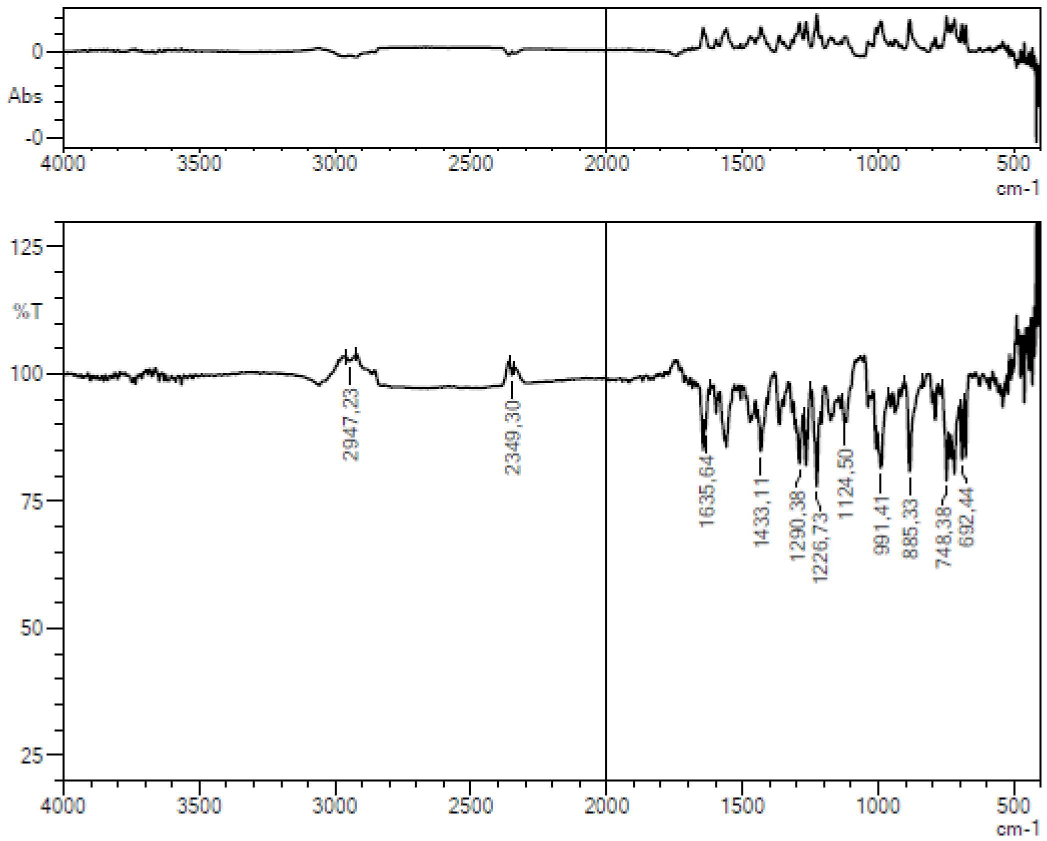

Figure S31. *Compound **4g**-IR spectrum*

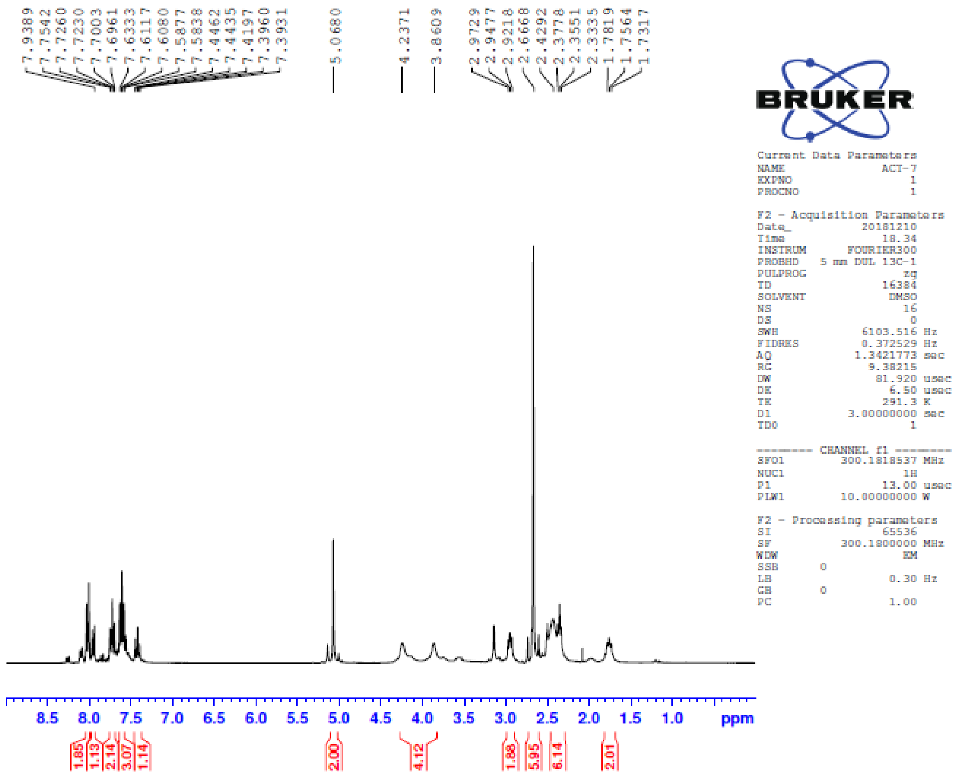

Figure S32. *Compound **4g**-^{1}H-NMR spectrum*

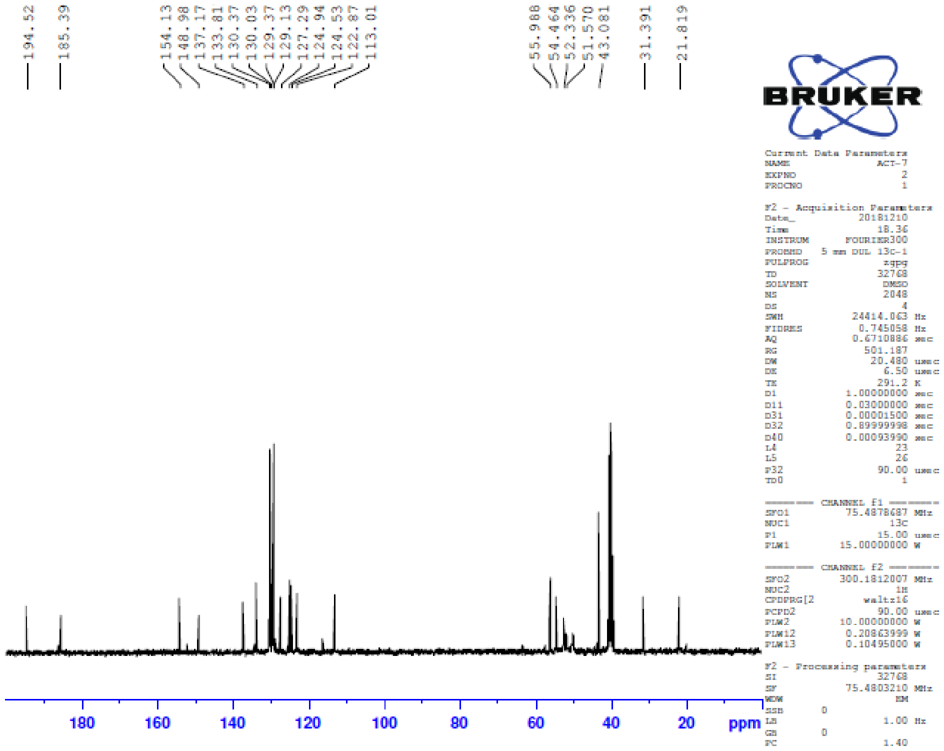

Figure S33. *Compound **4g**-^{13}C-NMR spectrum*

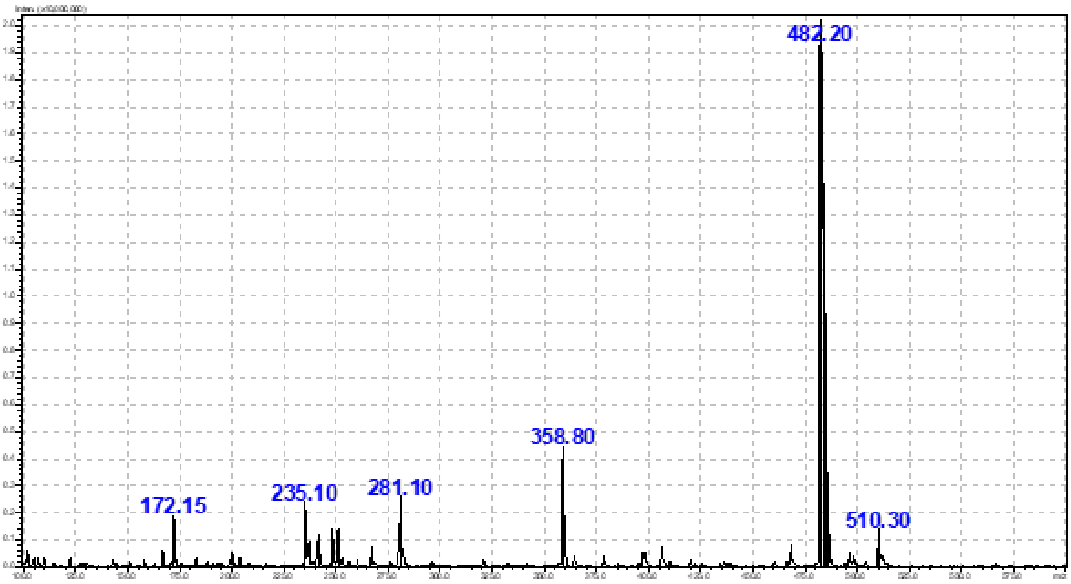

Figure S34. *Compound **4g**-LC-MSMS spectrum*

1.1.1.8. (2-benzoylbenzofuran-3-yl)methyl 4-(furan-2-carbonyl)piperazin-1-dithiocarbamate (4h)

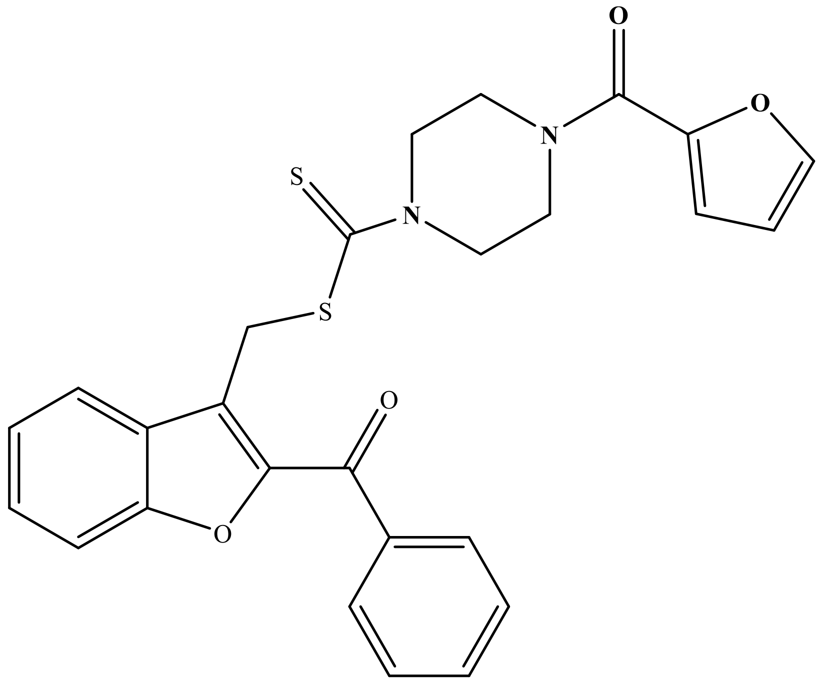

Figure S35. *Compound **4h***

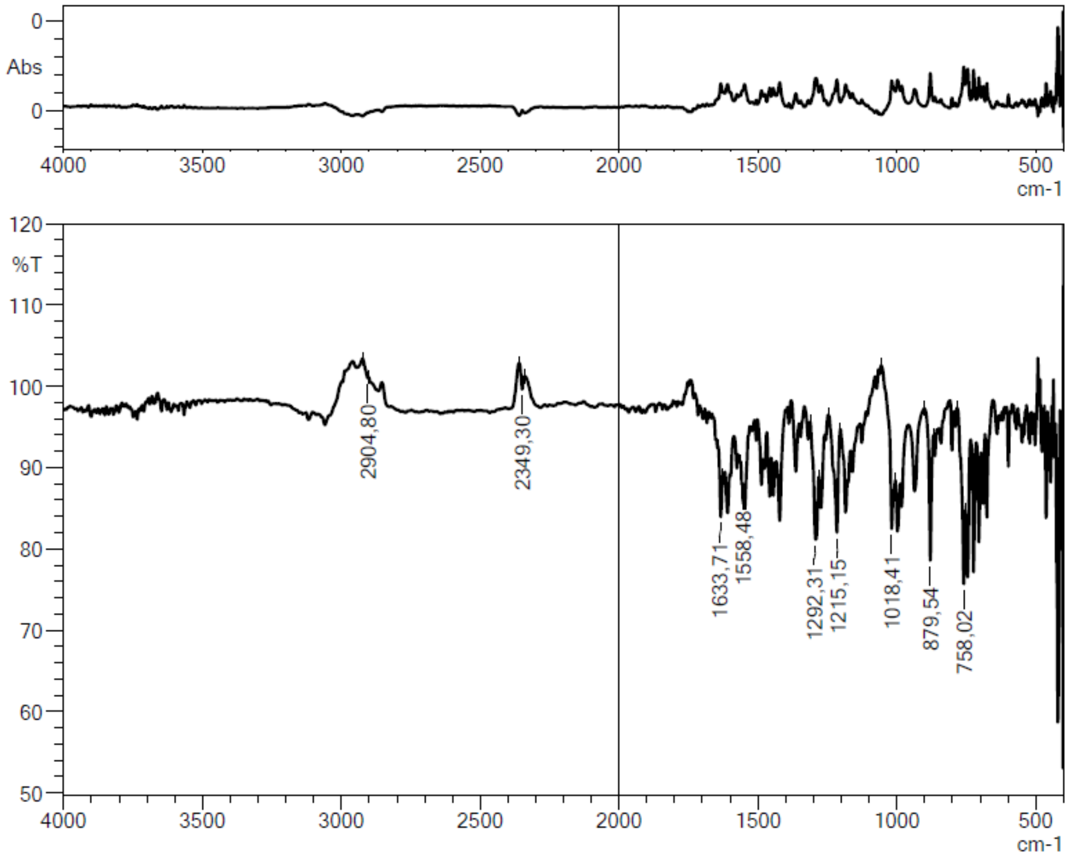

Figure S36. *Compound **4h**-IR spectrum*

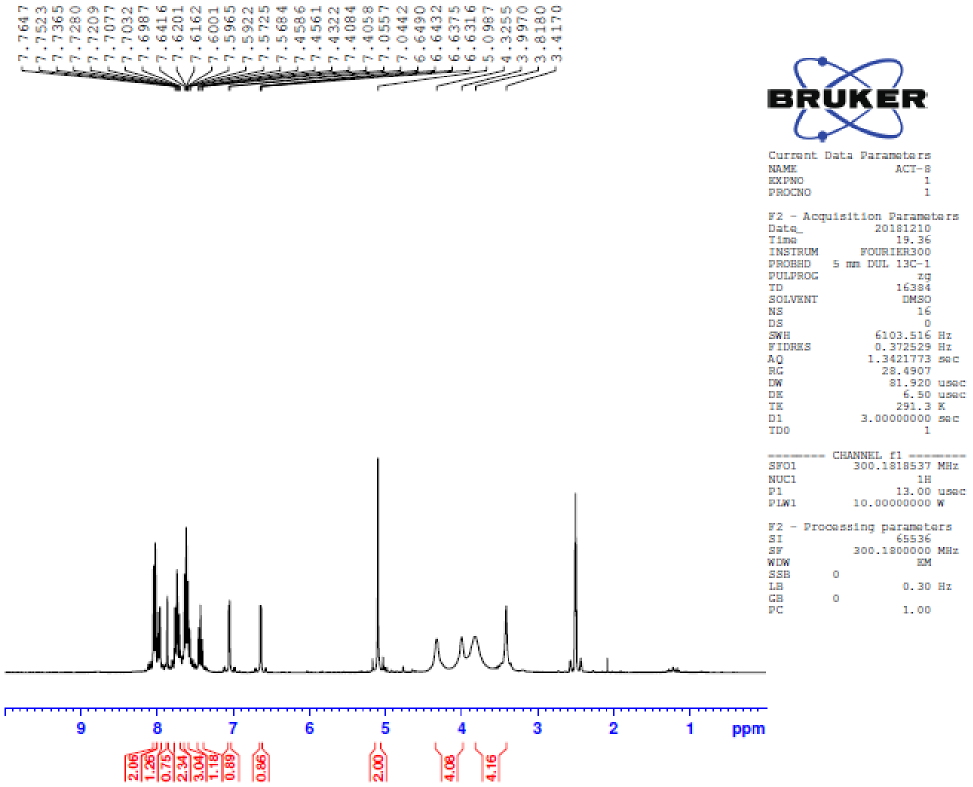

Figure S37. *Compound **4h**-^{1}H-NMR spectrum*

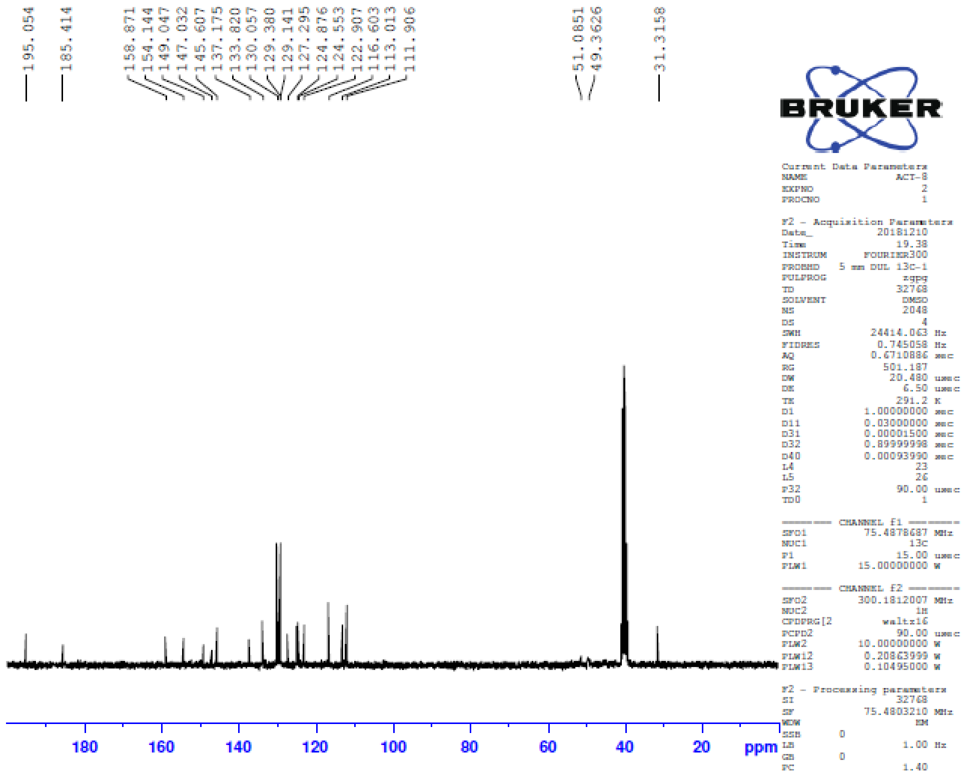

Figure S38. *Compound **4h**-^{13}C-NMR spectrum*

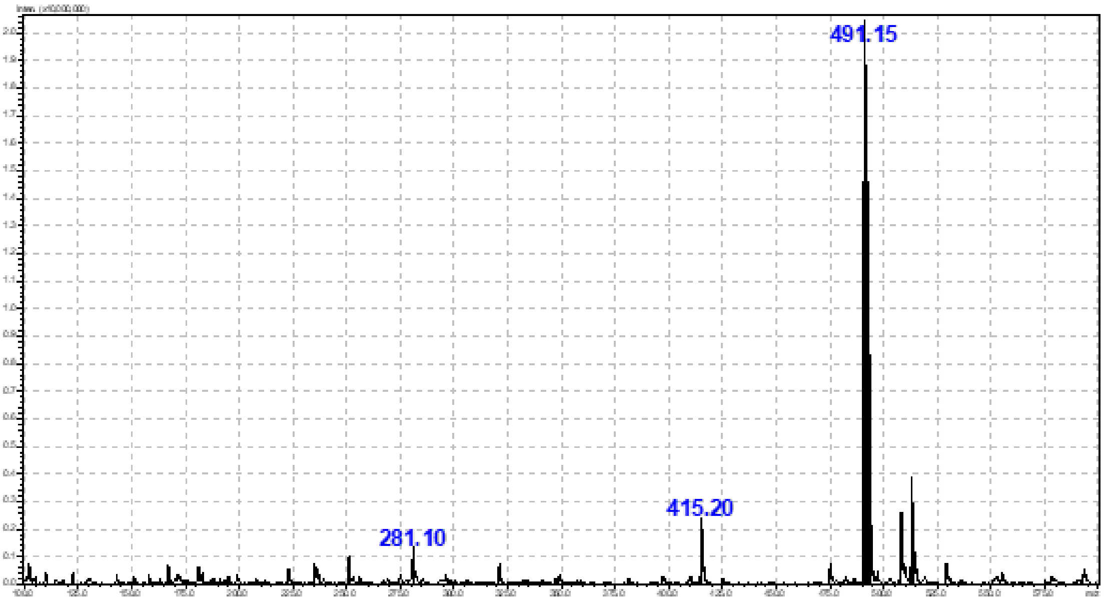

Figure S39. *Compound **4h**-LC-MSMS spectrum*

1.1.1.9. (2-benzoylbenzofuran-3-yl)methyl 4-(pyrimidin-2-il)piperazin-1-dithiocarbamate (4i)

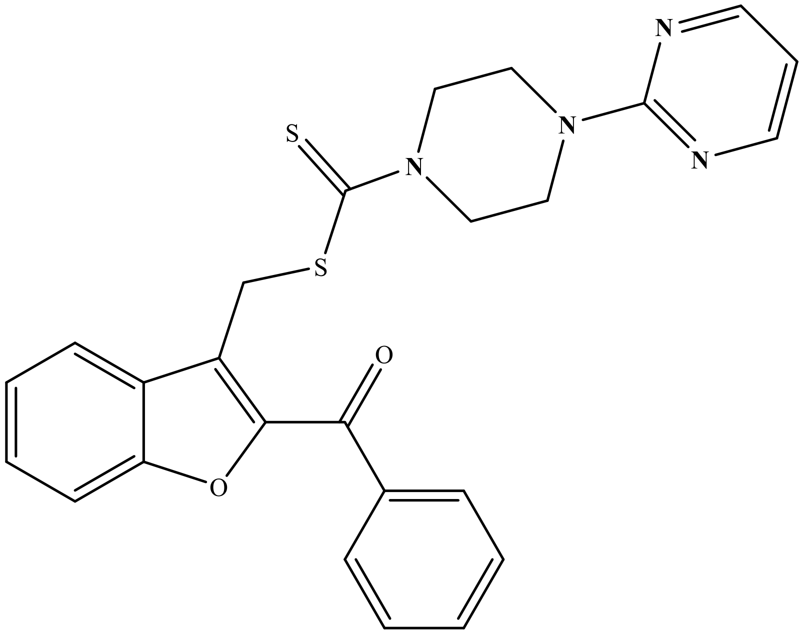

Figure S40. *Compound 4i*

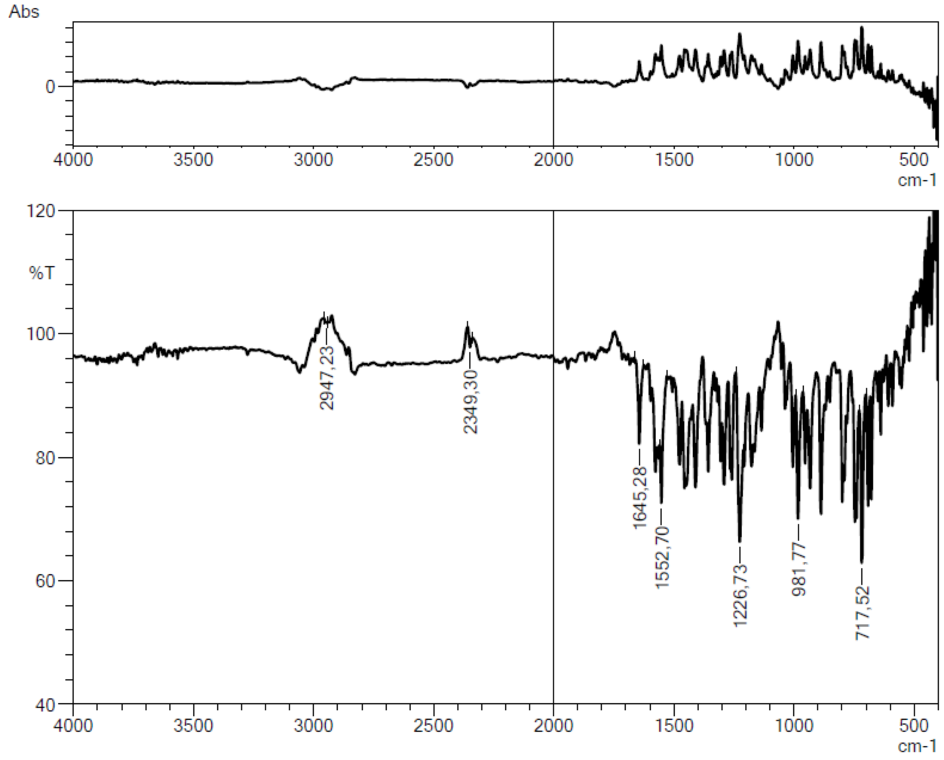

Figure S41. *Compound 4i-IR spectrum*

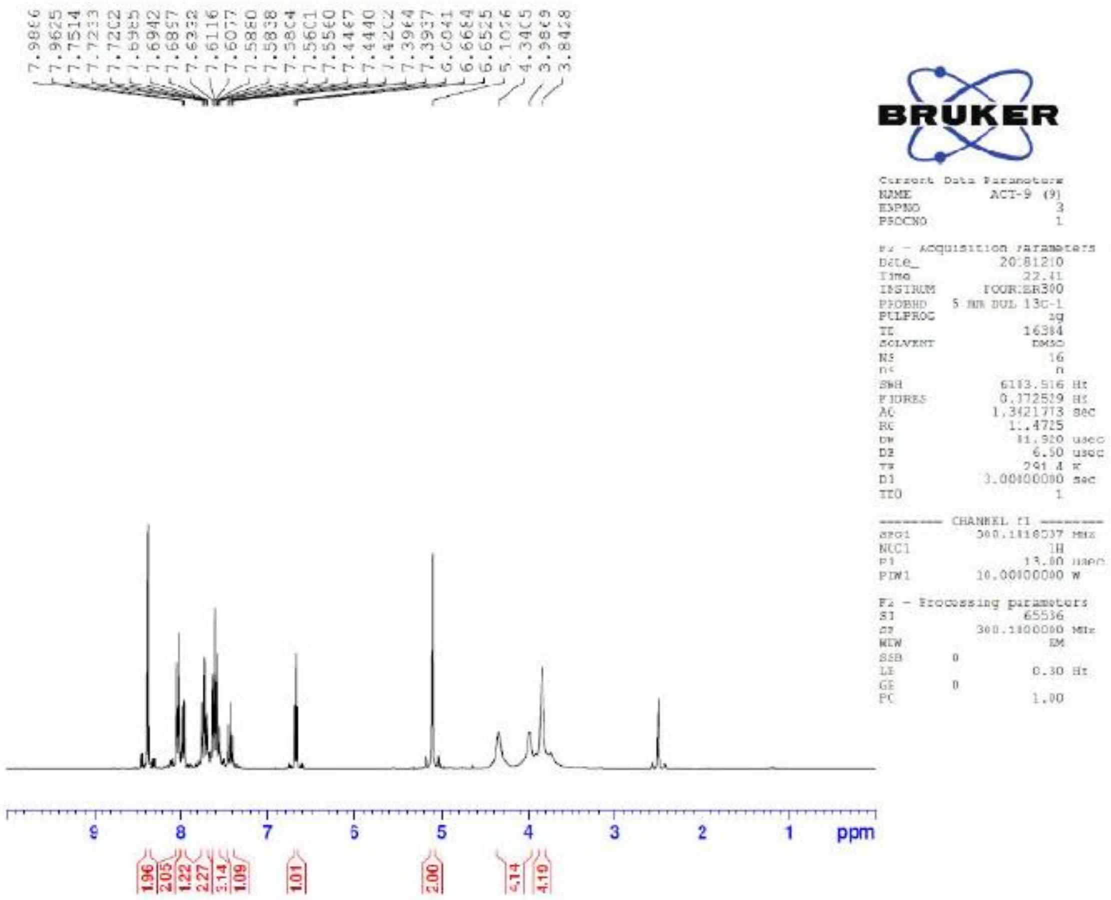

Figure S42. *Compound* ***4i***-*^{1}H-NMR spectrum*

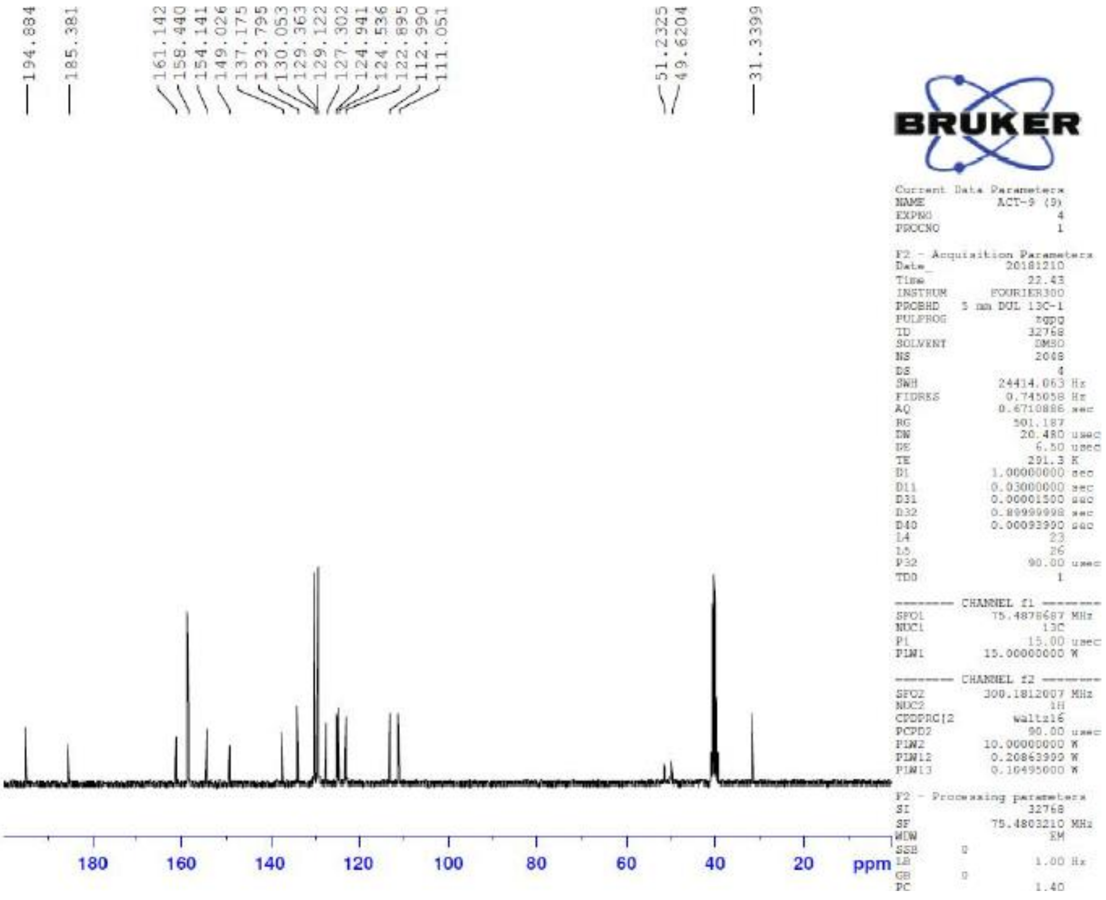

Figure S43. *Compound **4i**-^{13}C-NMR spectrum*

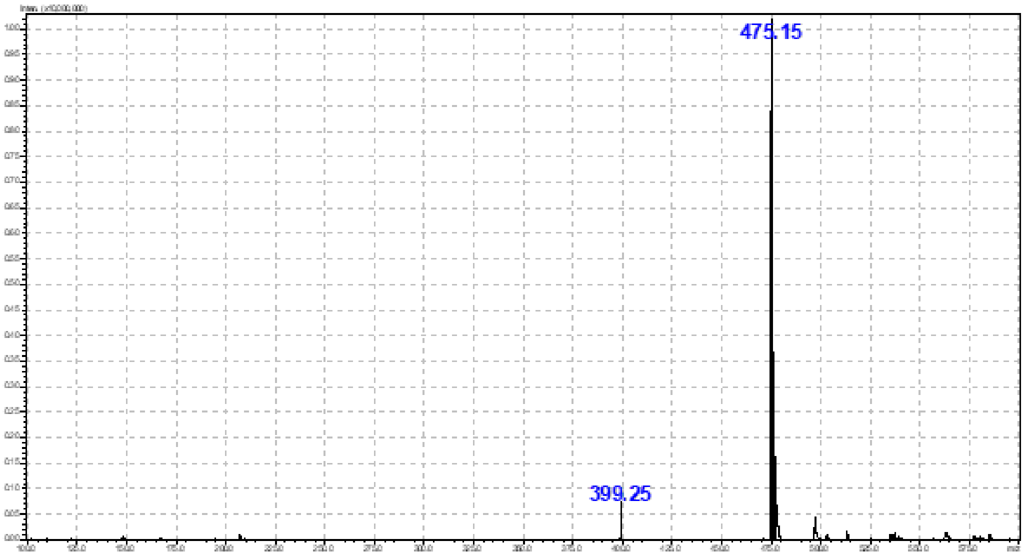

Figure S44. *Compound **4i**-LC-MSMS spectrum*

1.1.1.10. (2-benzoylbenzofuran-3-yl)methyl 4-phenylpiperazin-1-dithiocarbamate (4j)

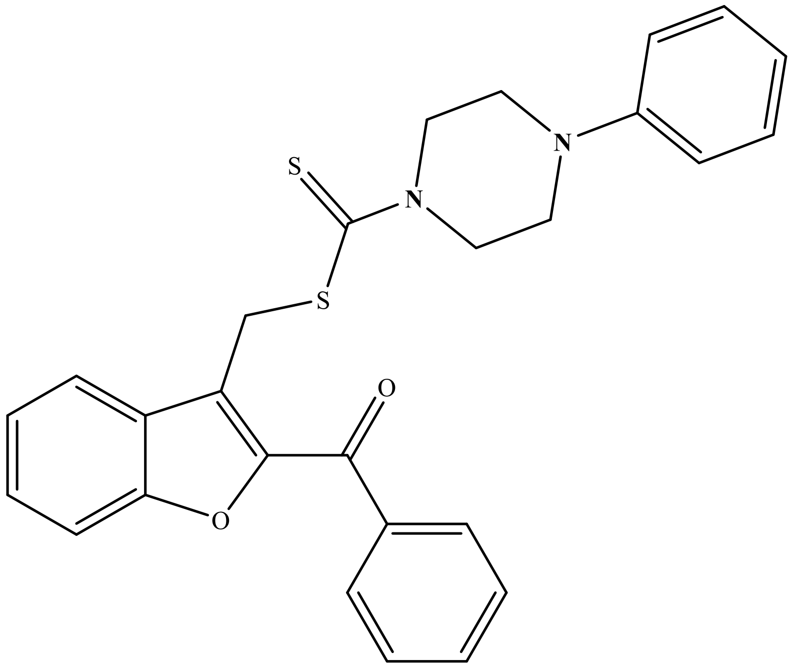

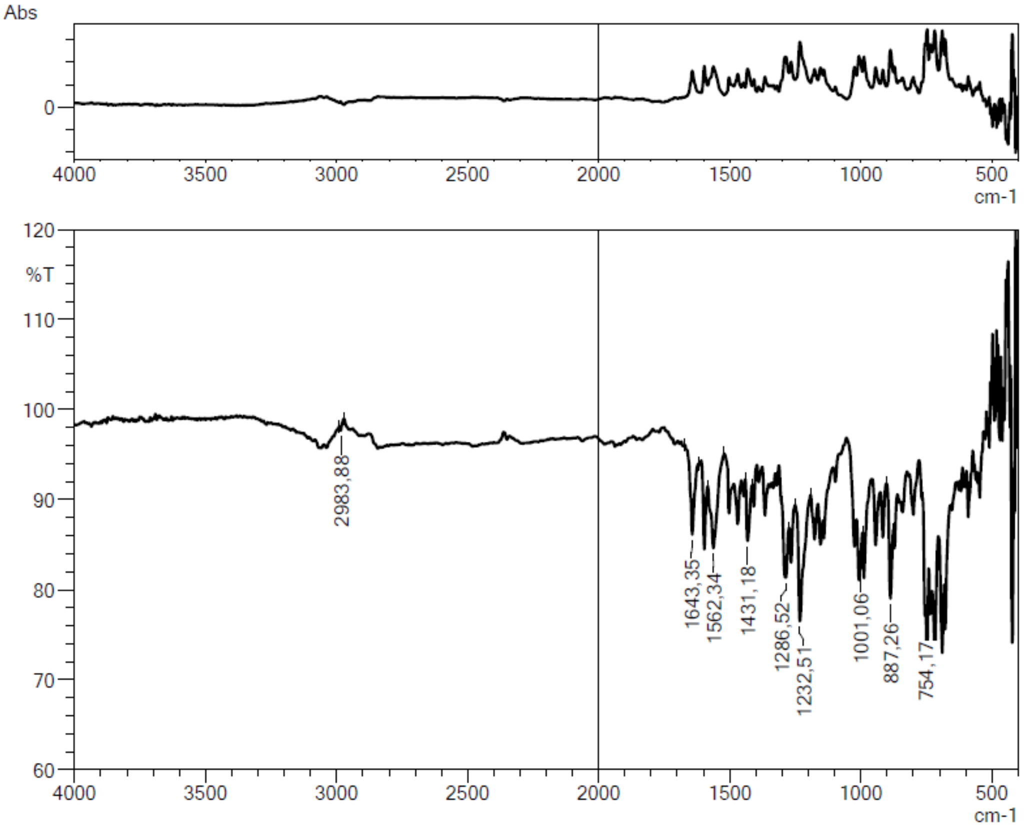

Figure S45. *Compound **4j**-IR spectrum*

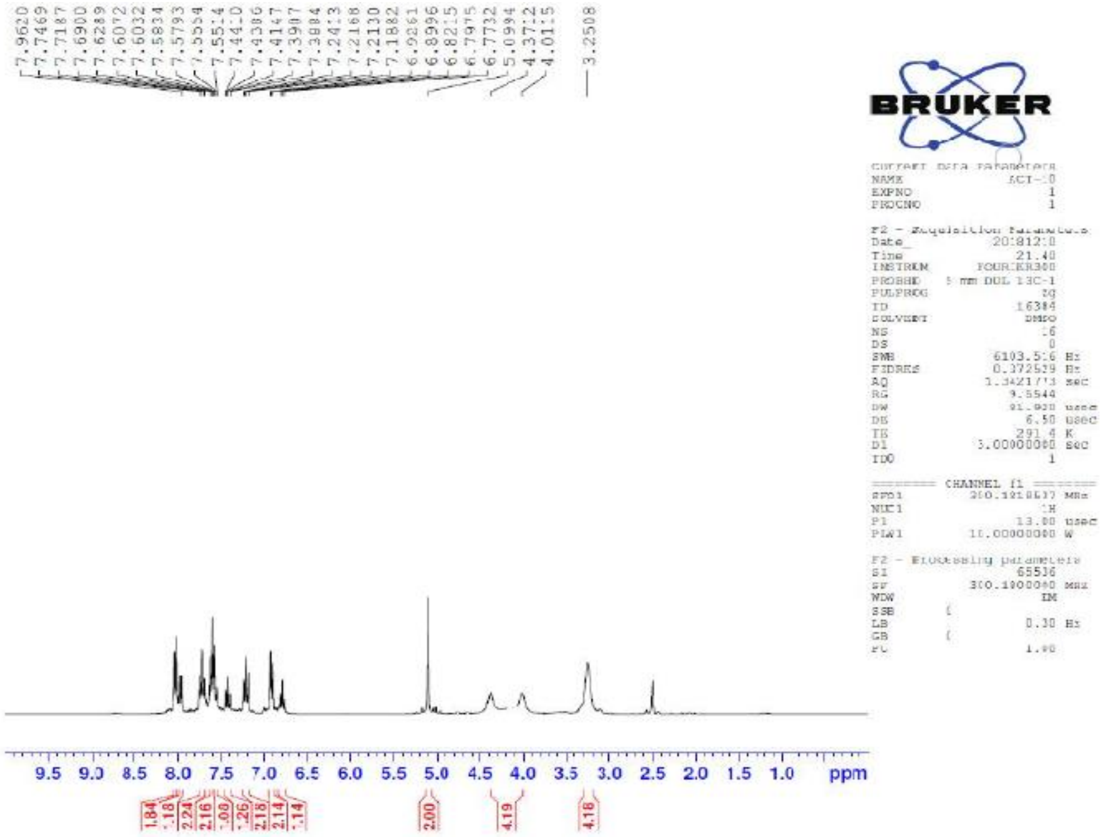

Figure S46. *Compound **4j**-^{1}H-NMR spectrum*

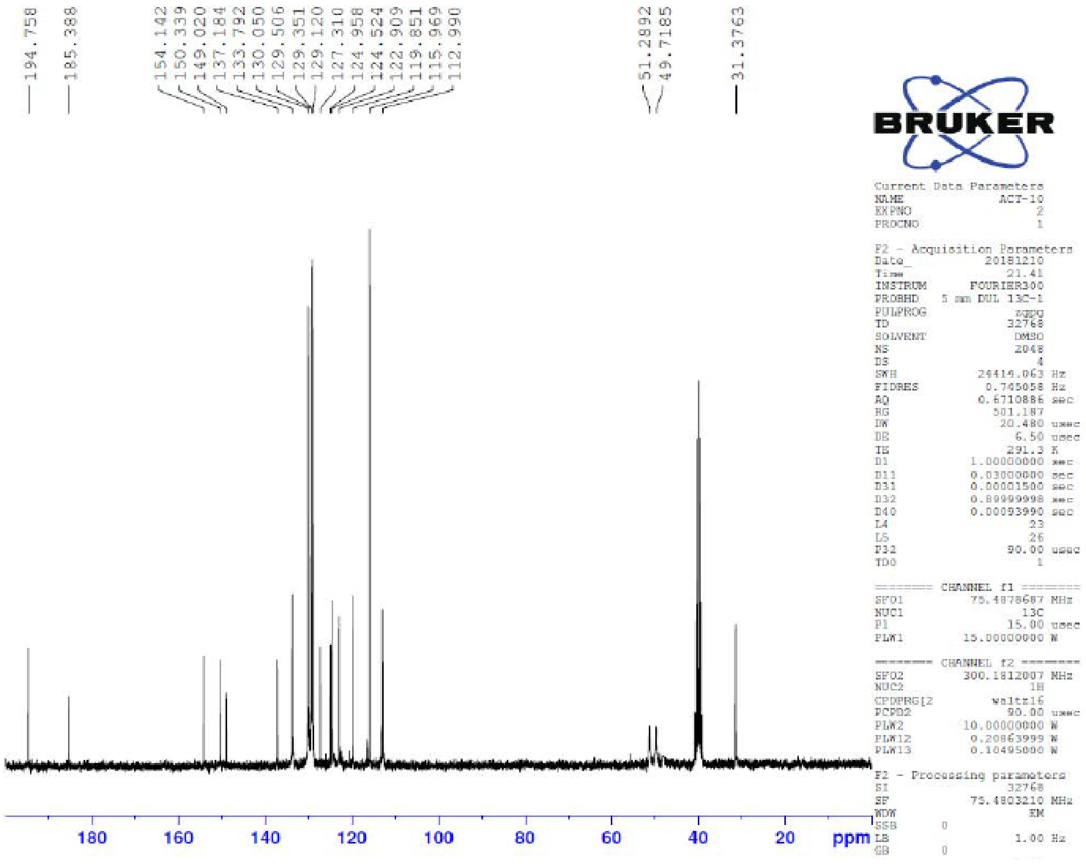

Figure S47. *Compound **4j**-^{13}C-NMR spectrum*

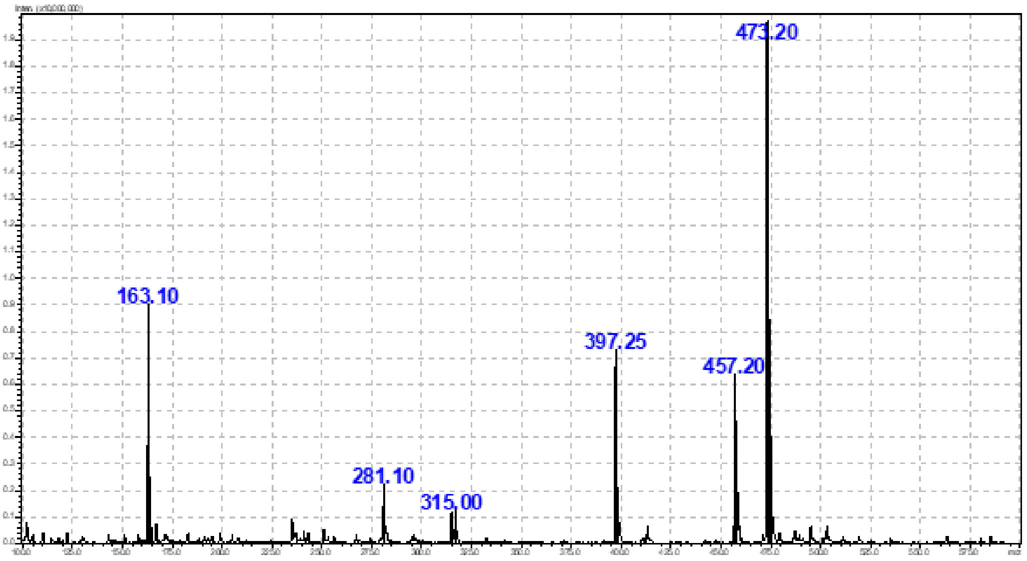

Figure S48. *Compound **4j**-LC-MSMS spectrum*

1.1.1.11. (2-benzoylbenzofuran-3-yl)methyl 4-(4-nitrophenyl)piperazin-1-dithiocarbamate (4k)

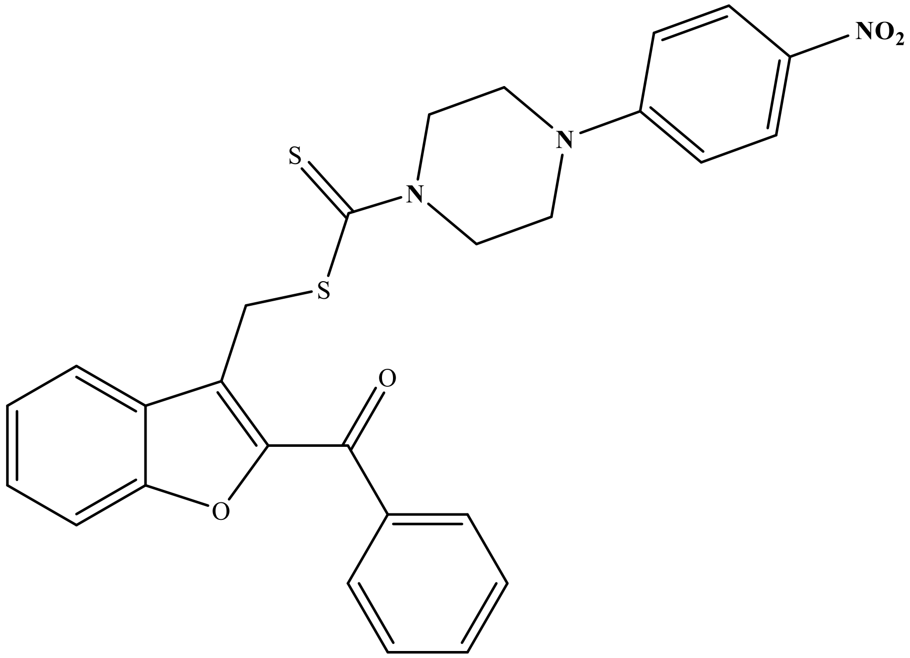

Figure S49. *Compound **4k***

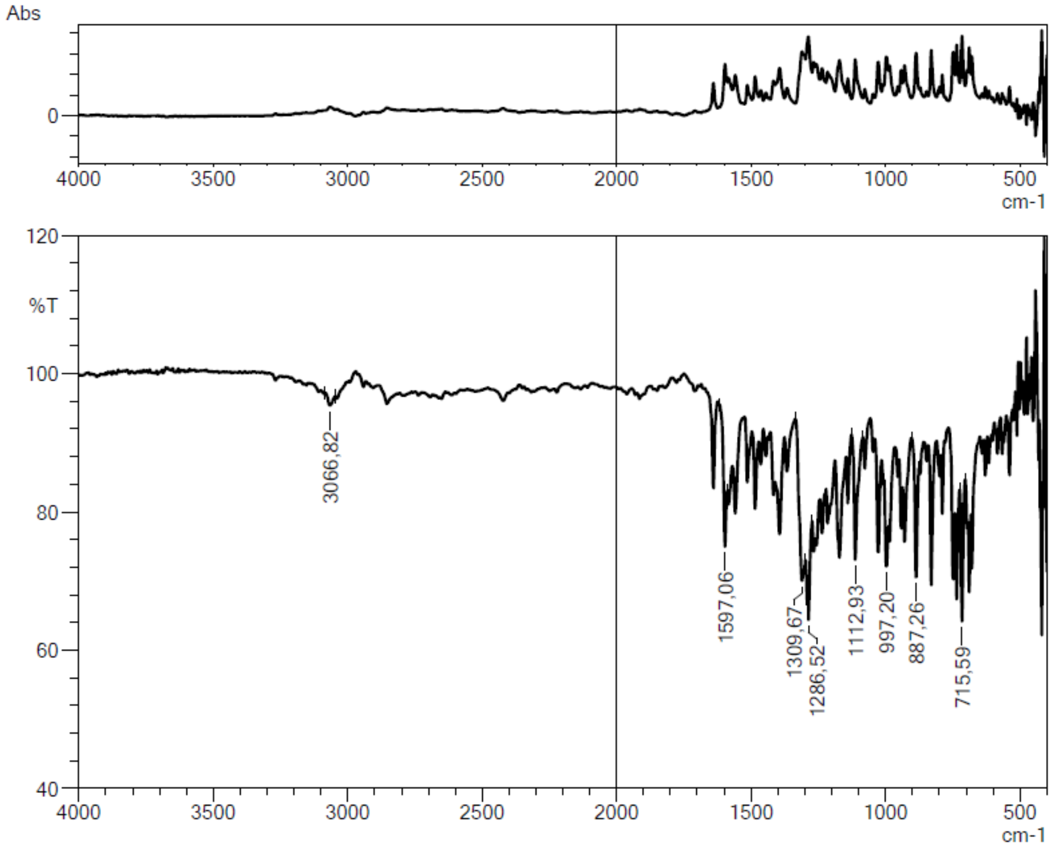

Figure S50. *Compound **4k**-IR spectrum*

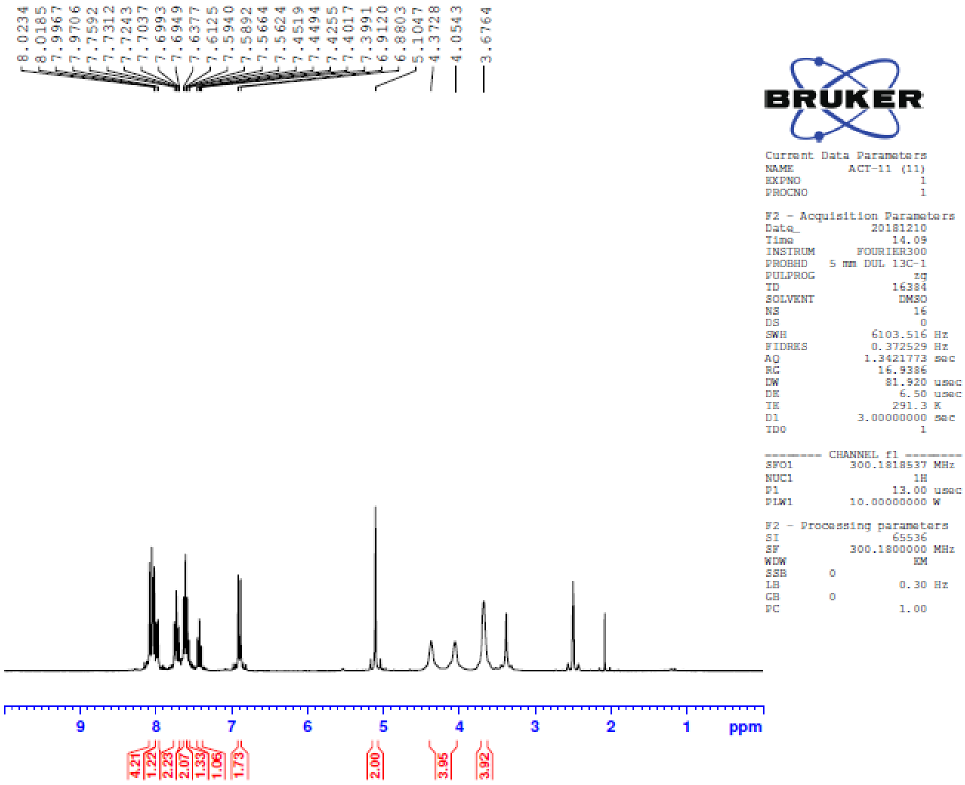

Figure S51. *Compound* ***4k***-^{1}*H-NMR spectrum*

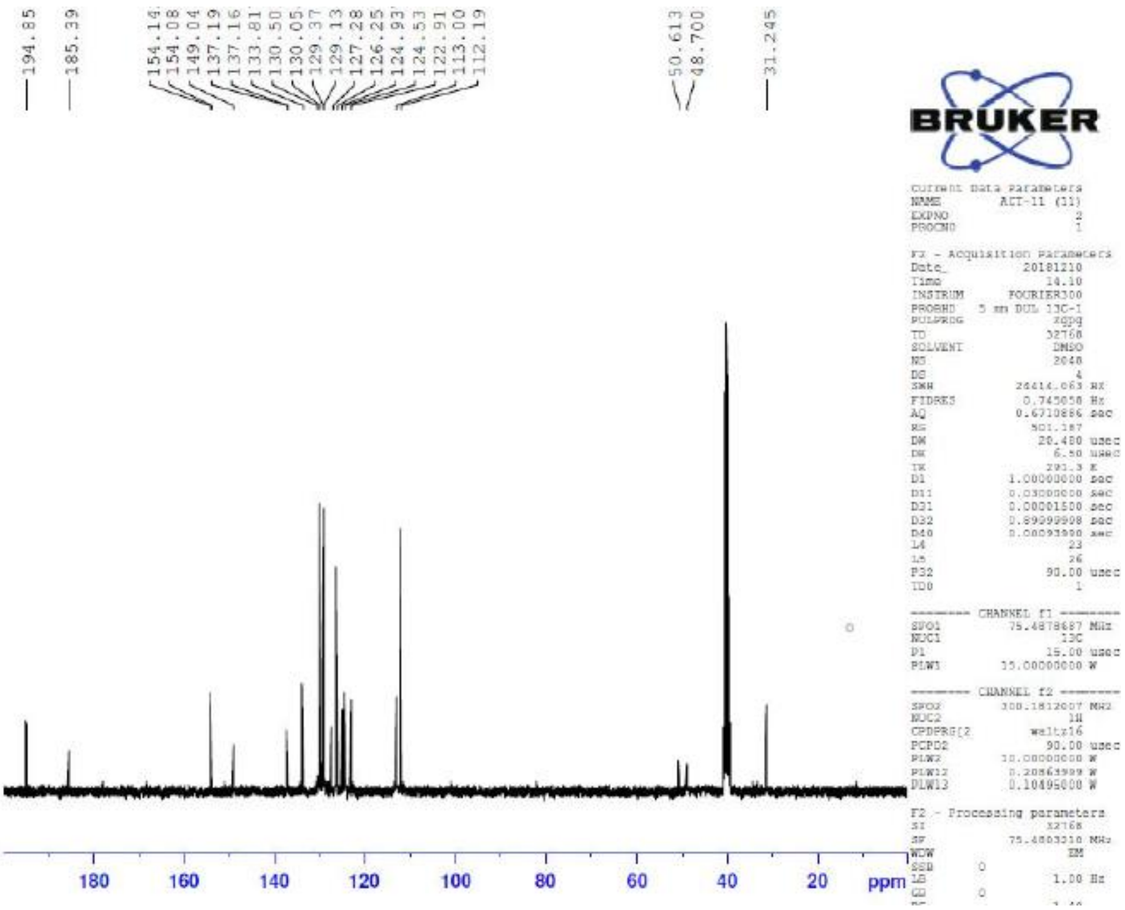

Figure S52. *Compound **4k**-^{13}C-NMR spectrum*

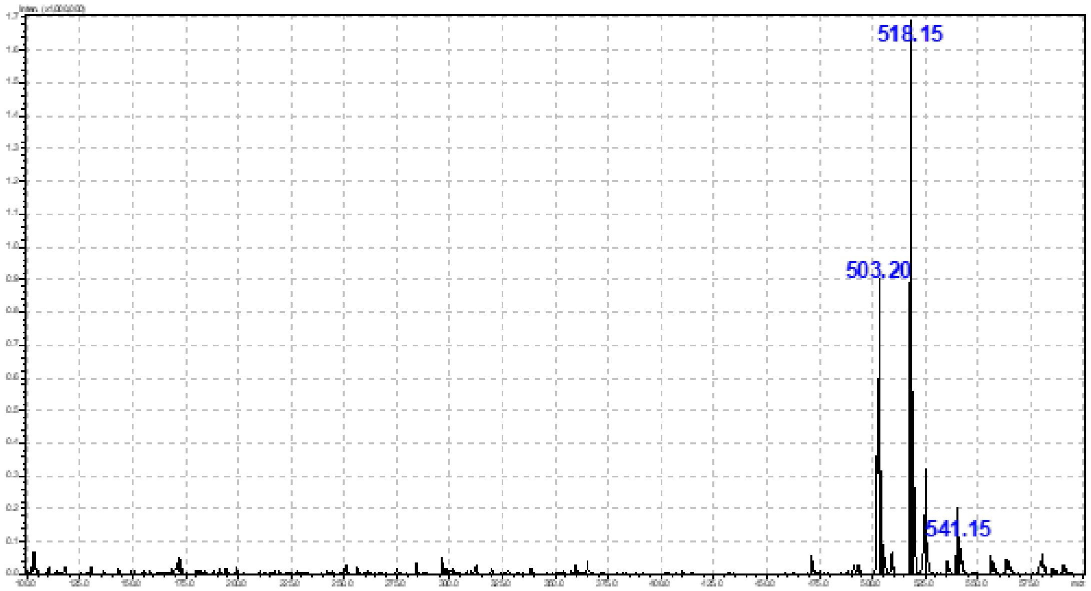

Figure S53. *Compound **4k**-LC-MSMS spectrum*

1.1.1.12. (2-benzoylbenzofuran-3-yl)methyl 4-(4-methoxyphenyl)piperazin-1-dithiocarbamate (4l)

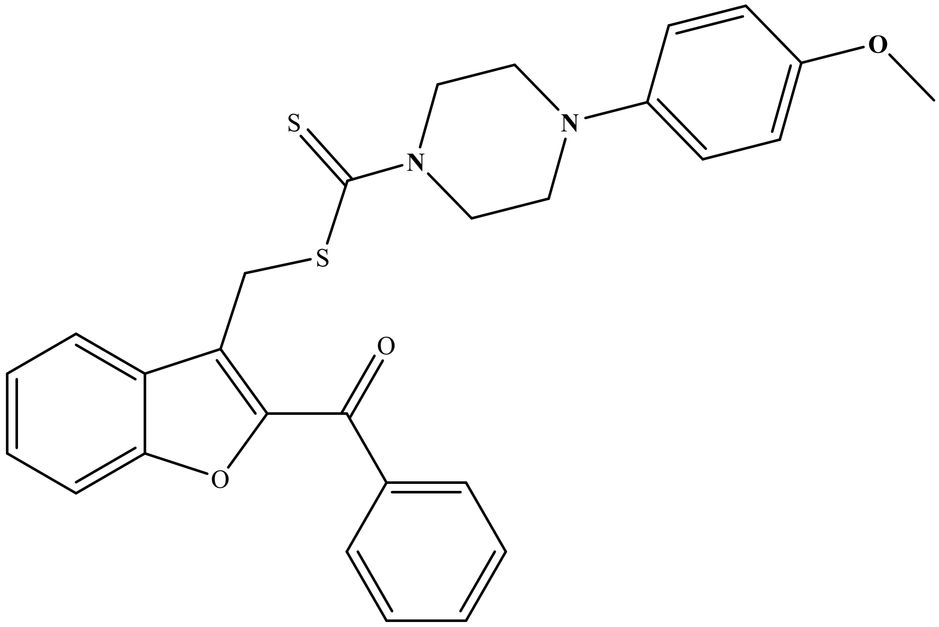

Figure S54. *Compound **4l***

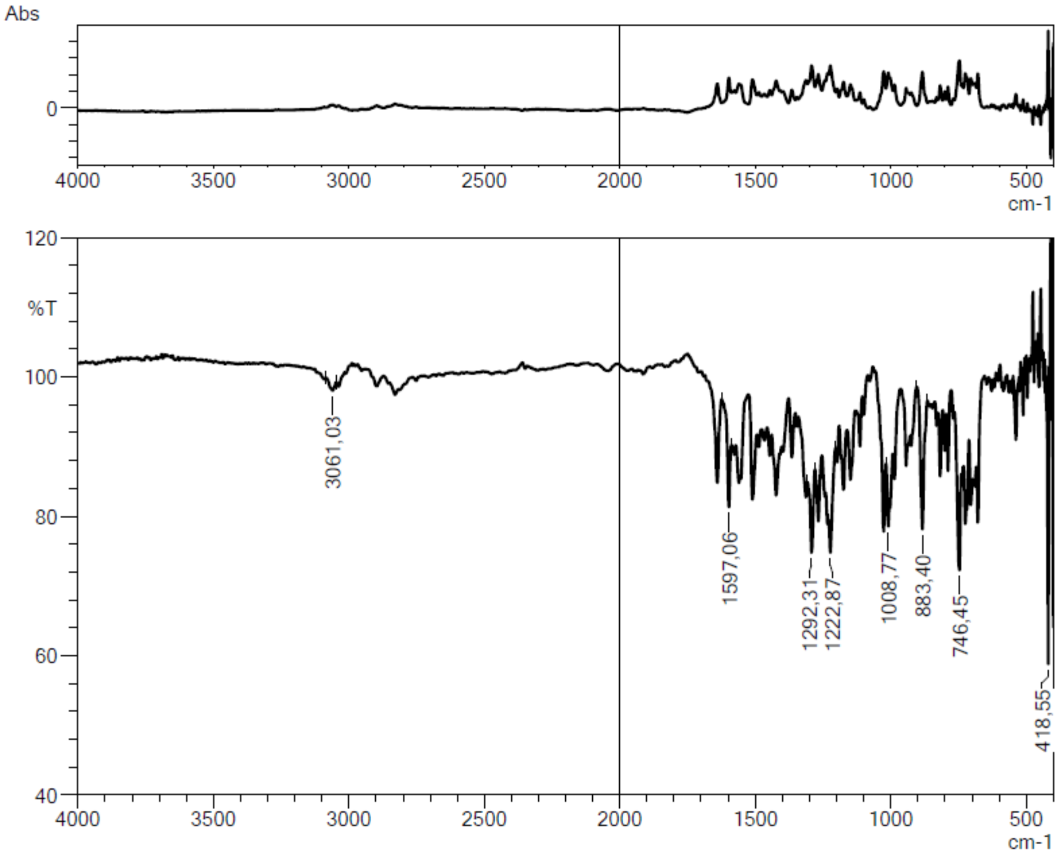

Figure S55. *Compound **4l**-IR spectrum*

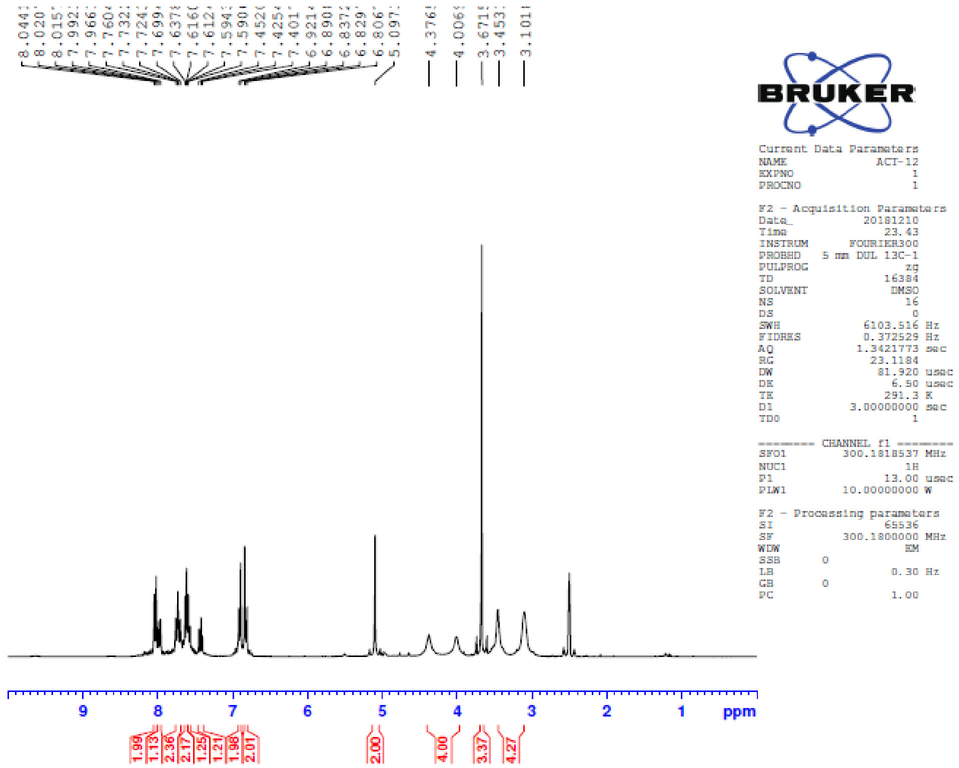

Figure S56. *Compound **4l**-^{1}H-NMR spectrum*

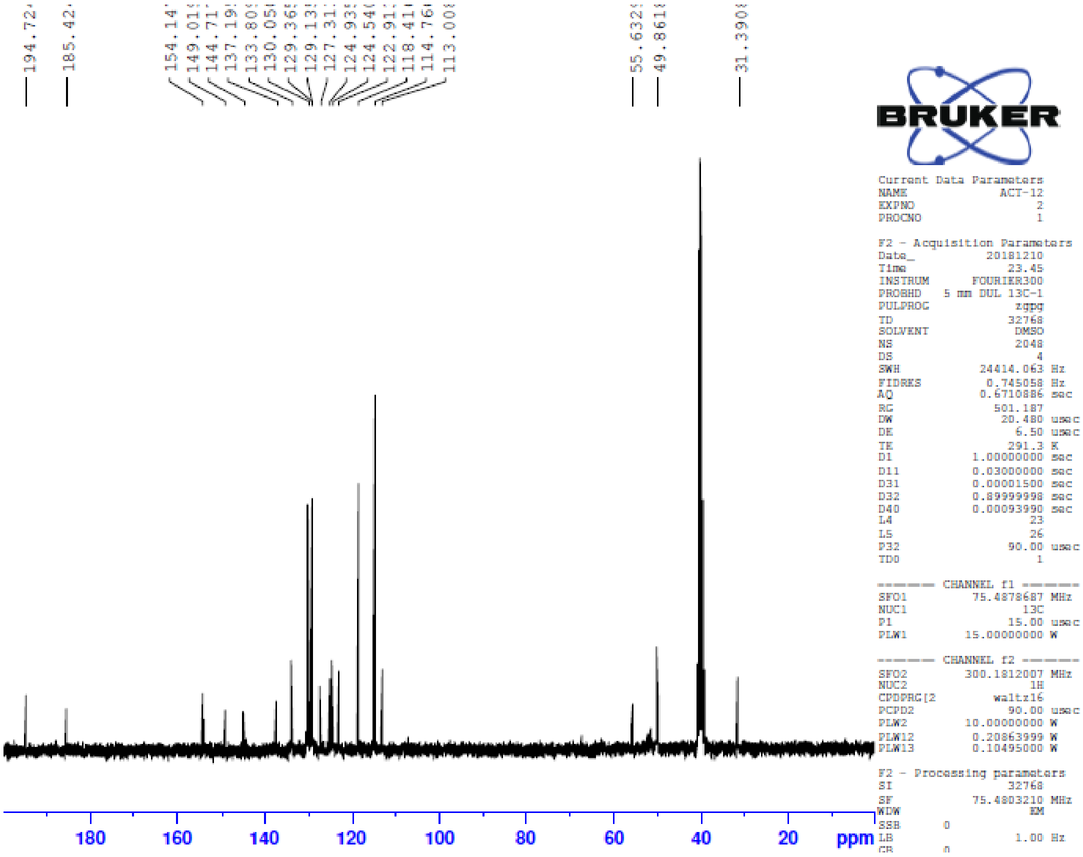

Figure S57. *Compound **4l**-^{13}C-NMR spectrum*

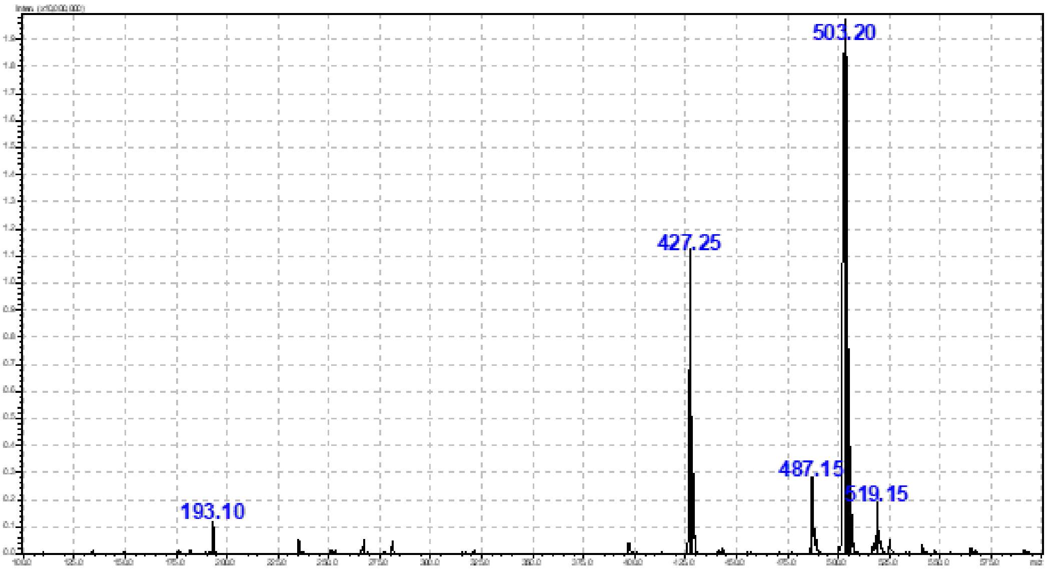

Figure S58. *Compound **4l**-LC-MSMS spectrum*

1.1.1.13. (2-benzoylbenzofuran-3-yl)methyl 4-(2-hydroxyetil)piperazin-1-dithiocarbamate (4m)

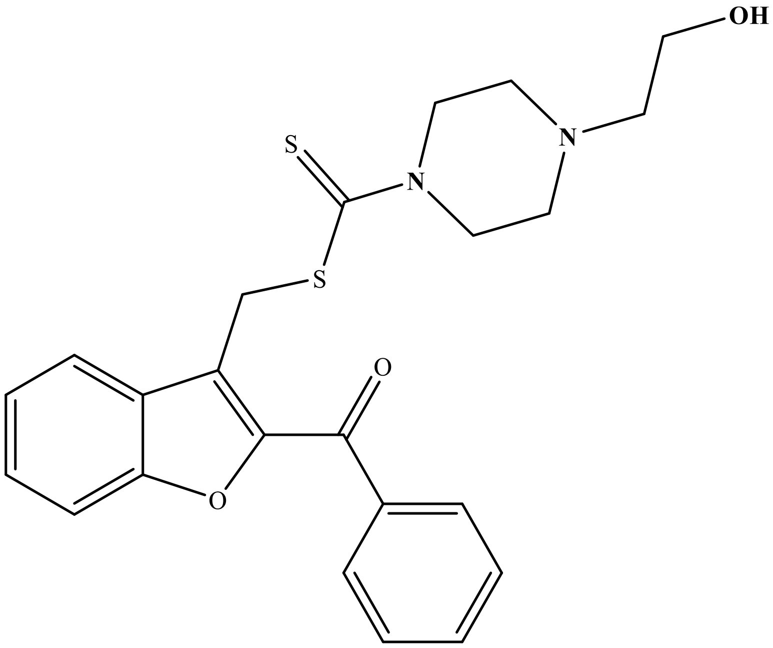

Figure S59. *Compound **4m***

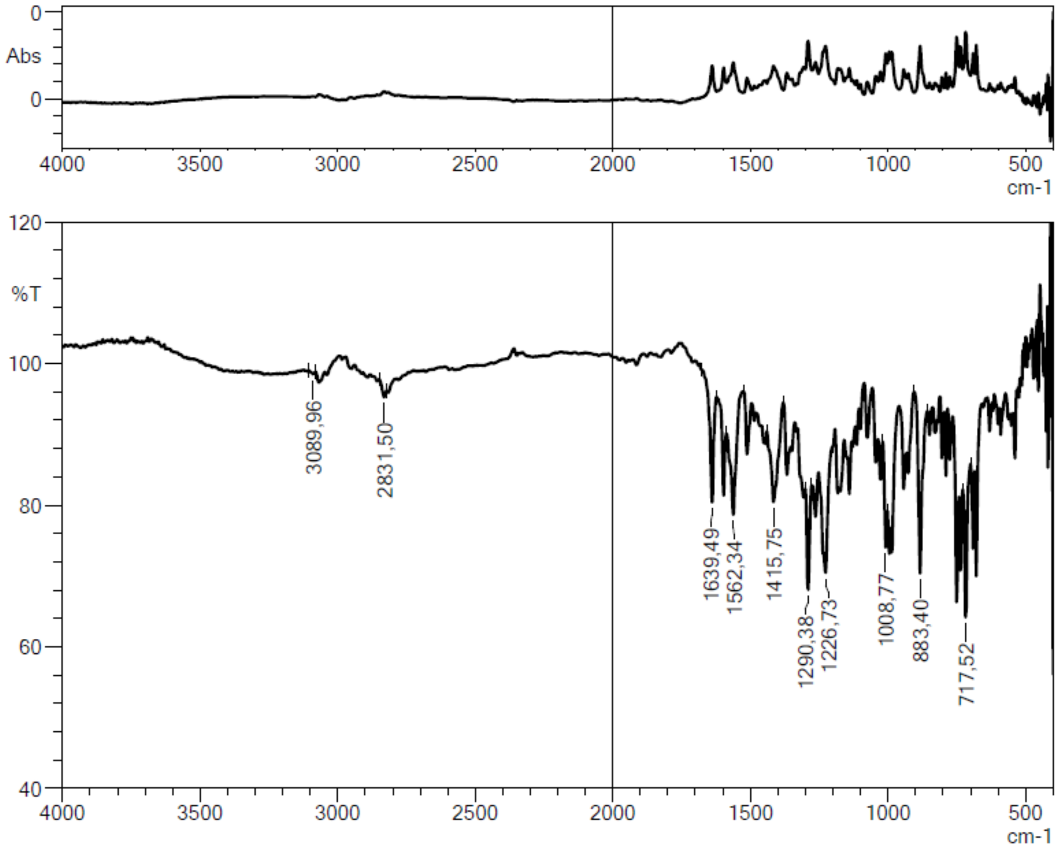

Figure S60. *Compound **4m**-IR spectrum*

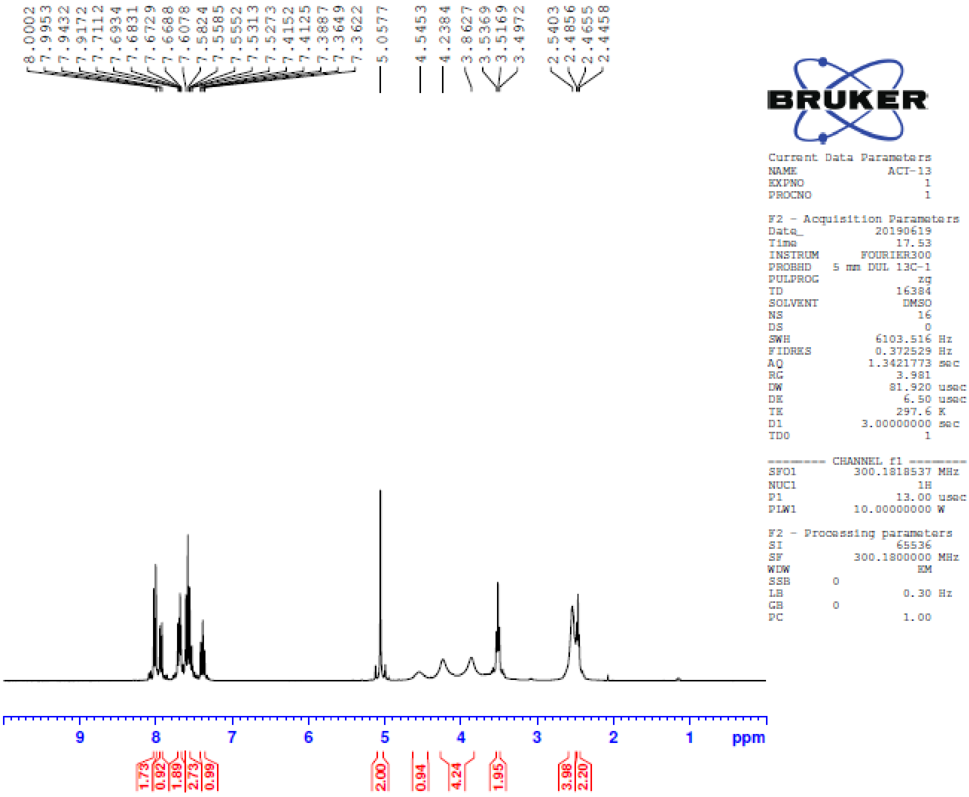

Figure S61. *Compound **4m**-^{1}H-NMR spectrum*

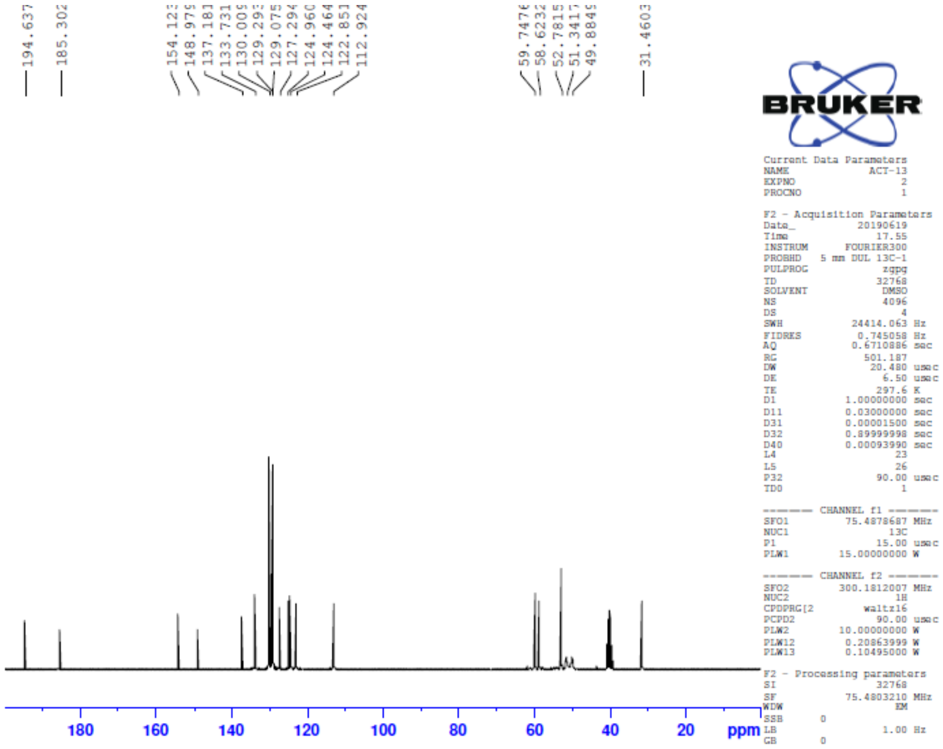

Figure S62. *Compound **4m**-^{13}C-NMR spectrum*

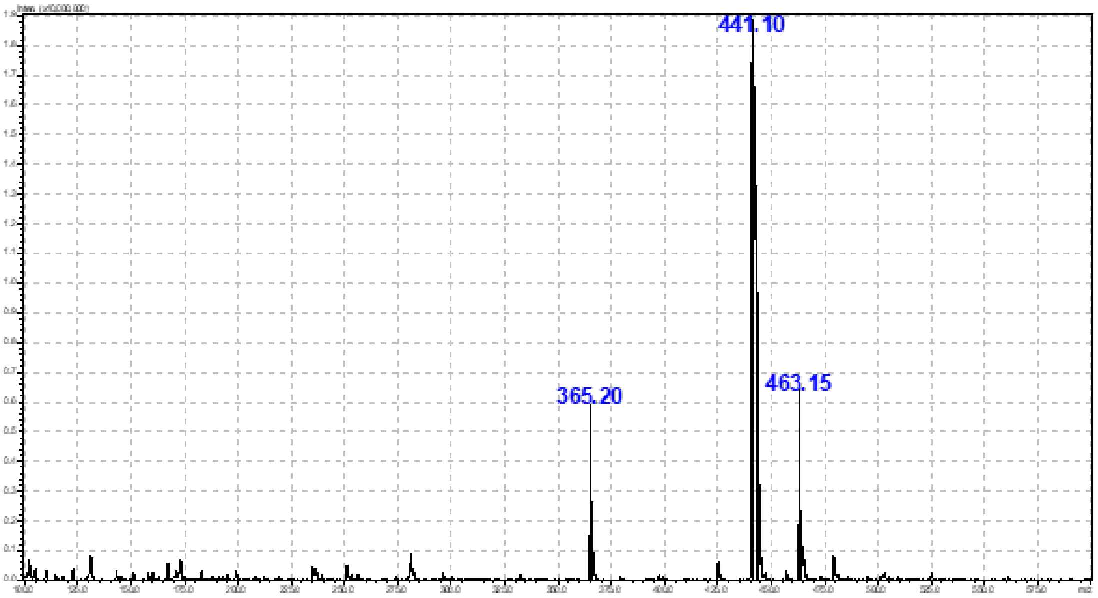

Figure S63. *Compound* ***4m****-LC-MSMS spectrum*

1.1.1.14. (2-benzoylbenzofuran-3-yl)methyl 4-(4-clorophenyl)piperazin-1-dithiocarbamate (4n)

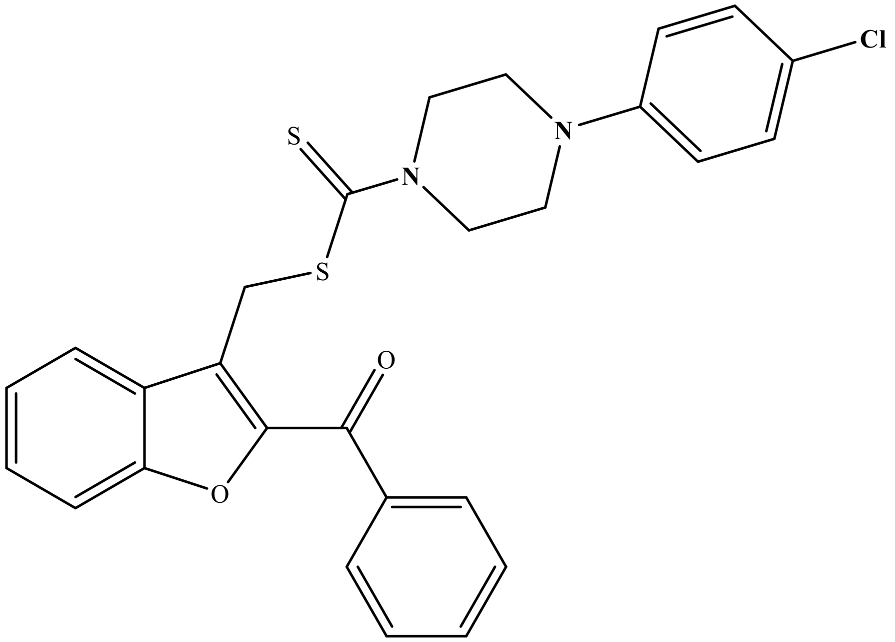

Figure S64. *Compound* ***4n***

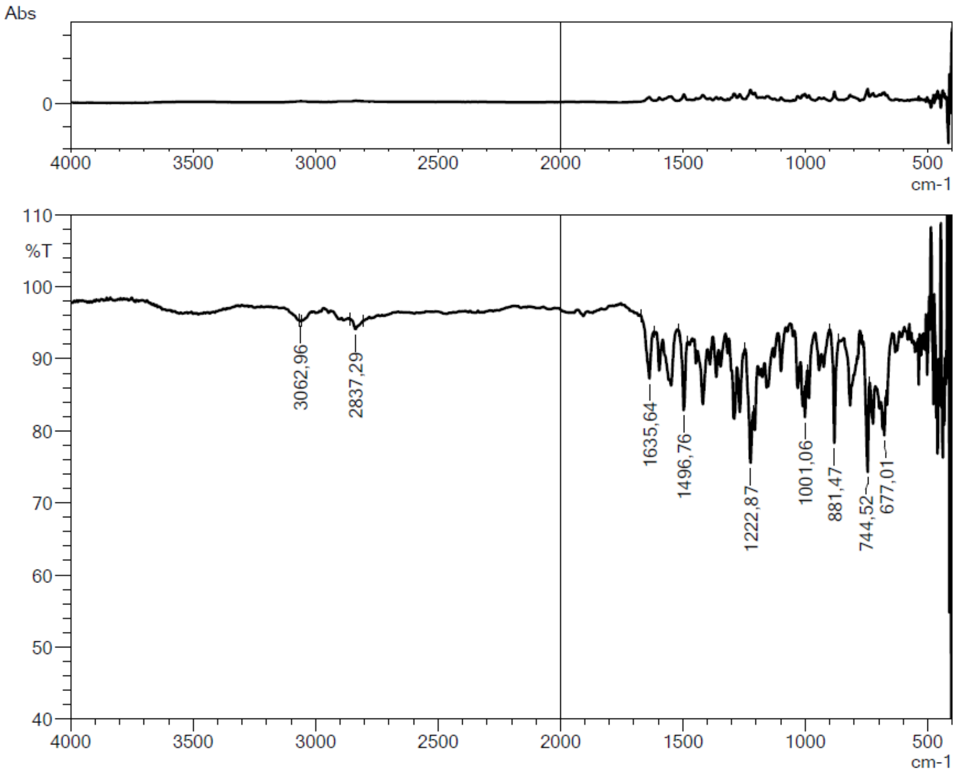

Figure S65. *Compound* ***4n****-IR spectrum*

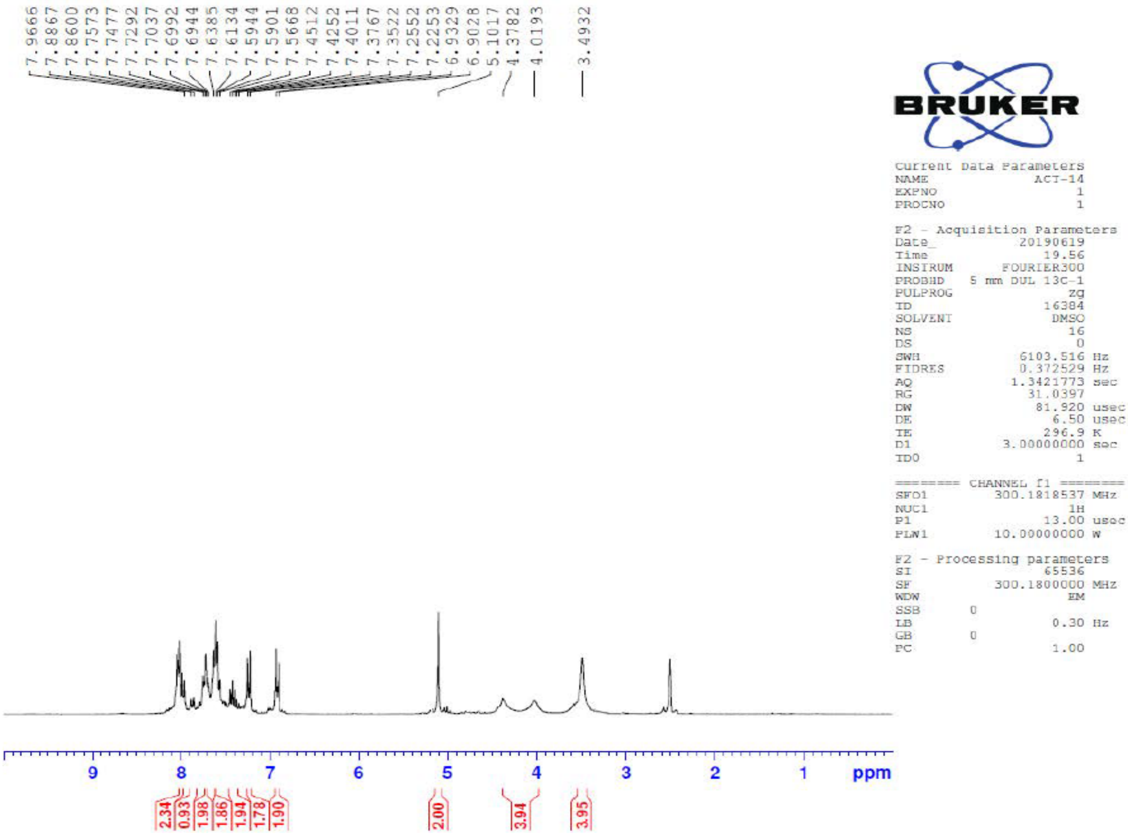

Figure S66. *Compound **4n**-^{1}H-NMR spectrum*

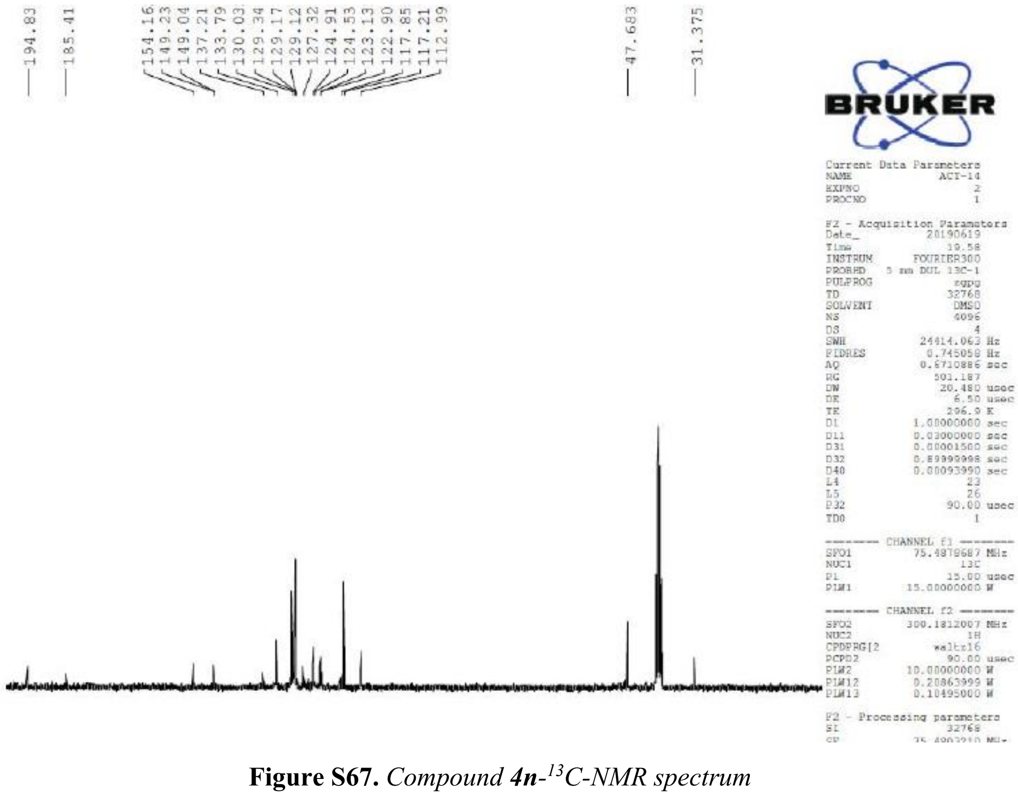

Figure S67. *Compound **4n**-^{13}C-NMR spectrum*

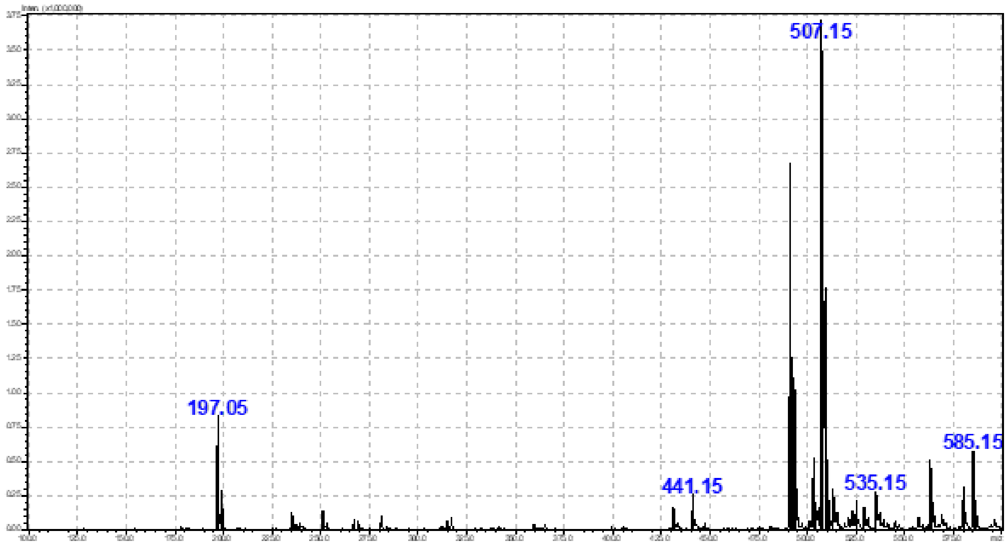

Figure S68. *Compound **4n**-LC-MSMS spectrum*

1.1.1.15. (2-benzoylbenzofuran-3-yl)methyl 4-(4-flourophenyl)piperazin-1-dithiocarbamate (4o)

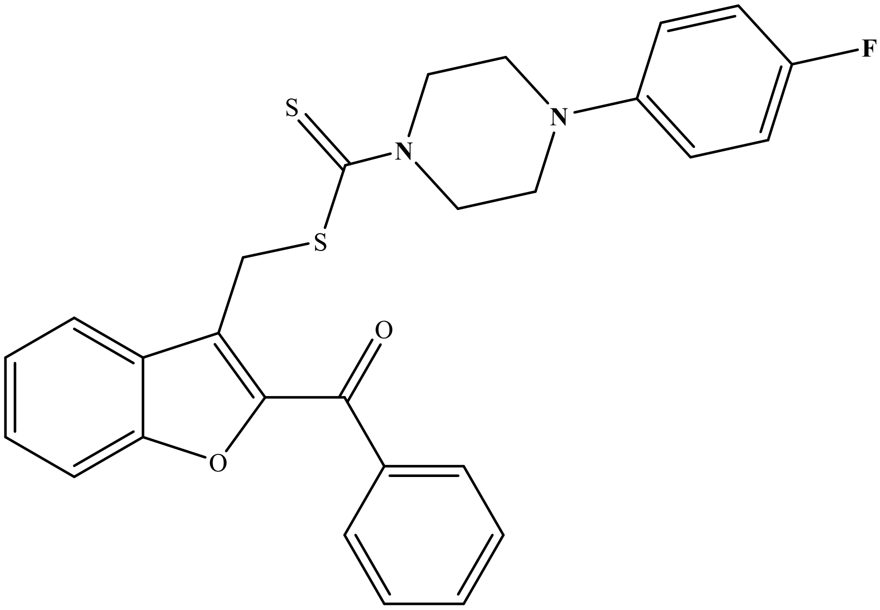

Figure S69. *Compound* ***4o***

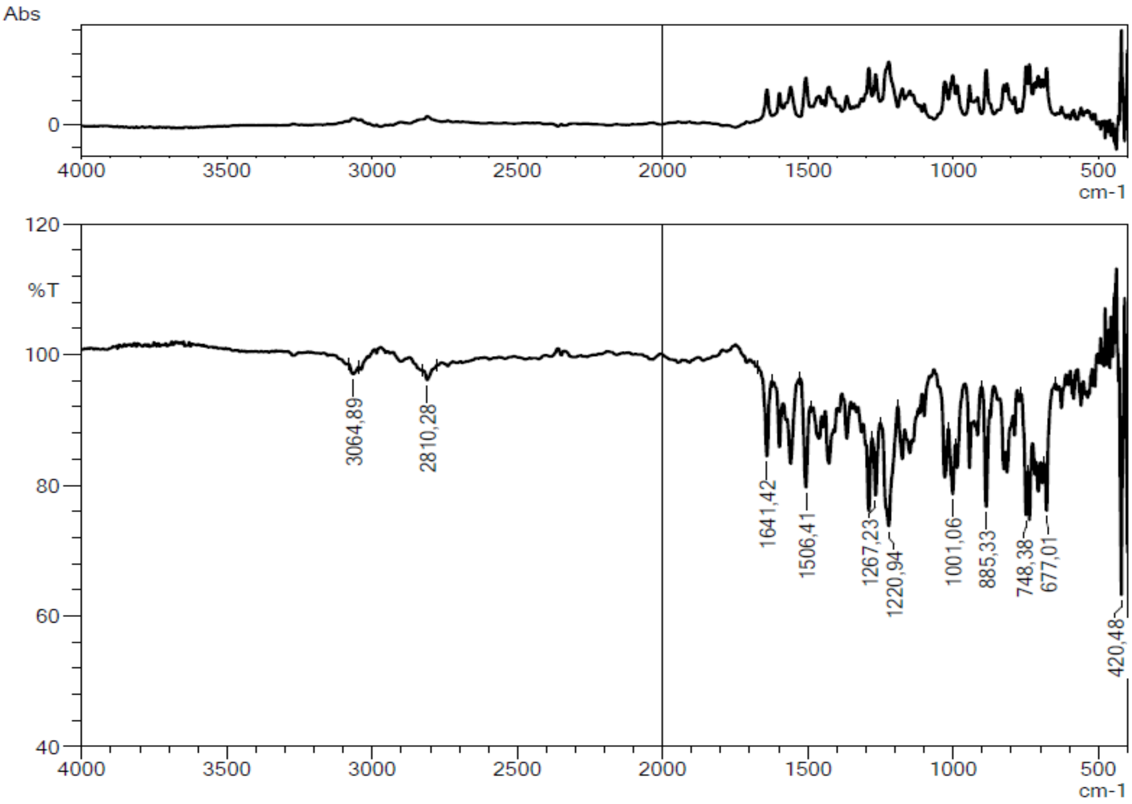

Figure S70. *Compound* ***4o****-IR spectrum*

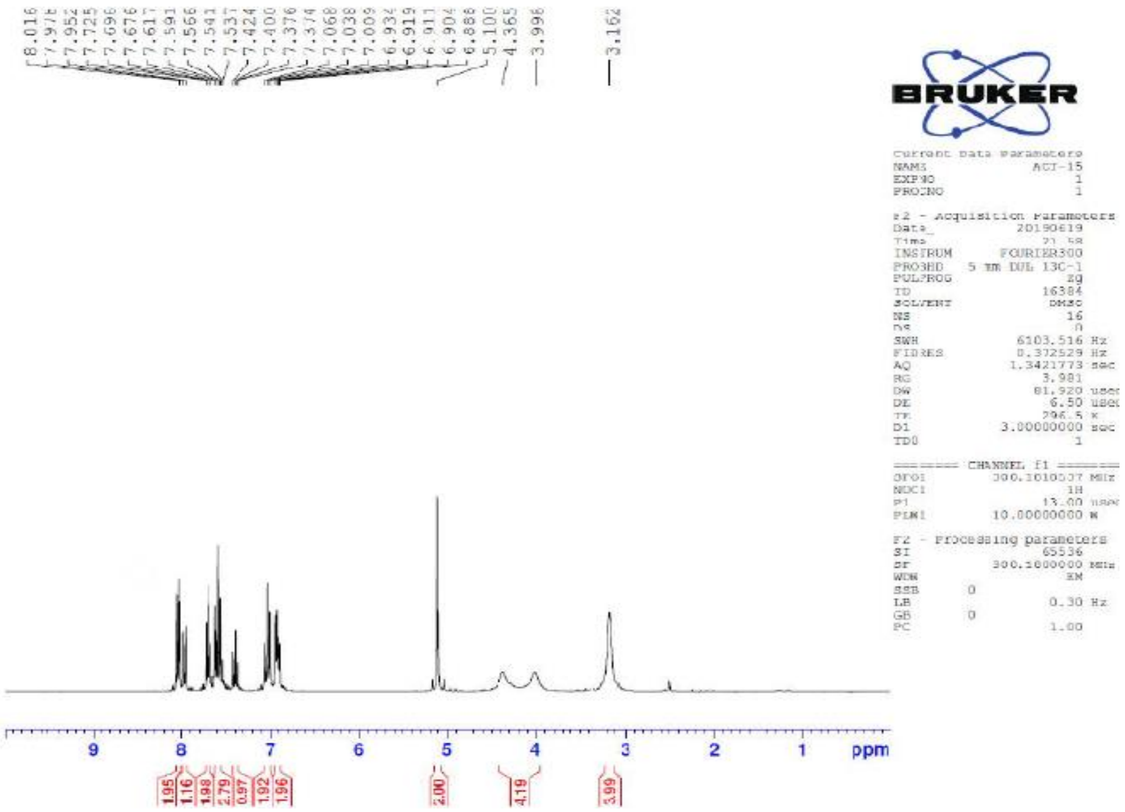

Figure S71. *Compound **4o**-*[1]*H-NMR spectrum*

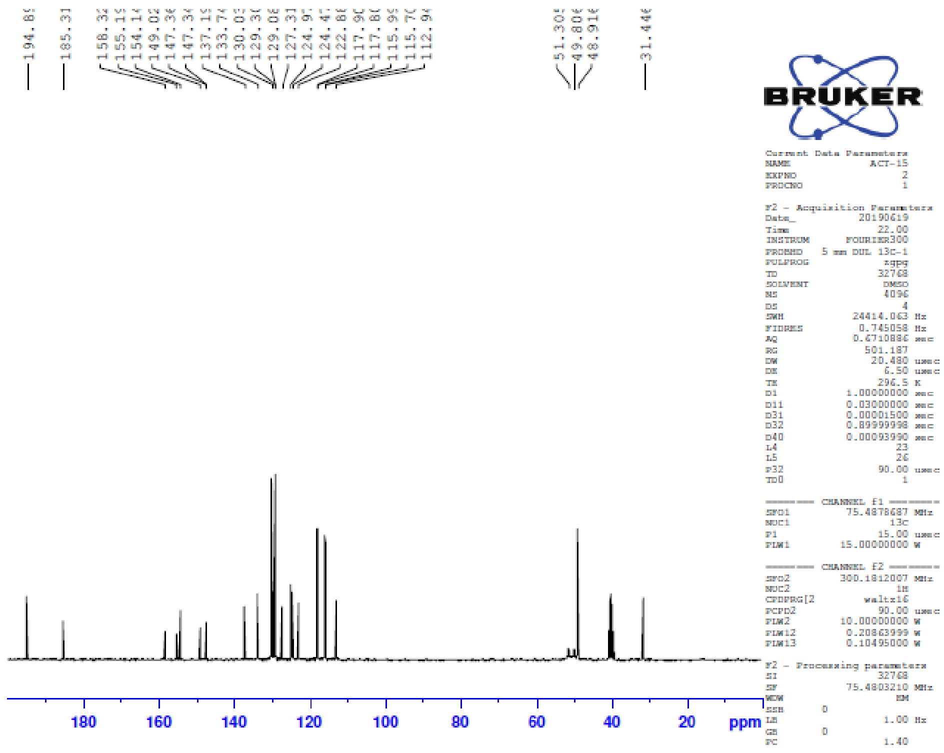

Figure S72. *Compound **4o**-*[1]*H-NMR spectrum*

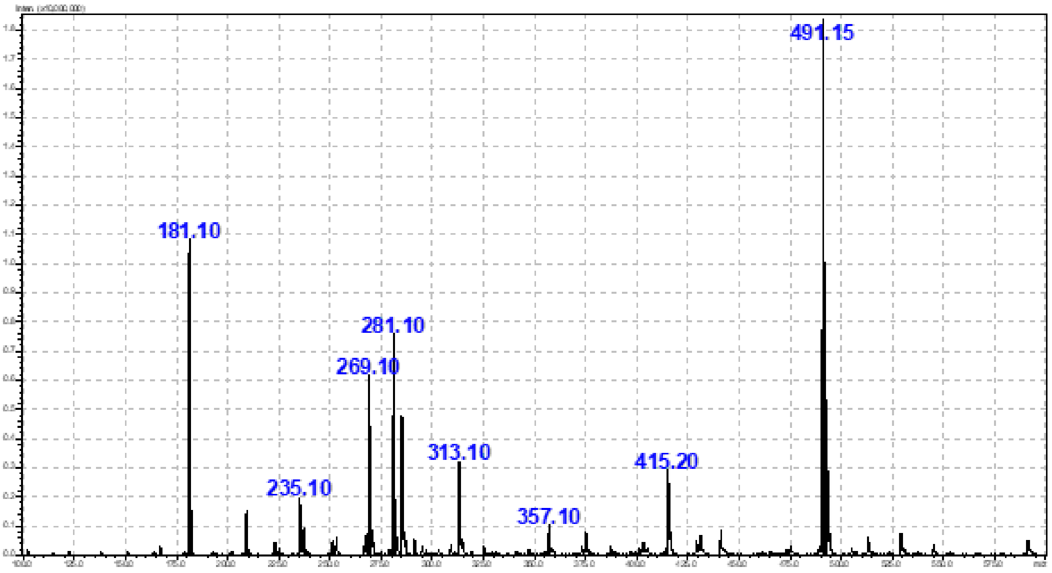

Figure S73. *Compound **4o**-LC-MSMS spectrum*
